# Structural Analysis of Oxidized Cerebrosides from the Extract of Deep-Sea Sponge *Aulosaccus* sp.: Occurrence of Amide-Linked Allylically Oxygenated Fatty Acids

**DOI:** 10.3390/molecules25246047

**Published:** 2020-12-21

**Authors:** Elena A. Santalova, Vladimir A. Denisenko, Pavel S. Dmitrenok

**Affiliations:** G.B. Elyakov Pacific Institute of Bioorganic Chemistry, Far Eastern Branch of the Russian Academy of Sciences, Pr. 100 let Vladivostoku 159, 690022 Vladivostok, Russia; vladenis@piboc.dvo.ru (V.A.D.); paveldmt@piboc.dvo.ru (P.S.D.)

**Keywords:** glycosphingolipids, cerebrosides, peroxidation products, structure elucidation, allylic thioether, NMR, ESI-MS, GC-MS, mass spectra, glass sponge

## Abstract

The structural elucidation of primary and secondary peroxidation products, formed from complex lipids, is a challenge in lipid analysis. In the present study, rare minor oxidized cerebrosides, isolated from the extract of a far eastern deep-sea glass sponge, *Aulosaccus* sp., were analyzed as constituents of a multi-component RP-HPLC (high-performance liquid chromatography on reversed-phase column) fraction using NMR (nuclear magnetic resonance) spectroscopy, mass spectrometry, GC (gas chromatography), and chemical transformations (including hydrogenation or derivatization with dimethyl disulfide before hydrolysis). Eighteen previously unknown β-D-glucopyranosyl-(1→1)-ceramides (**1a**–**a^//^**, **1b**–**b^//^**, **2a**–**a^//^**, **2b**–**b^//^**, **3c**–**c^//^**, **3d**–**d^//^**) were shown to contain phytosphingosine-type backbones (2*S*,3*S*,4*R*,11*Z*)-2-aminoeicos-11-ene-1,3,4-triol (in **1**), (2*S*,3*S*,4*R*,13*Z*)-2-aminoeicos-13-ene-1,3,4-triol (in **2**), and (13S*,14R*)-2-amino-13,14-methylene-eicosane-1,3,4-triol (in **3**). These backbones were *N*-acylated with straight-chain monoenoic (2*R*)-2-hydroxy acids that had allylic hydroperoxy/hydroxy/keto groups on C-17**^/^** in the 15**^/^***E*-23:1 chain (**a**–**a^//^**), C-16**^/^** in the 17**^/^***E*-23:1 (**b**–**b^//^**) and 14**^/^***E*-22:1 (**c**–**c^//^**) chains, and C-15**^/^** in the 16**^/^***E*-22:1 chain (**d**–**d^//^**). Utilizing complementary instrumental and chemical methods allowed for the first detailed structural analysis of a complex mixture of glycosphingolipids, containing allylically oxygenated monoenoic acyl chains.

## 1. Introduction

Lipid hydroperoxides are labile compounds derived from lipids containing carbon-carbon double bonds. The formation of these primary peroxidation products occurs in enzymatic and non-enzymatic (autooxidation, photo-oxidation) reactions [1]. Lipid hydroperoxides, formed in biological systems, have not only multiple damaging effects on cellular macromolecules, but are also important regulators of many cellular processes [2]. In some pathological situations, lipid hydroperoxides are generated at higher than normal rates. Such overproduction is implicated in several human diseases and exposures including atherosclerosis, cancer, diabetes, acute lung injury, chronic alcohol exposure, and neurodegenerative disorders. The complex nature of lipid peroxidation and its potential biological significance have attracted the attention of scientists across many disciplinary fields, ranging from chemistry and biochemistry to biology and clinical science (see review [3] and references cited herein).

Recent progress in the characterization and quantification of oxygenated fatty acids (FAs) has facilitated a better understanding of lipid oxidation, but the methods currently available still suffer from unresolved sensitivity and selectivity problems [4]. The main reason for these problems is the complexity of the product profile formed by oxidation from a single lipid molecular species. In particular, peroxidation of any unsaturated fatty acyl group may generate a mixture of allylic hydroperoxides with different double bond positions and/or configurations. These hydroperoxides are easily decomposed or go through further reactions to form different secondary peroxidation products including epoxides, allylic alcohols, α,β-unsaturated ketones (enones), and chain-cleavage products [1]. The constituents of these mixtures are difficult to separate, isolate, and identify. Additionally, natural extracts are characterized, as a rule, by extremely low levels of oxidized lipids, causing further difficulties in their isolation and analysis [3,4]. Undoubtedly, structural elucidation of primary and secondary peroxidation products, formed from complex lipids, remains a challenge for lipid analysts.

Monoenes are much less prone to undergo oxidation than polyenes. Respectively, direct addition of peroxyl radicals to monounsaturated lipids is not generally observed with the exceptions of cholesterol [3] and related sterols. In particular, sphingolipids, containing mainly saturated or monounsaturated hydrocarbon chains, are poor substrates for peroxidation. We found only one report on the isolation of peroxidized sphingolipids from a natural source, but these compounds formed due to oxidation of unique sphingoid base moiety with conjugated double bonds. Namely, some stereoisomeric glycosphingolipids, containing endoperoxide and allylic hydroperoxide functions in their dienoic sphingoid base moieties, were isolated from the extract of the sponge *Axinella corrugate* [5]. The locations of the double bonds and peroxide-containing groups were determined by ^1^H,^1^H-COSY (proton-proton correlation spectroscopy), НМВС (heteronuclear multiple-bond correlation spectroscopy), and ROESY (rotating-frame nuclear Overhauser effect spectroscopy) experiments due to the proximity of the previously mentioned functionalities to the polar portion of the sphingolipids. Methanolysis (MeOH/HCl) of the peroxidized glycosphingolipids was used to release methyl esters of saturated FAs, but oxidized dienoic sphingoid bases were not obtained, apparently, due to rapid decomposition under acidic conditions.

**Figure 1:**
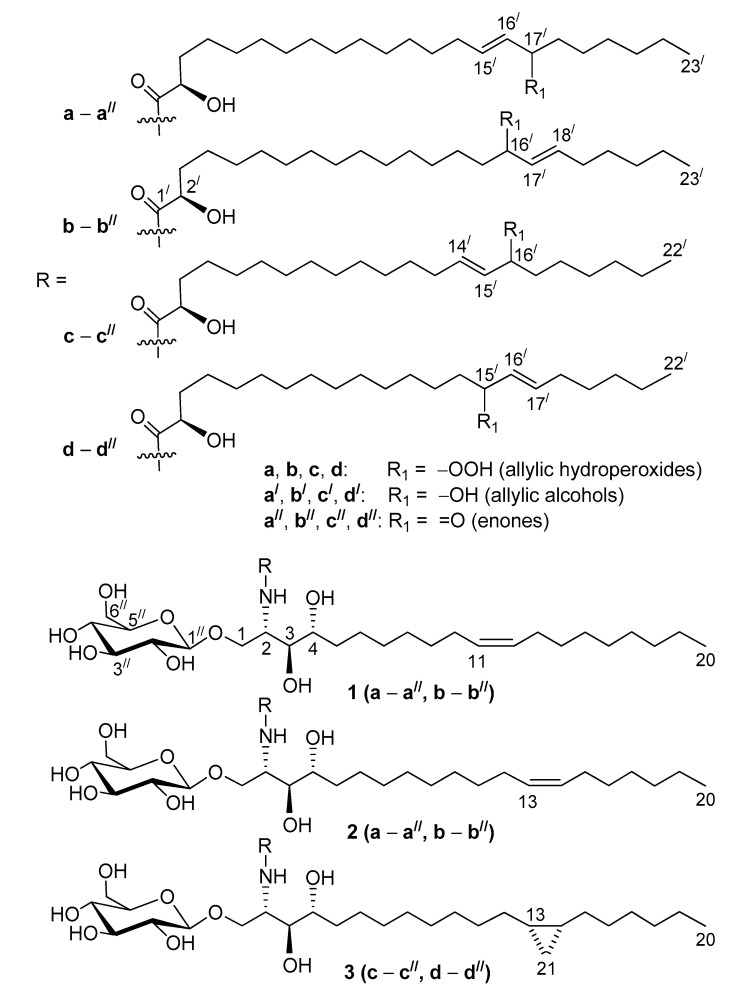
Oxidized cerebrosides from the extract of a sponge *Aulosaccus* sp.

In mass spectrometric (MS) studies of oxidative stress markers (disease biomarkers), oxidation of some standard glycosphingolipids, containing monoenoic FAs, was induced by a Fenton reaction (H_2_O_2_/FeCl_2_) or UVA (ultraviolet A) irradiation [6,7]. In particular, Couto et al. obtained galactosylceramides with allylic hydroperoxy, hydroxy, or keto groups in FA chains. These cerebrosides were characterized using ESI-MS (electrospray ionization mass spectrometry) and HPLC-MS (high-performance liquid chromatography-mass spectrometry) methods (including tandem mass spectrometry (MS/MS)) [6]. In our continued study on lipids from far eastern marine sponges [8], similar oxidized cerebrosides (Figure 1), presumably derived from glucosylceramides with monounsaturated fatty acyl groups, were found in the extract of a deep-sea glass sponge, *Aulosaccus* sp. As seen in Figure 1, the depicted compounds contain an allylic hydroperoxy, hydroxy, or keto group in each acyl chain, but their monoenoic sphingoid base moieties are not oxidized. A fraction of these minor cerebrosides, along with fractions of non-oxidized cerebrosides [8], was isolated from the sponge extract using high-performance liquid chromatography on reversed-phase column (RP-HPLC). The present study presents structural elucidation of rare oxidized glycosphingolipids using mass spectrometry (ESI-MS and gas-chromatography-mass spectrometry (GC-MS)), ^1^Н-, ^13^С-NMR (nuclear magnetic resonance) spectroscopy, GC, chemical transformations, and optical rotation measurement.

A classical approach to the analysis of any complex lipid includes methanolysis or hydrolysis, which is followed by analyses of liberated derivatized simple lipids and sugar. However, prolonged high temperatures and treatments with acidic or alkaline solutions, required for solvolysis of *N*-acyl lipids, presents significant problems with respect to the potentially labile allylic oxygen-containing groups of the acyl chains. To solve these problems, we used catalytic hydrogenation to fix the starting positions of the oxygenated groups prior to chemical degradation of the oxidized glycosphingolipids. In addition, attempts were made to fix the double bonds of allylic substructures by reacting with dimethyl disulfide (DMDS) because, in our preliminary research, the DMDS adduct of methyl oleate did not lose *S*-methyl groups during hydrolysis with HCl in MeCN-H_2_O. To minimize possible allylic rearrangements (1,3-isomerizations) and other alterations, we avoided elevated temperatures and strong acid/base conditions in the derivatization reactions before hydrolysis. Thus, our attention was mainly focused on procedures suitable for an initial detailed structural analysis of a complex mixture of glycosphingolipids, containing an allylic hydroperoxy, hydroxy, or keto group in the monoenoic acyl chain.

## 2. Results and Discussion

Isomeric allylic hydroperoxides **1a**, **1b**, **2a**, **2b**, **3c**, **3d**, related isomeric allylic alcohols **1a^/^**, **1b^/^**, **2a^/^**, **2b^/^**, **3c^/^**, **3d^/^**, and isomeric enones **1a**^//^, **1b^//^**, **2a^//^**, **2b^//^**, **3c^//^**, **3d^//^** (Figure 1) were constituents of a single RP-HPLC fraction. In the beginning of this study, the fraction contained 46% allylic hydroperoxides, 43% allylic alcohols, and 11% enones, calculated using relative intensities of the characteristic, isolated ^1^Н-NMR signals of *trans*-olefinic protons (see below). The final results of this study yielded 25%, 54%, and 21% of these allylically oxygenated compounds, respectively. Therefore, further transformation of allylic hydroperoxides to allylic alcohols and enones occurred upon analysis (registrations of NMR and MS spectra, preparation of samples, storage, etc.).

In UPLC-MS (ultra-performance liquid chromatography–mass spectrometry) analysis of oxidized cerebrosides, base peak chromatogram, and extracted-ion chromatograms provided limited information because many components eluted simultaneously. In particular, peaks for major isomeric allylic hydroperoxides **1a** and **1b** were not well-resolved (Supplementary Materials, Figure S1a,b).

### 2.1. Positive and Negative Ion Mode ESI-MS/MS Analyses of Oxidized Cerebrosides

**Scheme 1:**
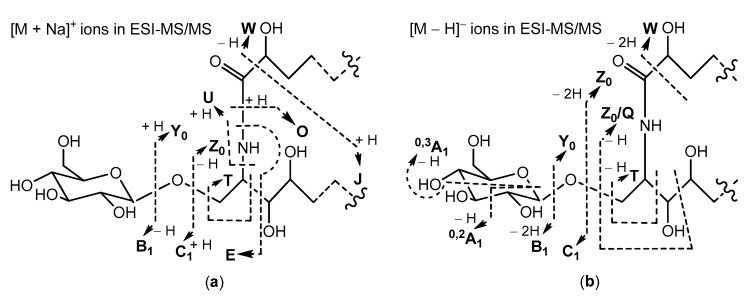
Designations of the fragment ions observed in the ESI-MS/MS spectra of (**a**) [M + Na]^+^ and (**b**) [M − H]^−^ ions of the cerebrosides reported here. These designations are consistent with the ones proposed by Ann and Adams [9]. In addition, [*Z*_0_/*Q* − C_3_H_5_N (55 Da)] ions (not shown) were observed in the MS/MS spectra of the [M − H]^–^ ions. The fragment *Z*_0_/*Q* was also referred to as a *Z*_0_/*K* ion [10].

The molecular formulae, С_49_H_93_NO_12_ for allylic hydroperoxides (**1a**, **1b**, **2a**, **2b**, **3c**, **3d**), С_49_H_93_NO_11_ for allylic alcohols (**1a^/^**, **1b^/^**, **2a^/^**, **2b^/^**, **3c^/^**, **3d^/^**), and С_49_H_91_NO_11_ for enones (**1a**^//^, **1b^//^**, **2a^//^**, **2b^//^**, **3c^//^**, **3d^//^**), were determined by HR-ESI-MS (high resolution ESI-MS) analyses in positive-((+)ESI-MS) and negative-((–)ESI-MS) ion modes. Complementary (+)ESI- and (–)ESI-MS/MS analyses of these glucosylceramides, containing 2-hydroxy acyl chains and phytosphingosine-type backbones, resulted in a series of fragment ions, as shown in Scheme 1.

**Figure 2:**
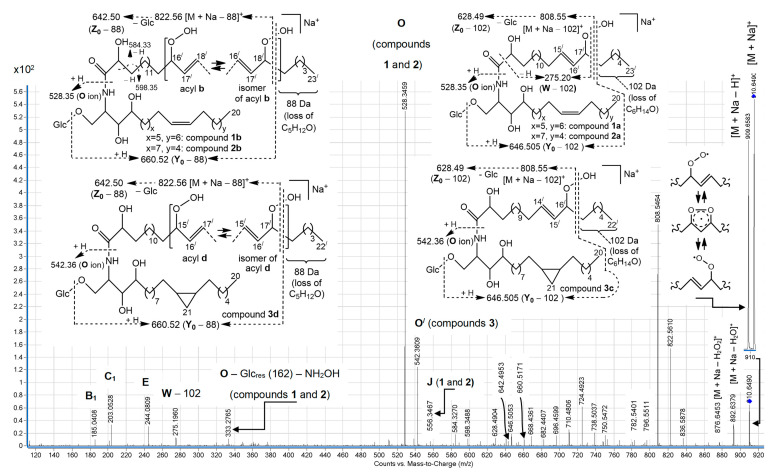
(+)ESI-MS/MS spectrum of [M + Na]^+^ ion of isomeric allylic hydroperoxides **1a**, **1b**, **2a**, **2b**, **3c**, and **3d**.

Many of the fragment ions, shown in Scheme 1, have also been detected in our ESI-MS/MS studies of non-oxidized cerebrosides isolated from *Aulosaccus* sp. Namely, (+)ESI-MS/MS spectra of sodium adducts from non-oxidized glycosphingolipids have been characterized by prominent peaks, corresponding to [M + Na]^+^ (base peak), *Y*_0_, *Z*_0_, *O*, and *C*_1_ ions, and by small peaks, representing [M + Na − H_2_O]^+^, *E*, and *B*_1_ ions. However, the (+)ESI-MS/MS spectrum of the [M + Na]^+^ ion of isomeric hydroperoxy cerebrosides (Figure 2) showed other relative abundances for a variety of these ions. In particular, the *O* ions of *m*/*z* 528.35, representing isomeric monoglucosylated monounsaturated С_20_ sphingoid base backbones **1** and **2**, constituted the base peak of this spectrum. The spectrum also exhibited a homologous, less abundant *O***^/^** ion (*m*/*z* 542.36), containing a monoglucosylated cyclopropane С_21_ sphingoid base backbone **3**. A relatively low intensity pseudo-molecular ion peak (*m*/*z* 910.65, [M + Na]^+^) and very small peaks, corresponding to *Y*_0_ (*m*/*z* 748.60) and *Z*_0_ (*m*/*z* 730.58) ions (not shown), were observed. The presence of the [M + Na − Н]^+^ peak, comparable with the pseudo-molecular ion peak of the hydroperoxides, was explained by hydrogen atom abstraction, followed by electronic delocalization in the resulting radical, which might yield rearranged products. At the same time, the (+)ESI-MS/MS spectrum revealed more abundant [M + Na − 102]^+^ (*m*/*z* 808.55), [M + Na − 88]^+^ (*m*/*z* 822.56), [*Y*_0_ − 102] (*m*/*z* 646.505), [*Y*_0_ − 88] (*m*/*z* 660.52), [*Z*_0_ − 102] (*m*/*z* 628.49), and [*Z*_0_ − 88] (*m*/*z* 642.50) ions. These fragments and fragment [*W* − 102] (*m*/*z* 275.20), each with a terminal α,β-unsaturated aldehyde, were thought to arise from specific α-cleavages of lipid hydroperoxides (Figure 2). Related fragment ions, formed by C_n_H_2n+2_O losses from [M + Na]^+^ ions, were also found in the MS/MS studies of some monoenoic [6] and polyenoic [11,12,13] FA moieties or free FAs, in which an allylic hydroperoxy group was between double bond(s) and a terminal methyl group. In general, the [M + Na − C_n_H_2n+2_O]^+^ ions were also more abundant in MS/MS spectra of those compounds compared to their [M + Na]^+^ ions.

Ions, possibly formed by Hock cleavage [14,15], were insignificant in our (+)ESI-MS/MS analysis of allylic hydroperoxides. These fragments included *m*/*z* 796.55 (for **1b**, **2b**, and **3d**) and 782.54 (for the allylic isomers of **1a**, **2a**, and **3c**) ions, as illustrated in Scheme S1 (Supplementary Materials) and Figure 2 (allylic rearrangements for acyl chains **a** and **c** are not shown). In contrast, the relatively abundant ions, presumably formed by analogous cleavage from hemiacetal derivatives, were reported for MS/MS fragmentations of [M + Na]^+^ precursors of some free polyenoic FAs in which an allylic hydroperoxy group was between the double bond system and C-1 [12,13].

**Scheme 2:**
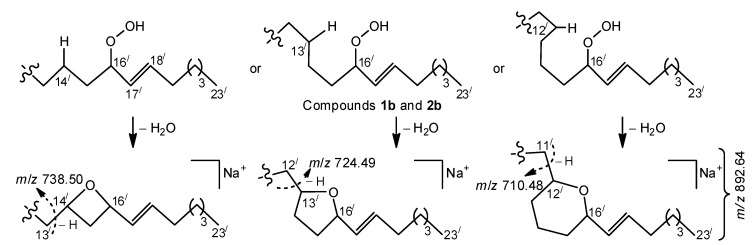
Possible formation of the homologous ions of *m*/*z* 738.50, 724.49, and 710.48.

Favorable cleavage of the isomers, formed by allylic rearrangements of acyl chains **b** and **d** (Figure 2), occurred, losing an 88 Da fragment. At the same time, compounds with parent acyl chains **b** and **d** underwent other favorable fragmentations, yielding a distinct group of homologous ions (from *m*/*z* 668.44 to 738.50), containing the most significant fragment of *m*/*z* 724.49. We suggest that the occurrence of these ions may be connected with homolysis of a weak RO-/-OH bond, formation of an alkoxyl radical, and subsequent formation of a radical centered on a remote and non-activated carbon atom of a saturated hydrocarbon chain. This may lead to fast cyclization that, in turn, leads to MS fragmentations of the resulting cyclic ethers, shown in Scheme 2. The proposed cyclization reaction is reminiscent of the formation of cyclic (mainly, five-membered) ethers from acyclic saturated monohydroxy alcohols, occurring via radical (primarily alkoxyl) intermediates under appropriate chemical and thermal conditions (for reviews, see References [16,17]). In this process, secondary aliphatic alcohols yielded 2,5-dialkyltetrahydrofurans [18]. A possible mechanism for the formation of such products includes 1,5-transposition of the radical center from the oxygen atom of the alkoxyl radical to *δ*-carbon atom, involving a 1,5-hydrogen transfer through a chair-like six-membered transition state. For a linear hydrocarbon chain with an initial alkoxyl radical, the 1,5-hydrogen atom transfer may be considered the most common reaction, even though intramolecular abstractions of the hydrogen atom from other positions (1,4-migrations, 1,6-migrations, and 1,7-migrations, etc.) may be observed [17]. Similar processes may have occurred in our MS/MS experiment because alkyl hydroperoxides are known to form alkoxyl radicals by thermal or photolytic decomposition [17,19]. Apparently, the ability to undergo favorable cyclic transition states affected the fragmentation process, leading to the formation of major homologous ions, as illustrated in Scheme 2.

**Figure 3:**
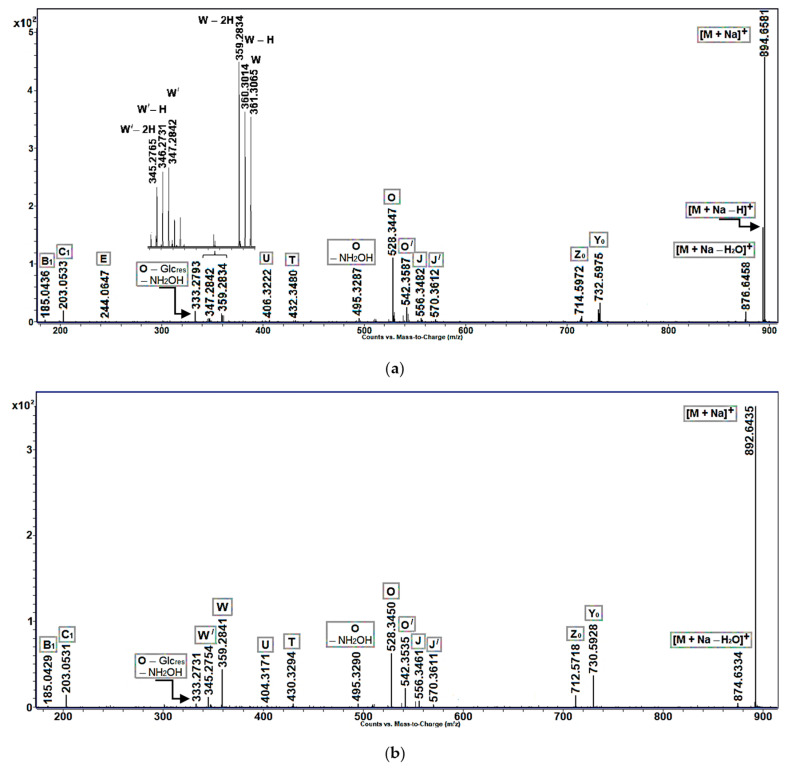
(+)ESI-MS/MS spectra of [M + Na]^+^ ions of isomeric (**a**) allylic alcohols **1a^/^**, **1b^/^**, **2a^/^**, **2b^/^**, **3c^/^**, **3d^/^** and (**b**) enones **1a**^//^, **1b^//^**, **2a^//^**, **2b^//^**, **3c^//^**, **3d^//^**.

Ion intensity profiles, obtained for cerebrosides with allylic hydroxy or keto groups, were, in general, similar to those of non-oxidized cerebrosides found in *Aulosaccus* sp. In particular, the MS/MS spectra of sodiated molecular ions of allylic alcohols (Figure 3a: *m*/*z* 894.66 [M + Na]^+^) and enones (Figure 3b: *m*/*z* 892.64 [M + Na]^+^) showed predominant peaks of pseudo-molecular ions, along with peaks of lower intensities, which represented [M + Na − H_2_O]^+^, *Y*_0_, *Z*_0_, and *O* ions. However, unlike [M + Na]^+^ ions of non-oxidized cerebrosides, the sodium adducts of allylic alcohols and enones fragmented to give discernible ions of *W*-type and minor *U* and *T* ions. Additionally, the acyl-containing ions of allylic alcohols had a tendency to lose one, or even two, hydrogen atoms. In this case, a trend was observed toward the increased loss of hydrogen atoms with decreasing ion masses. For example, a significant difference was noted between the relative intensities of [M + Na]^+^ and [M + Na − H]^+^ peaks ([M + Na − 2H]^+^ ions were not even detected), but the intensities of *W*, [*W* − H], and [*W* − 2H] peaks were comparable (Figure 3a).

**Figure 4:**
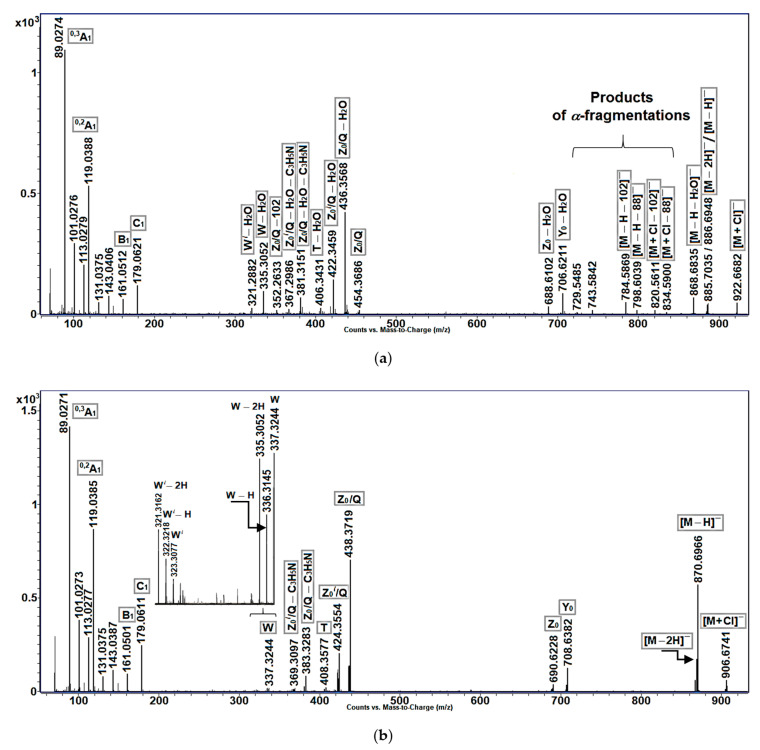

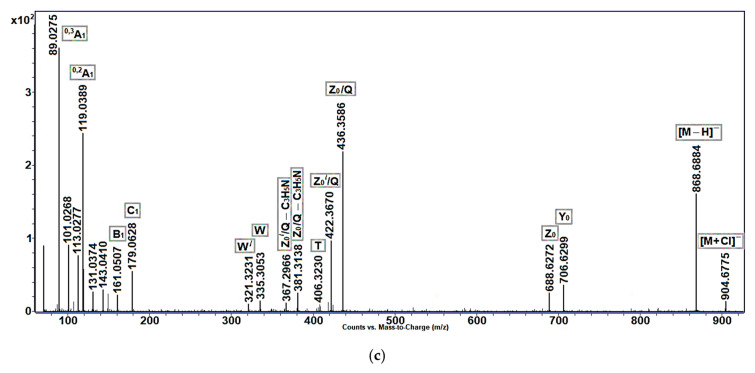
(−)ESI-MS/MS spectra of [M + Cl]^−^ and [M − H]^−^ ions of isomeric (**a**) allylic hydroperoxides **1a**, **1b**, **2a**, **2b**, **3c**, **3d**, (**b**) allylic alcohols **1a^/^**, **1b^/^**, **2a^/^**, **2b^/^**, **3c^/^**, **3d^/^**, and (**c**) enones **1a**^//^, **1b^//^**, **2a^//^**, **2b^//^**, **3c^//^**, **3d^//^**.

In (−)ESI-MS/MS experiments with [M − H]^−^ and [M + Cl]^−^ ions (Figure 4a–c), allylic hydroperoxides again produced more fragments than allylic alcohols and enones. The main feature of (−)ESI-MS/MS fragmentation of [M − H]^−^, *Y*_0_, *Z*_0_, *Z*_0_/*Q*, *T*, *W*, and other precursor ions, containing an allylic hydroperoxy group, was the loss of water to produce fragments with enone functionality in acyl chains, as described for [M − H]^−^ ions of hydroperoxy-eicosatetraenoic acids [20]. In particular, the homologous ions of *m*/*z* 436.4 ([*Z*_0_/*Q* − H_2_O]) and 422.4 ([*Z*_0_**^/^**/*Q* − H_2_O]), containing acyl C_23_ and С_22_ chains, respectively (Figure 4a), were also observed in (−)ESI-MS/MS spectrum of enones (Figure 4c). Then, relatively low-intensity pseudo-molecular ion peaks ([M + Cl]^−^ and [M − H]^−^) and very small peaks, corresponding to *Y*_0_ and *Z*_0_ ions (not shown), were seen in the (−)ESI-MS/MS spectrum of allylic hydroperoxides. A discernible [M − 2H] peak was comparable with a pseudo-molecular [M − H] peak. This spectrum also exhibited [M + Cl − 102]^−^, [M + Cl − 88]^−^, [M − H − 102]^−^, [M − H − 88]^−^, and [*Z*_0_/Q − 102] ions, interpreted as α-cleavage ions with a terminal α,β-unsaturated aldehyde. Additionally, the two minor ions of *m*/*z* 743.6 and 729.55 could be formed by α-cleavages of compounds in which an allylic hydroperoxy group was between a double bond and C-1^/^. In particular, the *m*/*z* 743.6 ions could be fragments of isomeric compounds **1b**, **2b** (C-15^/^−C-16^/^ bond fission), and **3d** (C-14^/^−C-15^/^ bond fission), while the less abundant *m*/*z* 729.55 ions could be fragments of the allylic isomers of compounds **1a**, **2a** (C-14^/^−C-15^/^ bond fission), and **3c** (C-13^/^−C-14^/^ bond fission).

Like the (−)ESI-MS/MS spectra of the non-oxidized cerebrosides of *Aulosaccus* sp., those of allylic alcohols (Figure 4b) and enones (Figure 4c) exhibited significant peaks corresponding to [M − H]^−^ and *Z*_0_/*Q* ions, with lower intensity peaks representing [M + Cl]^−^, *Y*_0_, *Z*_0_, [*Z*_0_/*Q* − C_3_H_5_N], and *W* ions. The negatively charged acyl-containing ions of allylic alcohols (Figure 4b), like their positively charged acyl-containing counterparts (Figure 3a), tended to lose hydrogen atoms in the MS/MS experiment.

### 2.2. NMR Characterization of Oxidized Cerebrosides

The ^1^Н-NMR and ^13^С-NMR spectra (CD_3_OD) of oxidized cerebrosides (Table 1, Figures S2 and S3), as well as the corresponding spectra of the non-oxidized cerebrosides of *Aulosaccus* sp. [8], showed signals of β-glucopyranosyl-(1→1)-ceramides that had monoenoic or cyclopropane-containing phytosphingosine-type backbones, *N*-acylated with 2-hydroxy FAs. In particular, H-2 (*δ*_Н_ 4.25, m) of the sphingoid base moieties displayed a characteristic cross signal with *N*-acyl C-1^/^ (*δ*_C_ 177.7), as demonstrated by an НMBC experiment with sphingolipids **1**–**3**. ^1^Н,^1^Н-COSY diagram indicated that several protons, starting from −O−CH_2_− (*δ*_Н_ 3.80 and 4.045, dd, CH_2_-1) and ending with alkyl −CH_2_− (*δ*_Н_ 1.31 and 1.55, m, CH_2_-6), formed a linear spin system of phytosphingosine-type moieties of **1**–**3**. Another spin system consisted of CH-2^/^ (*δ*_Н_ 4.01, dd), CH_2_-3^/^ (*δ*_Н_ 1.60 and 1.74, m), and CH_2_-4^/^ (*δ*_Н_ 1.42, m) protons of 2-hydroxy acyl chains. The signals of a β-glucopyranoside moiety were sequentially assigned by ^1^Н,^1^Н-COSY, HSQC (heteronuclear single-quantum correlation spectroscopy), and НМВС experiments, starting from the signal of anomeric CH-1^//^ (*δ*_Н_ 4.28, d, *J* = 7.8 Hz; *δ*_C_ 105.3). Accordingly, cross signals C**Н_2_**-1/**С**-1^//^ and C**Н**-1^//^/**С**-1 were observed in the НМВС diagram of glycosides **1**–**3**. Then, the ^1^Н- and ^13^С-NMR spectra of these sphingolipids showed signals of long hydrocarbon chains (−(CH_2_)_n_−, *δ*_Н_ 1.22–1.42, m, *δ*_С_ 30.6–32.0) and terminal methyl groups (*δ*_Н_ 0.89–0.90, several overlapping triplets, *δ*_С_ 15.0–15.05, broad signal). The *δ* values of allylic CH_2_ (*δ*_C_ 28.7–28.85, *δ*_Н_ 2.02, m) and olefinic CH (*δ*_Н_ 5.34, m) were used to characterize *cis*-double bonds of backbones **1** and **2**. These data were in good agreement with ^1^Н-NMR and ^13^С-NMR (CD_3_OD) data on some cerebrosides [21] and FA standards [22] with isolated *cis*-double bonds (*cis*-isomers: *δ*_C_ 28.7–28.9, *δ*_Н_ 2.02–2.03 m, allylic CH_2_, and *δ*_H_ 5.335–5.34 m, olefinic CH, *trans*-isomers: *δ*_C_ 34.15–34.2, *δ*_Н_ 1.97–1.975 m, allylic CH_2_, and *δ*_Н_ 5.37–5.38 m, olefinic CH). A *cis*-cyclopropane ring in backbone **3** caused three upfield shifted signals at *δ*_Н_ −0.33 (dt, *J* = 4.1, 5.3 Hz, H-21a), 0.58 (ddd, *J* = 4.1, 8.3, 8.3 Hz, H-21b), and 0.67 (m, H-13, H-14).

**Figure 5:**
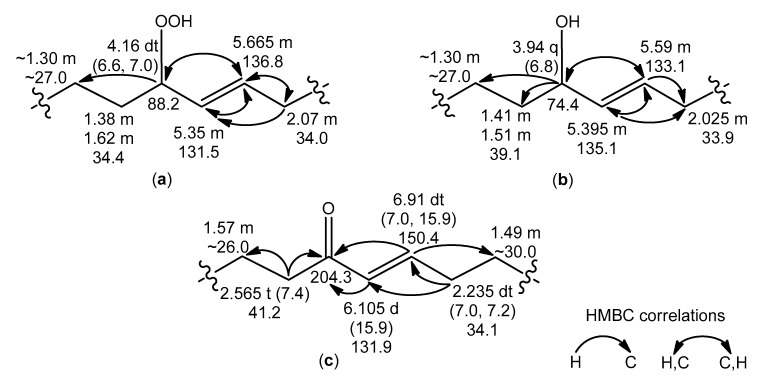
The *δ*_H_ (500 MHz) and *δ*_C_ (125 MHz) values and key HMBC correlations for acyl fragments with allylic (**a**) hydroperoxy (acyl chains **a**–**d**), (**b**) hydroxy (acyl chains **a^/^**–**d^/^**), and (**c**) keto (acyl chains **a^//^**–**d^//^**) groups (solvent: CD_3_OD).

Apart from the previously mentioned NMR resonances, the signals of *trans*-monoenoic acyl moieties with an allylic hydroperoxy, hydroxy, or keto group (Figure 5a–c) were observed in the NMR spectra of the RP-HPLC fraction, containing oxidized cerebrosides. The *δ*_H_ values (CD_3_OD) of the signals for –**H**C(OOH)– (4.16, dt), –**H**C(OH)– (3.94, q), and −CH=C**H**–CO– (6.105, d) were close to *δ*_H_ values (CDCl_3_) of signals of corresponding protons in the *trans*-monoenoic allylic hydroperoxides (4.2, q), allylic alcohols (4.0, q), and enones (6.1, d), respectively, prepared from oleic acid [23]. Deviations of *δ*_H_ values (this study) from those in other experiments [23] arose from solvent effects. According to ^1^Н,^1^Н-COSY correlations, a spin system, assigned to acyl chains **a**–**d** (Figure 5a), included protons of allylic hydroperoxide, especially two olefinic CH (*δ*_Н_ 5.35 and 5.665, m) and allylic CH (*δ*_Н_ 4.16, dt, *J* = 6.6, 7.0 Hz), bearing −OOH. Allylic CH (*δ*_Н_ 3.94, q, *J* = 6.8 Hz), bearing –OH, and two olefinic CH (*δ*_Н_ 5.395 and 5.59, m) groups, belonging to allylic alcohol, were part of a spin system within acyl chains **a^/^**–**d^/^** (Figure 5b). In experiments, involving selective irradiation of allylic protons, the coupling constants *J* = 15.6 Hz and *J* = 15.4 Hz for the *trans*-olefinic protons of the allylic hydroperoxides and allylic alcohols, respectively, were detected. The characteristic NMR resonances of two *trans*-alkenyl CH (*δ*_Н_ 6.105, d, *J* = 15.9 Hz, and 6.91, dt, *J* = 7.0, 15.9 Hz), conjugated C=O (*δ*_С_ 204.3), and α-CH_2_ (*δ*_Н_ 2.565, t, *J* = 7.4 Hz) groups were used for determining substructures of enones in acyl chains **a^//^**–**d^//^** (Figure 5c). The structures of the allylic hydroperoxides, allylic alcohols, and enones were confirmed by HMBC correlations, as depicted in Figure 5.

**Figure A1:**
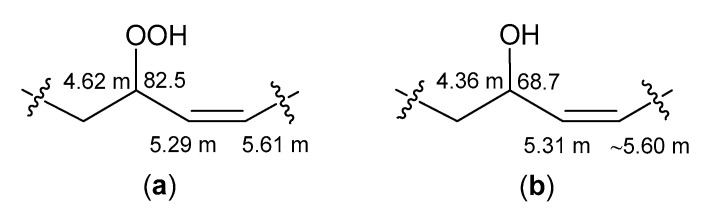
Some *δ*_H_ (500 MHz) and *δ*_C_ (125 MHz) values for trace *cis*-monoenes with allylic (**a**) hydroperoxy or (**b**) hydroxy groups (solvent: CD_3_OD).

Some very weak signals in the ^1^Н-NMR, ^1^Н,^1^Н-COSY, and HSQC spectra of oxidized cerebrosides found in the present study were attributed to *cis*-double bonds of allylic hydroperoxides and allylic alcohols (Appendix A, Figure A1). The complete structures of these compounds could not be elucidated due to their trace amounts.

### 2.3. Analyses of FAs, Sphingoid Bases, and Sugar Obtained from Oxidized Cerebrosides

The RP-HPLC oxidized cerebroside fraction was divided into two parts (parts 1 and 2), which were treated using different chemical procedures before hydrolysis. Then, we applied MeCN/HCl hydrolysis [24] for chemical degradation of cerebrosides. In our experience [8], this procedure causes less disruption of spingoid bases than methanolysis (MeOН/HCl), which is most widely used in studies of complex lipids.

*Analysis of FAs from Part 1*. Part 1 of the oxidized cerebrosides was subjected to hydrogenation (with Adams’ catalyst) to fix the positions of allylic oxygen-containing groups before hydrolysis. Upon hydrolysis, liberated FAs were acetylated and methylated. The ^1^Н-NMR spectrum (CDCl_3_) of FA derivatives showed proton signals of mid-chain substructures, including *−***H**_2_C−CO−C**H**_2_− at *δ*_Н_ 2.38 (t, *J* = 7.4 Hz) and −С**Н**(OAс)– at *δ*_H_ 4.855 (m). GC-MS analysis (electron impact ionization) of these derivatives revealed methyl esters of 2-acetyloxy C_23_ and C_22_ acids, containing an isolated keto or acetyloxy group or no additional oxygenated group. Similar products, namely keto, hydroxy, and non-oxygenated acid derivatives, have been previously reported for the hydrogenation (in EtOH over Adams’ catalyst) of allylic 9-hydroperoxides and 10-hydroperoxides, obtained from methyl oleate [25].

In the present report, mass spectra exhibited base peaks at *m*/*z* 339 and 325 ([M − MeOCO − CH_2_CO]^+^) for methyl esters of 2-acetyloxy keto C_23_ (440 [M]^+^) and C_22_ (426 [M]^+^) acids, respectively (Figures S4–S7). Ions produced by cleavage β to a keto group and ions formed from the methyl end of the molecules by cleavage α to the keto group were prominent in the mass spectra, as described for MS fragmentations of several methyl esters of oxo (keto) FAs [26]. In particular, mass spectra of methyl esters of C_23_ and C_22_ acids displayed homologous pairs of *m*/*z* 127/142 and 113/128 fragments, containing methyl ends of the molecules with keto groups on the (*n*–8) or (*n*–7) carbon, respectively. These keto group positions were confirmed by a number of ions containing the polar end of the FA esters that also produced daughter ions due to loss of AcOH, CH_2_CO, or MeOH (Figures S4–S7).

Hydrogenation of part 1, followed by hydrolysis, acetylation, and methylation, also yielded the methyl esters of 2-acetyloxy C_23_ and C_22_ acids, containing an additional isolated acetyloxy group. Expectedly, [M]^+^ peaks were absent in the mass spectra of the methyl esters of these diacetylated acids, but [M − CH_3_CO]^+^ and [M − AcOH]^+^ ions were observed in high mass regions (Figures S8–S11). Additionally, these compounds, as derivatives of 2-acetyloxy FAs, fragmented to give abundant [M − MeOCO − AcOH]^+^ ions of *m*/*z* 365 and 351 for methyl esters of C_23_ and C_22_ acids, respectively. Isomers, containing an isolated acetyloxy group in different positions, were discerned based on the presence of diagnostic α-cleavage ions and more abundant product ions, formed by elimination of CH_2_CO or AcOH from α-fragments, as described for acetates of secondary alcohols [27]. In particular, the α-fragments included the ions of *m*/*z* 385 and 399 (Figures S8 and S9) for the derivatives of 2,16-, and 2,17-diacetyloxy C_23_ acids, respectively, and the ions of *m*/*z* 371 and 385 (Figures S10 and S11) for the 2,15- and 2,16-diacetyloxy C_22_ acid derivatives, respectively. Moreover, some homologous ions, which arose from C-1–⁄–C-2 bond fission and cleavage α to an isolated acetyloxy group, were specific for the different positions of this group in acyl chains. For example, the abundant ions of *m*/*z* 283 and 297 (Figures S8 and S9), which were detected in the mass spectra of the methyl esters of isomeric 2-acetyloxy C_23_ acids, confirmed the presence of second acetyloxy groups on C-16 and C-17, respectively. Similarly, *m*/*z* 269 and 283 ions in the mass spectra of the methyl esters of 2-acetyloxy C_22_ acids (Figures S10 and S11) indicated a second acetoxy group on C-15 and C-16, respectively. Additionally, α-fragmentation of isomers with an acetyloxy group in the (*n*–8) or (*n*–7) positions and subsequent loss of AcOH gave rise to *m*/*z* 111 and 97 ions, respectively, containing the methyl end of acyl chains.

As a result of the FA analyses, hydrogenated minor derivatives of allylically oxygenated FAs were also detected. While the major derivatives formed from fatty acyl groups containing hydroperoxy/hydroxy/keto groups in the (*n*–8) or (*n*–7) positions, the minor derivatives were rearranged products of these FA moieties (Appendix B, Figures S12–S20).

*Analysis of FAs from Part 2*. In this investigation, we tried to use *S*-methyl groups as markers for oxidized cerebroside double bonds. However, enones with polarized double bonds that did not react with DMDS under mild conditions, and labile allylic hydroperoxides were not suitable for this purpose. Therefore, enones and allylic hydroperoxides were converted into allylic alcohols that were expected to add to DMDS.

**Scheme 3:**
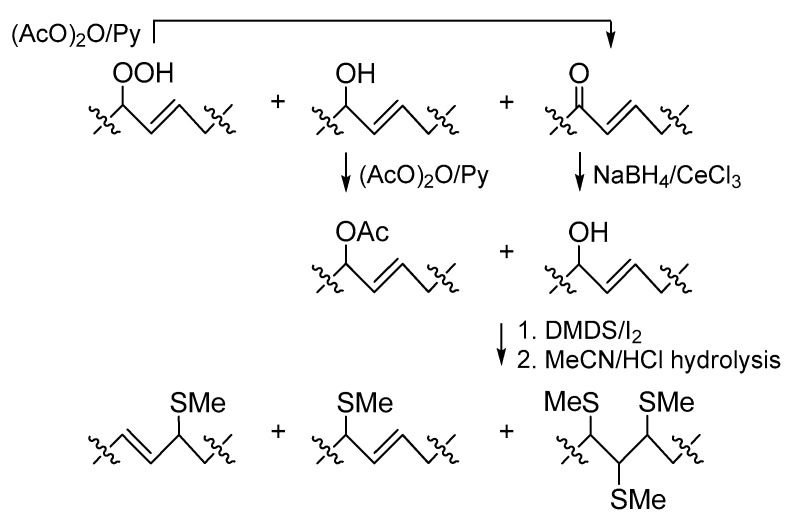
Transformations of acyl substructures, containing allylic hydroperoxy/hydroxy/keto groups, into methylthio derivatives.

**Scheme 4:**
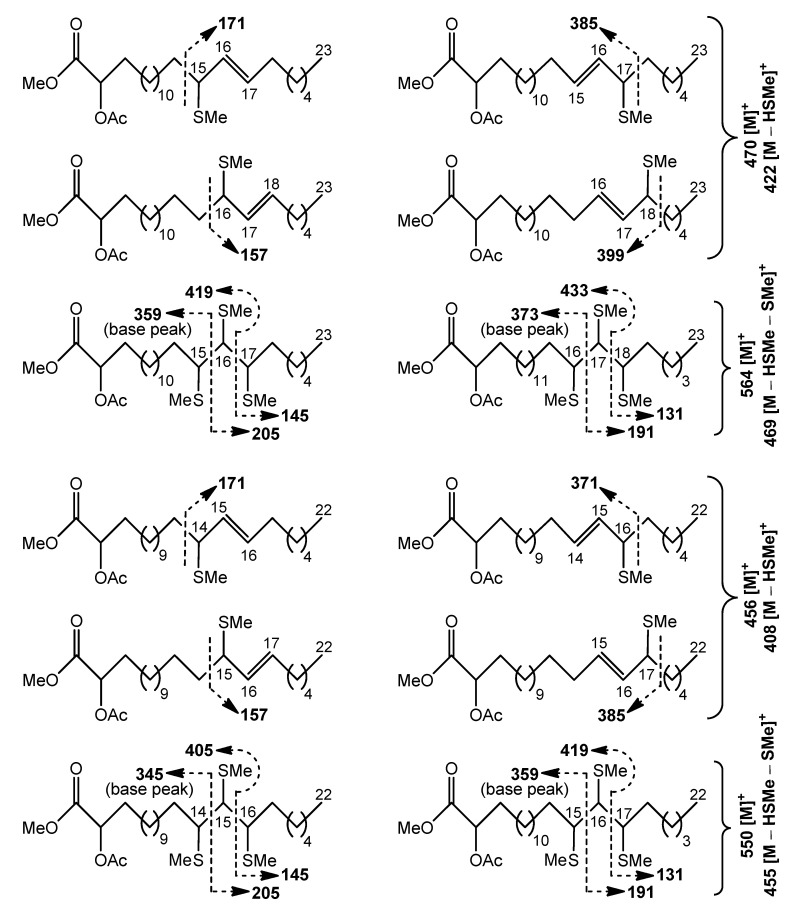
GC-MS cleavage patterns for the methyl esters of 2-acetyloxy С_23_ and С_22_ acids, containing an allylic methylthio group or three methylthio groups.

The oxidized cerebrosides from part 2 were acetylated to increase solubility of these relatively polar compounds in DMDS and other low-polarity or non-polar organic solvents. In this process, allylic hydroperoxides were transformed into enones (Scheme 3), as reported by Porter and Wujek [23]. The mixture obtained after acetylation was treated with NaBH_4_/CeCl_3_ [28] to convert enones into allylic alcohols. The resulting derivatives, containing an allylic hydroxy or acetyloxy group, were treated with DMDS, and the products of this reaction were hydrolyzed in MeCN/HCl. Liberated 2-hydroxy FAs were acetylated, methylated, and analyzed by a GC-MS method that revealed major mono(methylthio) and minor tris(methylthio) derivatives (Scheme 3 and Scheme 4). The mono(methylthio) compounds, containing an allylic methylthio group, were characterized by ^1^H-NMR resonances (CDCl_3_) of two *trans*-olefinic CH (*δ*_Н_ 5.175 dd, *J* = 9.0, 15.2 Hz, and 5.40 dt, *J* = 6.9, 15.2 Hz) and one CH (*δ*_Н_ 2.99 m), bearing a –SMe group (*δ*_H_ 1.97 s). The presence of the mid-chain allylic substructure was confirmed by a TOCSY (total correlation spectroscopy) experiment.

Cleavage patterns for methyl esters of 2-acetyloxy С_23_ (470 [M]^+^) and С_22_ (456 [M]^+^) acids, containing an allylic methylthio group, are depicted in Scheme 4. The positional isomers of these allylic thioethers were only partially GC-separated. The mass spectra of isomeric allylic thioethers (Figures S21–S28) were characterized by diagnostic peaks, corresponding to ions produced by α-cleavage. The fragments, formed by cleavage α to the carbon carrying an allylic methylthio group on the side remote from the carboxyl group included *m*/*z* 385 and 399 ions for the methyl esters of 2-acetyloxy C_23_ acids with –SMe group in the (*n*–7) and (*n*–6) positions, respectively (Figures S22 and S24). The peaks at *m*/*z* 371 and 385, observed in the mass spectra of methyl esters of homologous 2-acetyloxy C_22_ acids, represented fragments of the same origin (Figures S26 and S28). α-fragmentation of other mono(methylthio) derivatives gave rise to the ions at *m*/*z* 171 and 157, containing the methyl end of isomers with an allylic –SMe group in the (*n*–9) and (*n*–8) positions, respectively (Figures S21, S23, S25, and S27). Relative abundances of α-fragments in average mass spectrum were used to quantify isomer distribution, and approximately equal amounts of the four isomeric allylic thioethers were found. This finding may reflect the fact that these *S*-methyl derivatives were possibly products of allylic rearrangements, occurring prior to GC-MS analysis.

The minor tris(methylthio) derivatives of the methyl esters of 2-acetyloxy C_23_ (564 [M]^+^) or C_22_ (550 [M]^+^) acids produced more diagnostic fragments than the previously mentioned major mono(methylthio) derivatives. Expectedly, the cleavages of minor *S*-methylated compounds occurred between methylthio-carrying carbons to yield substantial fragment ions, as illustrated in Scheme 4 and Figures S29–S32. A cluster of four GC peaks for isomeric tris(methylthio) derivatives was observed. According to fragmentation patterns, there were two peaks representing positional isomers and two peaks that represented stereoisomers of these regioisomers on the chromatogram.

**Scheme A1:**
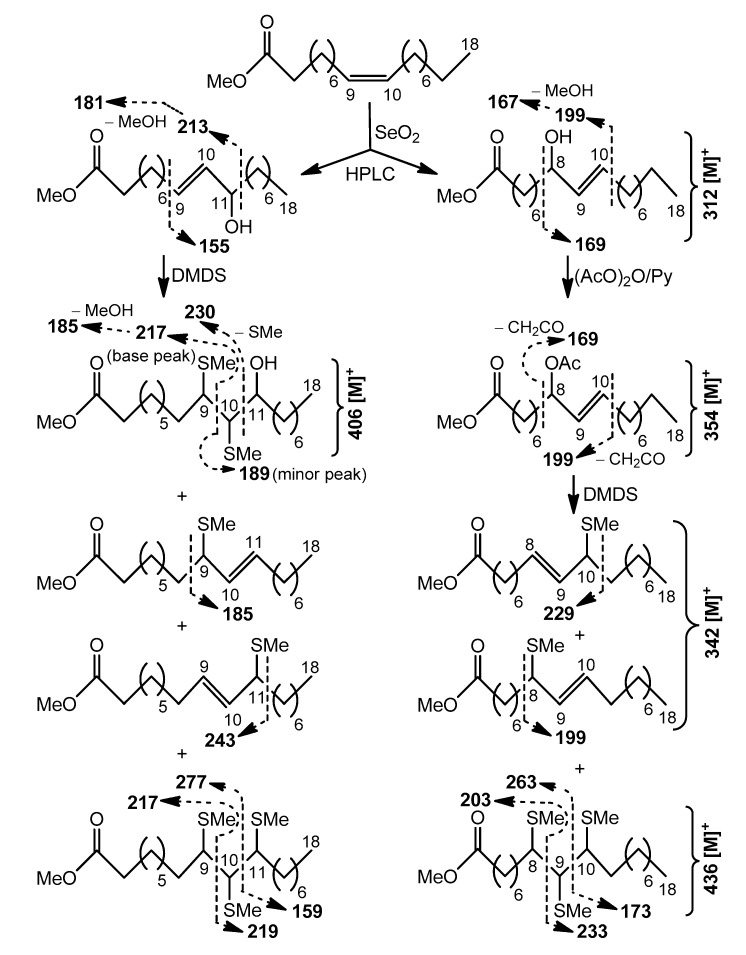
Allylic mono-hydroxylation of methyl oleate, followed by transformations of allylic alcohols into *S*-methyl derivatives. GC-MS cleavage patterns for the derivatives, obtained from methyl oleate, are depicted.

The results of the transformations of allylic alcohols and their acetates into methylthio derivatives (Scheme 3) were confirmed by experiments with model compounds, methyl esters of 11-hydroxy and 8-acetyloxy elaidic acids (prepared from methyl oleate, Appendix C, Scheme A1, Figures S33–S37). Under the conditions used here, the allylic alcohol acetates reacted with DMDS to give major allylic thioethers and minor tris(methylthio) derivatives. Allylic alcohols reacted with DMDS to give major DMDS adducts and minor allylic thioethers and tris(methylthio) derivatives. However, in contrast to the DMDS adducts of monoenes with an isolated double bond, the bis(methilthio) derivatives of allylic alcohols were destroyed during MeCN/HCl hydrolysis.

Previously, the synthesis of allylic thioethers from allylic alcohols and thiols was reported by Zhang et al. ([29]: iodine-catalyzed process) and Tabarelli et al. ([30]: catalyst-free approach). Regio-isomeric mixtures of allylic thioethers were produced when the allylic alcohol contained two different substituents. To explain the presence of regio-isomer products of 1,3-isomerization, the allylic cation, formed by water loss from the allylic alcohol, was proposed to be an intermediate in the reaction pathway [30]. The existence of a similar mechanism explains the formation of regio-isomeric allylic thioethers and their tris(methilthio) derivatives in the iodine-catalyzed reaction of allylic alcohols and their acetates with DMDS reported here. Additionally, allylic thioethers, which could undergo 1,3-isomerization under acidic conditions, were possibly formed from the bis-DMDS adducts of allylic alcohols during MeCN/HCl hydrolysis. As a result of these rearrangements, the FA methylthio derivatives, obtained from oxidized cerebrosides, gave characteristic mass spectra, permitting locations of three-carbon allylically oxygenated substructures, rather than double bonds in the starting acyl chains.

We clarified the acyl structures (Figure 1) using a logical approach. The GC-MS analyses of the methyl esters of 2-acetyloxy methylthio FAs (Scheme 4) revealed that the three-carbon allylically functionalized substructures of oxidized cerebrosides included С-15^/^–С-16^/^–С-17^/^ and С-16^/^–С-17^/^–С-18^/^ fragments for 2-hydroxy С_23_ acyl chains and С-14^/^–С-15^/^–С-16^/^ and С-15^/^–С-16^/^–С-17^/^ fragments for 2-hydroxy С_22_ acyl chains. According to the GC-MS analyses of the hydrogenated FA derivatives, the amide-linked FAs of oxidized cerebrosides contained hydroperoxy, hydroxy, or keto groups in the (*n*–8) or (*n*–7) positions, more specifically in the 16^/^ and 17^/^ positions of 2-hydroxy С_23_ acyl chains and the 15^/^ and 16^/^ positions of 2-hydroxy С_22_ acyl chains. A priori, an allylic hydroperoxy, hydroxy, or keto group should be located in the terminal points of the previously mentioned three-carbon substructures. Consequently, the С-15^/^–С-16^/^–С-17^/^ fragments of the С_23_ acyl chains (**a**–**a^//^**) contained such groups in position С-17^/^ and the double bond between С-15^/^ and C-16^/^, while the С-16^/^–С-17^/^–С-18^/^ fragments of the other C_23_ acyl chains (**b**–**b^//^**) had 16^/^-hydroperoxy/hydroxy/oxo groups and 17^/^,18^/^-double bonds. Similarly, the С-14^/^–С-15^/^–С-16^/^ fragments of the С_22_ acyl chains (**c**–**c^//^**) contained 16^/^-hydroperoxy/hydroxy/oxo groups and 14^/^,15^/^-double bonds, while the С-15^/^–С-16^/^–С-17^/^ fragments of the other C_22_ acyl chains (**d**–**d^//^**) had 15^/^-hydroperoxy/hydroxy/oxo groups and double bonds between C-16^/^ and C-17^/^.

The mono(methylthio) and tris(methylthio) derivatives of allylically oxygenated FAs were also used to explain other structural peculiarities of the starting material. Upon hydrogenation, all the methylthio derivatives lost *S*-methyl groups, giving saturated hydrocarbon chains. In particular, the transformations, shown in Scheme 3, with subsequent hydrodesulfurization allowed us to convert –CH=CH–CH(OOH/OH)– and –CH=CH–CO– substructures to –СН_2_–СН_2_–СН_2_– chains without reducing other oxygen-containing groups in the molecules. As a result, the unbranched structures of the parent amide-linked FAs were clarified using retention times of the 2-acetyloxy tricosanoic and docosanoic acid methyl esters, obtained from the methylthio derivatives. Then, these methyl esters were converted into (2*S*)-oct-2-yl esters of 2-hydroxy acids. The resulting (2*S*)-oct-2-yl esters of 2-hydroxy tricosanoic and docosanoic acids coeluted in GC analyses with the reference (2*S*)-oct-2-yl ester of (2*R*)-2-hydroxy tricosanoic or docosanoic acids, respectively, indicating (2*R*)-configurations of 2-hydroxy acids liberated from oxidized cerebrosides.

Thus, we used two complementary approaches for determining oxidized acyl chain structures in glycosphingolipids. Analysis of the FA derivatives from part 1 indicated the allylic oxygen-containing group positions, but hydrogenation resulted in the loss of information regarding the positions of double bonds in the starting material (approach 1). In the analysis of part 2 (approach 2), the data on the locations of three-carbon allylically oxygenated substructures in the FA derivatives and the information on their straight-chain structures and (2*R*)-configurations were obtained. Although there was no direct information about the position of the double bond in the FA esters, the combined data of approaches 1 and 2 allowed for determination of the double bond and allylic hydroperoxy, hydroxy, or keto group locations in each acyl chain.

*Analyses of Sphingoid Bases and Sugar from Parts 1 and 2*. The sphingoid bases, liberated by hydrolysis of oxidized cerebrosides, were obtained as acetylated derivatives. The ^1^Н-NМR data (*δ*_Н_ values of CH_2_-1–CH-4; CDCl_3_) and optical rotation value ([α]^25^_D_ = + 27.9, CHCl_3_) of the hydrogenated sphingoid base acetates, isolated from the hydrolysate of part 1, indicated their (2*S*,3*S*,4*R*)-configuration [31]. The ^1^Н-NМR spectrum of these compounds also showed signals of the terminal methyl groups of dominant normal-chain (*δ*_Н_ 0.88, t, *J* = 6.9 Hz) and minor cyclopropane-containing (*δ*_Н_ 0.89, t, *J* = 6.9 Hz) constituents. Under our conditions of hydrogenation, ring opening was not the dominant process for the minor constituent, so the signals of *cis*-cyclopropane protons at *δ*_Н_ −0.33 (dt, *J* = 4.2, 5.5 Hz), 0.56 (ddd, *J* = 4.2, 8.3, 8.3 Hz), and 0.645 (m) [32] were observed. The hydrolysis of derivatized oxidized cerebrosides from part 2 with subsequent acetylation of products gave three sphingoid base derivatives. Two spingoid base derivatives were the DMDS adducts of acetylated isomeric monoenoic С_20_ compounds. The mass spectrum of the DMDS adduct of major acetylated C_20_ monoene exhibited significant peaks at *m*/*z* 432 [M − H_3_CSC_9_H_18_]^+^, 372 [M − H_3_CSC_9_H_18_ − AcOH]^+^ (base peak), and 173 [H_3_CSC_9_H_18_]^+^, indicating 11,12-double bond in backbone **1**. Key peaks in the mass spectrum of the DMDS adduct of isomeric minor acetylated C_20_ monoene, which have a longer retention time in GC-MS, were observed at *m*/*z* 460 [M − H_3_CSC_7_H_14_]^+^, 400 [M − H_3_CSC_7_H_14_ − AcOH]^+^ (base peak), and 145 [H_3_CSC_7_H_14_]^+^, indicating Δ13 unsaturation in backbone **2**. A third acetylated sphingoid base, derived from cyclopropane-containing backbone **3**, did not give a DMDS adduct. In the mass spectrum of this compound, an [M − AcOH]^+^ ion fragmented to give discernible peaks at *m*/*z* 380 [M − AcOH − (CH_2_)_6_СH_3_]^+^ (α cleavage to a cyclopropane ring, at the C-14–C-15 position) and 394 [M − AcOH − (CH_2_)_5_СH_3_]^+^ (β-cleavage, at the C-15–C-16 position) that was characteristic of the peracetate of the С_21_ sphingoid base containing a ring between C-13 and C-14.

**Figure 6:**
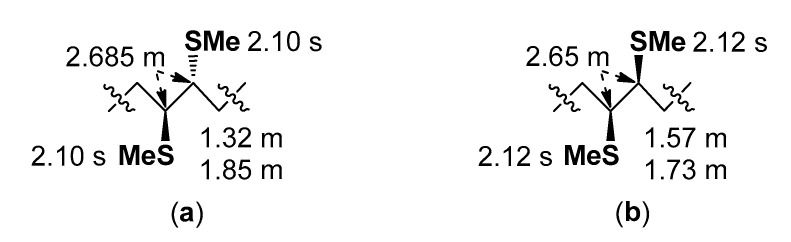
Some *δ*_H_ values (700 MHz, CDCl_3_) for the *S*-methylated substructures of (**a**) methyl threo-9,10-bis(methylthio)hexadecanoate and (**b**) methyl erythro-9,10-bis(methylthio)hexadecanoate. Unlike the signals of the methylthio groups of the threo-isomer, the signals of –SMe groups of the erythro-isomer were two partially resolved singlets at *δ*_H_ 2.121 and 2.119.

Methylthiolation of *cis*-monoenes and *trans*-monoenes with DMDS, as an anti-addition, leads to the threo-adducts and erythro-adducts, respectively [33,34]. The threo-diastereomers and erythro-diastereomers can be easily distinguished by NMR shifts of protons and carbons in and close to the 1,2-bis(alkylthio) moiety [35]. In addition to the data presented in the NMR study of Knothe and Steidley [35], we used *δ*_Н_ values of the DMDS adducts of two standards, methyl palmitoleate (Figure 6a) and its *trans*-isomer (Figure 6b), to confirm the configurations of double bonds in the monoenoic sphingoid base moieties of the starting oxidized cerebrosides. The *δ*_H_ values for CH_2_, α to the –CH(SMe)–CH(SMe)– moiety, were obtained through correlations in ^1^Н,^1^Н-COSY diagrams. The ^1^Н-NМR and ^1^Н,^1^Н-COSY spectra (CDCl_3_) of the DMDS derivatives of acetylated sphingoid bases, derived from backbones **1** and **2**, showed superimposed resonances of two vicinal CH groups (*δ*_Н_ 2.685, m), linked to –SMe (*δ*_H_ 2.10, s), and two α-CH_2_ groups (*δ*_Н_ 1.84, m; 1.32, m), characteristic of threo-diastereomers. Consequently, *cis*-monoenes (**1** and **2**) were precursors of these compounds.

GC analyses of peracetylated (2*R*)- and (2*S*)-oct-2-yl glucosides showed a D-configuration of glucose, released from parts 1 and 2 [36].

### 2.4. Oxidized Cerebrosides from the Extract of Aulosaccus sp.: Structures and Possible Origins

As a result of our study, structures of 18 previously unknown compounds, found in the complex mixture of the oxidized cerebrosides from the extract of *Aulosaccus* sp., were elucidated. These β-D-glucopyranosyl-(1→1)-ceramides (**1a**–**a^//^**, **1b**–**b^//^**, **2a**–**a^//^**, **2b**–**b^//^**, **3c**–**c^//^**, **3d**–**d^/^^/^**) were shown to contain phytosphingosine-type backbones, (2*S*,3*S*,4*R*,11*Z*)-2-aminoeicos-11-ene-1,3,4-triol (in **1**), (2*S*,3*S*,4*R*,13*Z*)-2-aminoeicos-13-ene-1,3,4-triol (in **2**), and (13S*,14R*)-2-amino-13,14-methylene-eicosane-1,3,4-triol (in **3**). These backbones were *N*-acylated with straight-chain monoenoic (2*R*)-2-hydroxy acids that had allylic hydroperoxy/hydroxy/keto groups on C-17**^/^** in the 15**^/^***E*-23:1 chain (**a**–**a^//^**), C-16**^/^** in the 17**^/^***E*-23:1 (**b**–**b^//^**) and 14**^/^***E*-22:1 (**c**–**c^//^**) chains, and C-15**^/^** in the 16**^/^***E*-22:1 chain (**d**–**d^//^**). Cerebrosides, having backbones **1**, **2**, and **3**, comprised, respectively, 60%, 20%, and 20% of the mixture. The percentages were calculated from the integration of signals of a cyclopropane ring in the ^1^H-NMR spectra of this mixture (backbone **3**) and from relative intensities of GC peaks, represented by DMDS derivatives of two acetylated isomeric monoenoic sphingoid bases (backbones **1** and **2**, Δ^11^:Δ^13^ ≈ 3:1). The GC-MS analysis of the hydrogenation products of amide-linked FAs indicated that the **a**:**b**, **c**:**d**, **a^/^**:**b^/^**, **c^/^**:**d^/^**, **a^//^**:**b^//^**, and **c^//^**:**d^//^** isomer ratios were approximately 1:1.Therefore, the employed complementary instrumental and chemical methods clarified structures of oxidized cerebrosides in a complex mixture, without requiring isolation or complete separation.

Previously, the possible precursors of the oxidized cerebrosides were found in the major RP-HPLC fraction of glycosphingolipids, isolated from the extract of *Aulosaccus* sp. These potential precursors were β-d-glucopyranosyl-(1→1)-ceramides that contained backbones **1** (60% in the fraction) and **2** (20%), *N*-acylated with (2*R*,16*Z*)-2-hydroxytricos-16-enoic acid, and backbone **3** (20%), *N*-acylated with (2*R*,15*Z*)-2-hydroxydocos-15-enoic acid [8]. Perhaps, peroxidation of the amide-linked FAs occurred symmetrically about the *cis*-(*n*–7) double bond, so a hydroperoxy, hydroxy, or keto group, found in the major *trans*-monoenoic peroxidation products, was located at each of the carbon atoms, which originally formed the double bond (namely, in the (*n*–8) and (*n*–7) positions, ≈1:1). In particular, structures **a**–**a^//^** and **b**–**b^//^** could be formed from С_23_Δ^16*Z*^ acyl chain while structures **c**–**c^//^** and **d**–**d^//^** could be formed from the С_22_Δ^15*Z*^ acyl chain.

According to the product composition, photo-oxidation and autooxidation [1] are possible mechanisms involved in the formation of the oxidized cerebrosides from the extract of *Aulosaccus* sp. However, we would like to point to another possible origin of the oxidized cerebrosides in *Aulosaccus* sp., taking into account the relationship between these oxidation products and other compounds isolated from the same sponge sample. In particular, some bacterial branched-chain, cyclopropane-containing FAs, and their monoenoic precursors were present in significant amounts in *Aulosaccus* sp. [32], and an overwhelming number of the sterols (stanols, Δ^5^-, Δ^7^-, and Δ^8(14)^-sterols) of this sponge were oxidized to the corresponding 3-ketosteroids [37]. The occurrence of these FAs and steroids in *Aulosaccus* sp. suggested this sponge was associated with actinobacteria, known as sponge-specific microorganisms [38] and sterol degraders [39]. Cholesterol oxidase, produced by a variety of actinobacteria [40], could catalyze the transformations of the previously mentioned sterols into 3-ketosteroids [41] with the generation of H_2_O_2_. We suggest that H_2_O_2_ production in the enzymatic oxidation of *Aulosaccus* sp. sterols led to oxidative transformations of a certain part of cerebrosides, located in the membranes of eukaryotic cells together with sterols.

## 3. Materials and Methods

### 3.1. General Procedures

^1^H-, ^13^C-NMR, ^1^Н,^1^Н-COSY, HSQC, HMBC, NOESY (Nuclear Overhauser Effect Spectroscopy), and TOCSY spectra (in CD_3_OD or CDCl_3_) were recorded on Bruker Avance III HD 500 and Bruker Avance III 700 spectrometers (Bruker BioSpin, Bremen, Germany) at 125 MHz (^13^C), 500 (^1^H), and 700 (^1^H) MHz. Chemical shifts (ppm) were internally referenced to the corresponding residual solvent signals *δ*_H_ 3.306/ *δ*_C_ 49.6 for CD_3_OD. For the spectra of compounds in CDCl_3_, TMS was used as an internal standard. ESI mass spectra were recorded on a Q-TOF 6510 mass spectrometer (Agilent Technologies, Santa Clara, CA, USA). GC-MS analyses were carried out on a Hewlett Packard HP6890 GC System (Hewlett-Packard Company, Palo Alto, CA, USA) with an HP-5MS (J&W Scientific, Folsom, CA, USA) capillary column (30.0 m × 0.25 mm), helium as the carrier gas, and 70 eV ionizing potential. GC analyses were done on an Agilent 6850 Series GC System chromatograph (Agilent Technologies, Santa Clara, CA, USA) equipped with an HP-1 (Agilent technology, Santa Clara, CA, USA) capillary column (30 m × 0.32 mm), the carrier gas was helium (flow rate 1.7 mL/min), and the detector temperature was 300 °C (or 280 °C for per-acetylated (2*R*)-oct-2-yl glucosides). The GC-MS analyses of FA esters and per-acetylated sphingoid bases were done using the injector temperature of 270 °C and the temperature program 100 °C (1 min)–10 °C/min–280 °C (30 min). The GC analyses of per-acetylated (2*R*)-oct-2-yl derivatives of D-Glc and L-Glc were carried out using the injector temperature of 150 °C and the temperature program 100 °C (0.5 min)–5 °C/min–250 °C (10 min). Optical rotation was measured on a Perkin–Elmer polarimeter, model 343 (Perkin-Elmer GmbH, Überlingen, Germany). Column chromatography was performed using silica gel (50/100 μm, Sorbpolimer, Krasnodar, Russia). RP-HPLC separations were performed using a Du Pont Series 8800 Instrument (DuPont, Wilmington, DE, USA) with an RIDK-102 refractometer (Laboratorni pristroje, Praha, Czechoslovakia). Two columns (Agilent Technologies, Santa Clara, CA, USA), Agilent ZORBAX Eclipse XDB-C8 (4 × 150 mm), and Agilent ZORBAX SB-C18 (4.6 × 250 mm), were used for the HPLC.

### 3.2. Animal Material

The sample of the genus *Aulosaccus* (phylum Porifera, class Hexactinellida, order Lyssacinosida, family Rossellidae), 930 g (wet weight), was collected in July 2011 by dredging (with small Sigsbi trail) from a 505-m depth near Iturup Island (Kuril Islands, 45°01.05^/^N, 147°00.03^/^E) during a cruise onboard the r/v “Academik Oparin”. The species was identified by Dr A.L. Drozdov (A.V. Zhirmunskii Institute of Marine Biology, Far Eastern Branch of RAS, Vladivostok, Russia). The collected sponge was stored at −15 °C.

### 3.3. Extraction and Isolation

The isolation of total cerebroside sum (238 mg) from the EtOH extract of *Aulosaccus* sp. was described in our previous article [8]. This sum was separated by RP-HPLC (Agilent ZORBAX Eclipse XDB-C8 column, EtOH-H_2_O, 85:15, *v*/*v*). A mixture of the most polar minor cerebrosides (22.4 mg) was re-chromatographed (EtOH-H_2_O-CHCl_3_, 75:25:5, *v*/*v*) to give a major fraction (4.4 mg) containing oxidized cerebrosides: (+)HR-ESI-MS: 910.6606 ([M + Na]^+^ of isomeric allylic hydroperoxides, C_49_H_93_NO_12_Na^+^; calc. 910.6590), 894.6649 ([M + Na]^+^ of isomeric allylic alcohols, C_49_H_93_NO_11_Na^+^; calc. 894.6641), and 892.6534 ([M + Na]^+^ of isomeric enones, C_49_H_91_NO_11_Na^+^; calc. 892.6484); (−)HR-ESI-MS: 922.6387 ([M + Cl]^−^ of isomeric allylic hydroperoxides, C_49_H_93_NO_12_Cl^−^; calc. 922.6386), 906.6425 ([M + Cl]^−^ of isomeric allylic alcohols, C_49_H_93_NO_11_Cl^−^; calc. 906.6437), and 904.6353 ([M + Cl]^−^ of isomeric enones, C_49_H_91_NO_11_Cl^−^; calc. 904.6281); ^1^H- and ^13^C-NMR (CD_3_OD): see Table 1 and Figures S2 and S3. This fraction was divided into two parts. The constituents of parts 1 and 2 were analyzed using different chemical procedures.

### 3.4. Analyses of FAs, Sphingoid Bases, and Sugar from Oxidized Cerebrosides (Parts 1 and 2)

The constituents of part 1 (3.0 mg) were hydrogenated in EtOH over Adams’ catalyst at room temperature (10 h). The resulting hydrogenation product was hydrolyzed in MeCN (0.45 mL) and 5 N HCl (0.05 mL) for 4 h at 75 °C in a capped vial. Hydrolysate was concentrated in vacuo, partitioned between CHCl_3_ (1.0 mL) and H_2_O (3 × 1.0 mL). The H_2_O extract was evaporated to dryness to give sugar-containing subfraction. The CHCl_3_ layer was concentrated in vacuo, and a dry residue was acetylated with Ac_2_O (0.2 mL) in pyridine (0.2 mL), overnight. The acetylated material was subjected to chromatography over silica gel column (3.0 cm × 1.2 cm), using hexane-ethylacetate (5:1→2:1→1:1, *v*/*v*) system, and then CHCl_3_-EtOH (1:1, *v*/*v*) system. Elution with 60 mL of hexane-ethylacetate (2:1, *v*/*v*) gave a subfraction of per-acetylated sphingoid bases (0.8 mg). 2-Acetyloxy FA (0.7 mg) was eluted with 20 mL of CHCl_3_-EtOH (1:1, *v*/*v*) system. The subfraction of the acetates of sphingoid bases was re-purified by column chromatography (SiO_2_, 3.5 cm × 1.2 cm) eluting with 60 mL of hexane-ethyl acetate (2:1, *v*/*v*). The eluate was evaporated in vacuo yielding a mixture (0.5 mg) of (2*S*,3*S*,4*R*)-2-aminoeicosane-1,3,4-triol (*n*-t20:0, using shorthand nomenclature [42]) and (2*S*,3*S*,4*R*,13S*,14R*)-2-amino-13,14-methylene-eicosane-1,3,4-triol (*cis*-13,14-methylene-t21:0): [α]^25^_D_ = +27.9 (*c* = 0.03, CHCl_3_), ^1^H-NMR (500 MHz, CDCl_3_): 5.94 (d, *J* = 9.4 Hz, NH), 5.10 (dd, *J* = 3.0, 8.1 Hz, H-3), 4.94 (dt, *J* = 3.0, 9.9 Hz, H-4), 4.475 (m, H-2), 4.29 (dd, *J* = 4.8, 11.6 Hz, H-1b), 4.01 (dd, *J* = 3.0, 11.6 Hz, H-1a), 2.08 (s, −OCOCH_3_ at C-3), 2.05 (two s, −OCOCH_3_ at C-4 and at C-1), 2.025 (s, −NHCOC**H_3_**), 1.64 (m, H_2_C-5), 1.45−1.05 (m, –(CH_2_)_n_–), 0.88 (t, *J* = 6.9 Hz, H_3_C-20 of *n*-t20:0), 0.89 (t, *J* = 6.9 Hz, H_3_C-20 of *cis*-13,14-methylene-t21:0), 0.65 (m, H-13, H-14 of *cis*-13,14-methylene-t21:0), 0.565 (ddd, *J* = 4.1, 8.3, 8.3 Hz, H-21b of *cis*-13,14-methylene-t21:0), −0.33 (dt, *J* = 4.1, 5.5 Hz, H-21a of *cis*-13,14-methylene-t21:0); (2*S*,3*S*,4*R*)-2-aminoeicosane-1,3,4-triol MS, *m*/*z* (relative intensity, %): 452/453/454 [M − AcOH − H/M − AcOH/M − AcNH_2_]^+^ (1.0/0.3/0.9), 380 [M − AcOCH_2_ − AcOH]^+^ (15), 338 [M − AcOCH_2_ − AcOH − CH_2_CO]^+^ (14), 333 [M − 3AcOH]^+^ (19), 320 [M − AcOCH_2_ − 2AcOH]^+^ (23),144 [AcOCH_2_CH(NHAc)]^+^ (90), 102 [AcOCH_2_CH(NHAc) − СН_2_СO]^+^ (45), 84 [AcOCH_2_CH(NHAc) − AсOН]^+^ (100); (13S*,14R*)-2-amino-13,14-methylene-eicosane-1,3,4-triol MS, *m*/*z* (relative intensity, %): 525 [M]^+^ (2), 465 [M − AcOH]^+^ (21), 406 [M − AcOH − AcNH_2_]^+^ (9), 394 (6), 380 (5), 332 [M − AcOCH_2_ − 2AcOH]^+^ (12), 144 [AcO-CH_2_CH(NHAc)]^+^ (52), 102 [AcOCH_2_CH(NHAc) − СН_2_СO]^+^ (52), 84 [AcOCH_2_CH(NHAc) − AсOН]^+^ (100). The components of the subfraction of 2-acetyloxy FAs were methylated by a standard method with *N*-nitroso-*N*-methylurea. The resulting compounds were purified by column chromatography (SiO_2_, 3.5 cm × 1.2 cm) eluting with 44 mL of hexane-ethyl acetate (10:1, *v*/*v*). The elution gave a mixture of the methyl esters of 2-acetyloxy FAs (0.5 mg): ^1^H-NMR (CDCl_3_, 700 MHz): 4.99 (dd, *J* = 6.6, 6.9 Hz, H-2), 4.855 (m, mid-chain –С**Н**(OAс)– of diacetyloxy acids), 3.74 (s, H_3_CO– at C-1), 2.38 (t, *J* = 7.4 Hz, mid-chain *–***H**_2_C–CO–C**H**_2_– of keto acids), 2.135 (s, −OCOCH_3_ at C-2), 1.82 (m, H_2_C-3), 1.45–1.15 (m, –(CH_2_)_n_–), 0.88 (a series of triplets about this point, *J* ≈ 7.0 Hz), the methyl esters of keto C_23_ and C_22_ acids MS: Figures S4–S7, S12–S16; the methyl esters of diacetyloxy C_23_ and C_22_ acids MS: Figures S8–S11, S17–S20, the methyl ester of saturated 2-acetyloxy C_23_ acid MS: 426 [M]^+^ (0.04%), 384 [M − CH_2_CO]^+^ (100%), 352 [M − CH_2_CO − MeOH]^+^ (34%), and the methyl ester of saturated 2-acetyloxy C_22_ acid MS: 412 [M]^+^ (0.04%), 370 [M − CH_2_CO]^+^ (100%), 338 [M − CH_2_CO − MeOH]^+^ (35%).

Components of part 2 (1.4 mg) were acetylated with Ac_2_O (0.2 mL) in pyridine (0.2 mL), overnight. After vacuum drying, CHCl_3_ (two drops) and CeCl_3_·7H_2_O (18.6 mg) were added to the acetylated material, and the mixture was dissolved in MeOH (0.125 mL). NaBH_4_ (5 mg, excess) was slowly added with stirring. After 5 min, the reaction was quenched by the addition of NH_4_Cl water solution (5.4 mg in 0.2 mL H_2_O). A dry residue was obtained after complete evaporation of the reaction mixture. It was extracted with CHCl_3_ to remove most inorganic admixtures. After vacuum drying of the CHCl_3_ extract, the resulting dry material was dissolved in DMDS (0.1 mL), and a solution (0.025 mL) of iodine in diethyl ether (60 mg/mL) was added. The mixture was kept for 4 days at room temperature. Then the reaction was stopped by adding aqueous solution of Na_2_S_2_O_3_ (5%, 0.2 mL), and the mixture was extracted with hexane (5 × 0.5 mL). The hexane extract was evaporated to dryness, and the resulting compounds were hydrolyzed in MeCN/HCl. Then, subfractions, containing sugar, peracetylated sphingoid bases (0.4 mg), and 2-acetyloxy FAs (0.3 mg), were obtained, as described above. The subfraction of peracetylated sphingoid bases consisted of peracetylated (13S*,14R*)-2-amino-13,14-methylene-eicosane-1,3,4-triol and DMDS derivatives of peracetylated (11*Z*)-2-aminoeicos-11-ene-1,3,4-triol and (13*Z*)-2-aminoeicos-13-ene-1,3,4-triol: ^1^H-NMR (500 MHz, CDCl_3_): 5.93 (d, *J* = 9.5 Hz, NH), 5.095 (dd, *J* = 3.1, 8.3 Hz, H-3), 4.94 (dt, *J* = 3.1, 9.9 Hz, H-4), 4.47 (m, H-2), 4.29 (dd, *J* = 4.8, 11.8 Hz, H-1b), 4.00 (dd, *J* = 3.1, 11.8 Hz, H-1a), 2.685 (m, –C**H**(SCH_3_)–C**H**(SCH_3_)–), 2.10 (s, –CH(SC**H_3_**)–CH(SC**H_3_**)–), 2.08 (s, −OCOCH_3_ at C-3), 2.05 (two s, −OCOCH_3_ at C-4 and at C-1), 2.025 (s, −NHCOC**H_3_**), 1.84 (m; H-b, –**H**_2_CCH(SCH_3_)–CH(SCH_3_)C**H**_2_–), 1.64 (m, H_2_C-5), 1.42−1.18 (m, –(CH_2_)_n_–), 1.32 (m, H-a, –**H**_2_CCH(SCH_3_)–CH(SCH_3_)C**H**_2_–), 0.88 (t, *J* = 6.9 Hz, H_3_C-20 of *n*-t20:0), 0.89 (t, *J* = 6.9 Hz, H_3_C-20 of *cis*-13,14-methylene-t21:0), 0.645 (m, H-13, H-14 of *cis*-13,14-methylene-t21:0), 0.56 (ddd, *J* = 4.2, 8.2, 8.2 Hz, H-21b of *cis*-13,14-methylene-t21:0), −0.33 (dt, *J* = 4.2, 5.4 Hz, H-21a of *cis*-13,14-methylene-t21:0). The DMDS derivative of per-acetylated (11*Z*)-2-aminoeicos-11-ene-1,3,4-triol MS, *m/z* (relative intensity, %): 557 [M − HSMe]^+^ (1.4), 432 [M − H_3_CSC_9_H_18_]^+^ (24), 372 [M − H_3_CSC_9_H_18_ − CH_3_COOH]^+^ (100), 330 (11), 312 (6), 173 [H_3_CSC_9_H_18_]^+^ (30), 102 [AcOCH_2_CH(NHAc) − СН_2_СO]^+^ (25), 84 [AcOCH_2_CH(NHAc) − AсOН]^+^ (45). The DMDS derivative of per-acetylated (13*Z*)-2-aminoeicos-13-ene-1,3,4-triol MS, *m*/*z* (relative intensity, %): 557 [M − HSMe]^+^ (2), 460 [M − H_3_CSC_7_H_14_]^+^ (24), 400 [M − H_3_CSC_7_H_14_ − CH_3_COOH]^+^ (100), 358 (10), 340 (11), 145 [H_3_CSC_7_H_14_]^+^ (36), 102 [AcOCH_2_CH(NHAc) − СН_2_СO]^+^ (36), 84 [AcOCH_2_CH(NHAc) − AсOН]^+^ (55). The 2-acetyloxy acids from FA subfraction were methylated using *N*-nitroso-*N*-methylurea. The resulting methyl esters were purified by column chromatography (SiO_2_, 3.5 cm × 1.2 cm), eluting with 44 mL of hexane-ethyl acetate (10:1, *v*/*v*). The elution gave a mixture, containing the methyl esters of major mono(methylthio) and minor tris(methylthio) derivatives of 2-acetyloxy FAs (0.2 mg): ^1^H-NMR (CDCl_3_, 700 MHz): 5.40 (dt, *J* = 6.9, 15.2 Hz, –**H**C=CH–CH(SCH_3_)–), 5.175 (dd, *J* = 9.0, 15.2 Hz, –HC=C**H**–CH(SCH_3_)–), 4.99 (dd, *J* = 6.2, 6.9 Hz, H-2), 3.74 (s, H_3_CO– at C-1), 2.99 (m, –HC=CH–C**H**(SCH_3_)–), 2.135 (s, −OCOCH_3_ at C-2), 2.04 (m, allylic CH_2_), 1.965 (s, –HC=CH–CH(SC**H_3_**)–), 1.82 (m, H_2_C-3), 1.45–1.15 (m, –(CH_2_)_n_–), 0.88–0.89 (a series of triplets, *J* ≈ 7.0 Hz), the methyl esters of the mono(methylthio) derivatives of 2-acetyloxy C_23_ acid MS: Figures S21–S24, the methyl esters of the mono(methylthio) derivatives of 2-acetyloxy C_22_ acid MS: Figures S25–S28, the methyl esters of the tris(methylthio) derivatives of 2-acetyloxy C_23_ acid MS: Figures S29 and S30, the methyl esters of the tris(methylthio) derivatives of 2-acetyloxy C_22_ acid MS: Figures S31 and S32. For the methyl esters of 2-acetyloxy C_23_ acids containing an allylic –SMe group, the order of elution was 15-methylthio-16-ene→17-methylthio-15-ene→16-methylthio-17-ene→18-methylthio-16-ene. The elution order for the methyl esters of 2-acetyloxy C_22_ acids containing an allylic –SMe group was 14-methylthio-15-ene→16-methylthio-14-ene→15-methylthio-16-ene→17-methylthio-15-ene. The mixture of these methylthio derivatives was hydrogenated over Adams’ catalyst in AcOH at room temperature (12 h). The hydrogenation gave methyl 2-(acetyloxy)tricosanoate and methyl 2-(acetyloxy)docosanoate. The straight-chain structures of these compounds were clarified, using the GC-MS data on the methyl esters of straight-chain 2-acetyloxy tricosanoic and docosanoic acids, released from the non-oxidized cerebrosides of *Aulosaccus* sp. Then, methyl 2-(acetyloxy)tricosanoate and methyl 2-(acetyloxy)docosanoate, obtained by hydrodesulfurization, were de-esterified in MeCN (0.45 mL) and 5N HCl (0.05 mL) for 4 h at 73−75 °C to give free FAs. These FAs were converted into (2*S*)-oct-2-yl esters of 2-hydroxy FAs by treatment with 2% H_2_SO_4_ in (2*S*)-octan-2-ol (0.2 mL) for 4 h at 75 °C in a capped vial [8]. In GC analyses, the retention times of the resulting (2*S*)-oct-2-yl ester of 2-hydroxy tricosanoic and docosanoic acids were identical with those of the standard (2*S*)-oct-2-yl esters of (2*R*)-2-hydroxy tricosanoic and docosanoic acids, respectively.

The absolute configuration of glucose, released from oxidized cerebrosides, was determined by the GC analyses of per-acetylated (2*R*)-oct-2-yl glycosides according to the method of Leontein et al. [41]. The sugar (1.2 mg), (2*R*)-octan-2-ol (0.4 mL), and one drop of trifluoroacetic acid in a capped vial were kept for 7 h at 120 °C with stirring. Then, the mixture was concentrated in vacuo and acetylated with Ac_2_O (0.4 mL) in pyridine (0.4 mL), overnight. The acetylated material was purified by column chromatography (SiO_2_, 3.0 cm × 1.2 cm), eluting with a mixture of hexane/ethylacetate (5:1, *v*/*v*). The eluate was evaporated, yielding per-acetylated (2*R*)-oct-2-yl glucoside. Standards, D-Glc (1.0 mg) and L-Glc (1.0 mg), were treated and derivatized under the same conditions that were applied to the sugar subfractions, liberated from parts 1 and 2. The GC profiles (the retention times and intensities of GC peaks) of the derivatives of D-Glc and sugar, obtained from cerebrosides, were proven to be identical.

## Figures and Tables

**Table 1:** ^1^Н-(500 MHz) and ^13^С-(125 MHz) NMR data (CD_3_OD) ^1^ for the RP-HPLC fraction, containing compounds **1a**–**a^//^**, **1b**–**b^//^**, **2a**–**a^//^**, **2b**–**b^//^**, **3c**–**c^//^**, and **3d**–**d^//^**.

Position	*δ*_H_ (mult., *J* Hz)	*δ*_C_
Sphingoid base moieties		
1	3.80 (dd, 3.9, 10.5), 4.045 (dd, 6.2, 10.5)	70.6
2	4.25 (m)	52.3
3	3.60 (t, 6.0)	76.1
4	3.51 (m)	73.6
5	1.39 (m), 1.64 (m)	33.7
6	1.31 (m), 1.55 (m)	27.6
СН_2_-pool	1.22−1.42 (m)	30.6–32.0
Allylic СН_2_ of **1** and **2**	2.02 (m)	28.7–28.85
Olefinic СН of **1** and **2**	5.34 (m)	131.4–131.5
12, 15 of **3**	1.17 (m), 1.37 (m)	30.5
13, 14 of **3**	0.67 (m)	17.5
21 of **3**	–0.33 (dt, 4.1, 5.3), 0.58 (ddd, 4.1, 8.3, 8.3)	12.2
18	1.28 (m)	33.5
19	1.31 (m)	24.3
20 of **1** and **2** 20 of **3**	0.895 (t, 6.9) 0.899 (t, 7.0) ^2^	15.0 15.0
*N*-Acyl moieties (some general features ^3^)		
1^/^	−	177.7
2^/^	4.01 (dd, 4.0, 7.7)	73.6
3^/^	1.60 (m), 1.74 (m)	36.4
4^/^	1.42 (m)	26.7
СН_2_-pool	1.22−1.42 (m)	30.6–32.0
(*n*−2) CH_2_	1.28 (m)	33.7
(*n*−1) CH_2_	1.32 (m)	24.2
CH_3_	0.893 and 0.897 (t, 7.0) ^2^	15.05
β-Glucopyranoside moiety		
1^//^	4.28 (d, 7.8)	105.3
2^//^	3.17 (dd, 7.8, 9.1)	75.6
3^//^	3.35 (m)	78.5
4^//^	3.27 (m)	72.2
5^//^	3.27 (m)	78.6
6^//^	3.66 (m), 3.865 (dd, 1.1, 11.9)	63.3

^1^ All signals were assigned based on data of ^1^Н−^1^Н COSY, HSQC, and НМВС experiments. ^2^ The two resolved triplets were observed in 700-MHz spectra. ^3^ *δ*_H_ and *δ*_C_ values for acyl fragments with allylic –OOН/–OН/=O are given separately (Figure 5).

## Supplementary Materials

The following are available online. Figure S1a: Base peak chromatogram from UPLC-MS analysis of oxidized cerebrosides (positive ion mode). Figure S1b: Extracted-ion chromatograms from UPLC-MS analysis of oxidized cerebrosides. Scheme S1: Hock fragmentation of some isomeric allylic hydroperoxides found in the present study. Figures S2, S2a–S2c: ^1^Н-NMR spectra (CD_3_OD, 500 MHz) of the RP-HPLC fraction, containing oxidized cerebrosides. Figure S3 ^13^С-NMR spectrum (CD_3_OD, 125 MHz) of the RP-HPLC fraction containing oxidized cerebrosides. Figures S4–S7: Mass spectra of samples, enriched in the methyl esters of 2-acetyloxy C_23_ and C_22_ acids with a keto group in the (*n*–8) or (*n*–7) positions. Figures S8–S11: Mass spectra of samples, enriched in the methyl esters of 2-acetyloxy C_23_ and C_22_ acids with an additional acetyloxy group in the (*n*–8) or (*n*–7) positions. Figures S12, S13: Mass spectra of samples, enriched in the methyl esters of 2-acetyloxy C_23_ acids with a keto group in the (*n*–9) or (*n*–6) positions. Figure S14: Averaged mass spectrum for the four methyl ester of isomeric 2-acetyloxy keto C_23_ acids. Figures S15, S16: Mass spectra of samples, enriched in the methyl esters of 2-acetyloxy C_22_ acids with a keto group in the (*n*–9) or (*n*–6) positions. Figures S17–S20: Mass spectra of samples, enriched in the methyl esters of 2-acetyloxy C_23_ and C_22_ acids with an additional acetyloxy group in the (*n*–9) or (*n*–6) positions. Figures S21–S28: Mass spectra of samples, enriched in the methyl esters of 2-acetyloxy C_23_ and C_22_ acids with an allylic methylthio group. Figures S29–S32: Mass spectra of the methyl esters of 2-acetyloxy tris(methylthio) C_23_ and C_22_ acids. Figure S33: Mass spectrum of the bis(methylthio) adduct of methyl 11-hydroxy elaidate. Figure S34: Superimposed mass spectra of overlapping methyl 9-(methylthio)octadec-10-enoate and 11-(methylthio)octadec-9-enoate. Figure S35: Mass spectrum of methyl 9,10,11-tris(methylthio)octadecanoate. Figure S36: Superimposed mass spectra of overlapping methyl 10-(methylthio)octadec-8-enoate and 8-(methylthio)octadec-9-enoate. Figure S37: Mass spectrum of methyl 8,9,10-tris(methylthio)octadecanoate. A section before Figures S33–S37 provide experimental details, concerning allylic monohydroxylation of methyl oleate and methylthiolation of allylic hydroxy/acetyloxy elaidates.

## Appendix A

The differences in *δ*_C_ (CD_3_OD, Figure 5a,b and Figure A1a,b) of allylic –Н**С**(OOH)– (Δ*δ*_C_ = *δ*_C_ *_trans_* − *δ*_C_ *_cis_* = 88.2 − 82.5 = 5.7) and –Н**С**(OH)– (Δ*δ*_C_ = *δ*_C_ *_trans_* − *δ*_C_ *_cis_* = 74.4 − 68.7 = 5.7) were close to the corresponding values (CDCl_3_) of *trans*-monoenoic and *cis*-monoenoic allylic hydroperoxides (Δ*δ*_C_ = 86.9 − 81.1 = 5.8) and allylic alcohols (Δ*δ*_C_ = 73.1 − 67.5 = 5.6), calculated in accordance with data in the literature [43]. Additionally, the signals of –**Н**С(OH)– of *trans*-monoenoic allylic alcohols (*δ*_Н_ 3.94, Figure 5b) were shifted upfield (by 0.42 ppm) compared to their *cis*-isomers (*δ*_Н_ 4.36, Figure A1b), as described for *cis*/*trans*-isomeric methyl 12-hydroxyoctadec-10-enoate and 9-hydroxyoctadec-10-enoate in CDCl_3_ ([44]: Δ*δ*_H_ = *δ*_H_ *_cis_* − *δ*_H_ *_trans_* = 4.4 − 4.0 = 0.4). Similarly, the signals of –**Н**С(OOH)– of *trans*-monoenoic hydroperoxides (*δ*_Н_ 4.16, Figure 5a) were shifted upfield (by 0.46 ppm) compared to their *cis*-isomers (*δ*_Н_ 4.62, Figure A1a), as observed earlier ([45]: Δ*δ*_H_ = *δ*_H_ *_cis_* − *δ*_H_ *_trans_* = 4.68 − 4.22 = 0.46 for *cis*/*trans*-isomeric hydroperoxides of methyl oleate in CDCl_3_).

## Appendix B

Although a single peak in the GC chromatogram represented overlapping esters of four isomeric keto acids, the mass spectra, obtained from different points in the GC peak, exhibited fragmentations of samples, enriched in the isomers with keto groups in the (*n*–9)→(*n*–8)→(*n*–7)→(*n*–6) positions (in this order of elution). Mass spectra of methyl esters of minor keto C_23_ and C_22_ acids displayed homologous pairs of *m*/*z* 141/156 and 99/114 fragments, containing methyl ends of the molecules with keto groups on the (*n*–9) or (*n*–6) carbon, respectively (Figures S12, S13, S15, and S16). The total mass spectra, obtained by averaging the spectra over the selected GC peaks, showed significant differences between relative abundances of several ions, characteristic of different isomers. For the overlapping esters of 15-keto, 16-keto, 17-keto, and 18-keto C_23_ acids, the abundance ratio of the characteristic ions of *m*/*z* 342, 356, 370, and 384 were about 1:3:3:1 (Figure S14). Accordingly, the mixture of C_23_ acid derivatives contained significant amounts of isomers with a keto group in the (*n*–8) and (*n*–7) positions and minor amounts of isomers with a keto group located at the (*n*–9) and (*n*–6) positions. The overlapping methyl esters of homologous keto C_22_ acids exhibited similar fragmentation and elution behavior.

GC-MS analysis also revealed the presence of clusters of closely overlapping chromatographic peaks for derivatives of regio-isomeric diacetyloxy C_23_ and C_22_ acids, including minor components. In particular, the α-fragments included the ions of *m*/*z* 371 and 413 (Figures S17 and S18) for the derivatives of 2,15-diacetyloxy and 2,18-diacetyloxy C_23_ acids, respectively, and the ions of *m*/*z* 357 and 399 (Figures S19 and S20) for the 2,14-diacetyloxy and 2,17-diacetyloxy C_22_ acid derivatives, respectively. The abundant ions of *m*/*z* 269 and 311 (Figures S17 and S18), which were detected in the mass spectra of the methyl esters of isomeric minor 2-acetyloxy C_23_ acids, confirmed the presence of second acetyloxy groups on C-15 and C-18, respectively. Similarly, *m*/*z* 255 and 297 ions in the mass spectra of the methyl esters of minor 2-acetyloxy C_22_ acids (Figures S19 and S20) indicated a second acetoxy group on C-14 and C-17, respectively. α-Fragmentation of isomers with an acetyloxy group in the (*n*–9), (*n*–8), (*n*–7), or (*n*–6) positions (in this order of elution) and subsequent loss of AcOH gave rise to *m*/*z* 125, 111, 97, and 83 ions, respectively, containing the methyl end of acyl chains. Among these α-fragments, the largest fragment of *m*/*z* 125 was much less abundant than the related ions. Unfortunately, the percentages of all the methyl esters of isomeric diacetylated FAs, which were only partially separated by GC, could not be accurately evaluated on the basis of mass spectral data because the presence of diagnostic α-fragments, formed from isomers with an acetyloxy group in the (*n*–9) positions, were masked by other peaks in averaged mass spectrum. However, for diacetyloxy C_23_ acid derivatives with an isolated acetyloxy group in the (*n*–8), (*n*–7), or (*n*–6) positions, the abundance ratio of the diagnostic α-fragments of *m*/*z* 385, 399, and 413, respectively, were about 3:3:1. The abundance ratio of homologous α-fragments (*m*/z 371, 385, and 399) were nearly the same for the derivatives of diacetyloxy C_22_ acids. Accordingly, the spectra, recorded at the top of the most intense overlapping GC peaks, exhibited fragmentation patterns of major isomers with an acetyloxy group in the (*n*–8) or (*n*–7) positions, while the mass spectra, recorded at lower points, belonged to minor isomers with an acetyloxy group on the (*n*–9) or (*n*–6) carbons.

We assumed that the abundance ratio of components with oxygen-containing groups on the (*n*–9), (*n*–8), (*n*–7), or (*n*–6) carbons (about 1:3:3:1, respectively) were nearly the same for isomeric hydrogenated products and initial hydroperoxy FA moieties. This suggestion was supported by (–)ESI-MS/MS study of isomeric allylic hydroperoxides (Figure 4a), showing two pairs of characteristic peaks with a 3:1 intensity ratio, including peaks at *m*/*z* 743.6/729.55 (fragments, formed from acyl chains with –OOH in the (*n*–8)/(*n*–9) positions) and *m*/*z* 784.6/798.6 (fragments, formed from acyl chains with –OOH in the (*n*–7)/(*n*–6) positions). Thus, the previously mentioned minor hydrogenated products were possibly derived from minor amide-linked FA with allylic hydroperoxy/hydroxy/keto groups in the (*n*–9) or (*n*–6) positions. However, complete structures of the corresponding oxidized cerebrosides could not be comprehensively elucidated due to their minor amounts. Therefore, only major cerebrosides with oxygen-containing groups in the (*n*–8) or (*n*–7) positions of acyl chains were the focus of our research.

## Appendix C

Methyl esters of 11-hydroxy and 8-hydroxy elaidic acids were prepared from methyl oleate according to the method described by Li et al. [46]. Methyl 11-hydroxy elaidate was treated with DMDS to give a mixture, consisting of 59.2% of the expected DMDS adduct, 26.8% of isomeric allylic thioethers (9-(methylthio)octadec-10-enoate and 11-(methylthio)octadec-9-enoate), and 8.0% of 9,10,11-tris(methylthio)octadecenoate (Scheme A1). Two GC peaks that showed identical major fragments in GC-MS represented stereoisomeric *bis*(methylthio) adducts with a nearly 1:1 ratio. Assuming a specific anti-addition of DMDS [33], these stereoisomeric adducts could be racemic erythro-9,10-bis(methylthio)octadecanoates, differing in configuration only at the carbon atom linked to the –OH group. Based on the NOESY experiment, two pairs of singlets at *δ*_Н_ 2.14, 2.22 and *δ*_Н_ 2.06, 2.185 in the ^1^H-NMR spectrum (CDCl_3_) of the mixture were assigned to vicinal –SMe groups of different racemates. Two GC peaks (major and minor) for mono(methylthio)octadecenoates also had identical mass spectra. For major *trans*-allylic thioethers, the ^1^H,^1^H-COSY diagram (CDCl_3_) showed a spin system, which consisted of the protons of two *trans*-olefinic CH (*δ*_Н_ 5.17, dd, *J* = 9.2, 15.2 Hz, and 5.40, dt, *J* = 6.8, 15.2 Hz) and CH (*δ*_Н_ 2.985, m), bearing an –SMe group (^1^H-NMR spectrum: *δ*_H_ 1.965, s). Accordingly, the major GC peak for overlapping 9-(methylthio)octadec-10-enoates and 11-(methylthio)octadec-9-enoates represented isomers with a *trans*-double bond (23.0%), and the minor GC peak, observed before the major peak, likely represented their *cis*-isomers (3.8%). Similarly, two GC peaks (major and minor) for stereoisomeric 9,10,11-tris(methylthio)octadecanoates were observed. Upon MeCN/HCl hydrolysis of this methylthio derivative mixture, the stereoisomeric bis(methylthio) adducts were destroyed, so major allylic thioethers (9/11-(methylthio)octadec-10/9-enoic acids, 59.3%) and minor tris(methylthio) derivatives (stereoisomeric 9,10,11-tris(methylthio)octadecanoic acids, 12.4%), analogous to that shown in Scheme 3, along with octadecadienoic acid (11.7%), were found in the hydrolysate. Apparently, the bis(methilthio) adduct of allylic alcohol could lose an –OH group and one –SMe group during MeCN/HCl hydrolysis, giving rise to an additional amount of allylic thioethers.

The DMDS adduct of methyl oleate can be de-esterified in MeCN/HCl without detectable degradation of the –CH(SMe)–CH(SMe)– fragment, in contrast to the DMDS adduct of methyl 11-hydroxy elaidate. This was confirmed by the ^1^Н-NMR spectra, recorded before and after hydrolysis. In particular, the ^1^Н-NМR spectrum (CDCl_3_) of the product, obtained after hydrolysis of the DMDS adduct of methyl oleate, showed the superimposed signals of two vicinal CH (*δ*_Н_ 2.685, m), linked to –SMe groups (*δ*_H_ 2.10, s), and α-CH_2_ (*δ*_Н_ 1.845, m; 1.32, m) groups of *bis*(methylthio) oleic acid. Analogously, the *bis*(methylthio) derivatives of monoenoic sphingoid bases were obtained after MeCN/HCl hydrolysis of derivatized cerebrosides (part 2).

As for the allylic alcohol acetate, methyl 8-acetyloxy elaidate did not react appreciably with DMDS under the conditions used for methyl 11-hydroxy elaidate (room temperature, 24 h). However, with longer reaction times (4 days), methyl 8-acetyloxy elaidate reacted with DMDS to give major allylic ethers, 8-(methylthio)octadec-9-enoate and 10-(methylthio)octadec-8-enoate (61.5%, mainly *trans*-forms), and minor 8,9,10-tris(methylthio)octadecanoates (25.4%), formed after deacetylation of the starting compound. Surprisingly, the expected bis(methylthio)derivative of methyl 8-acetyloxy elaidate was not detected (Scheme A1). For experimental details and mass spectra (Figures S33–S37), see the supplementary materials.
