# Supplementary material for: Structural Analysis of Oxidized Cerebrosides from the Extract of Deep-Sea Sponge Aulosaccus sp.: Occurrence of Amide-Linked Allylically Oxygenated Fatty Acids
Source: Molecules. 2020 Dec 21;25(24):6047. doi: 10.3390/molecules25246047 (PMC7767537; doi:10.3390/molecules25246047)
Supplement: Supplementary file 1 [file molecules-25-06047-s001.pdf]

# *Supplementary Materials to the Article*

## Structural Analysis of Oxidized Cerebrosides from the Extract of Deep-Sea Sponge *Aulosaccus* sp.: Occurrence of Amide-Linked Allylically Oxygenated Fatty Acids

**Elena A. Santalova*, Vladimir A. Denisenko and Pavel S. Dmitrenok**

**Content**

**Figure S1a**: Base peak chromatogram from UPLC-MS analysis of oxidized cerebrosides (positive ion mode)……………………………………………..……………….3

**Figure S1b**: Extracted-ion chromatograms from UPLC-MS analysis of oxidized cerebrosides ……………………………………………………………….……………………4

**Scheme S1**: Hock fragmentation of some isomeric allylic hydroperoxides found in the present study………..……………………………………………………………….……5

**Figures S2, S2a–S2c**: $^{1}$H-NMR spectrum ($CD_3OD$, 500 MHz) of RP-HPLC fraction, containing oxidized cerebrosides……………...…………………………………..6–9

**Figure S3:** $^{13}$C-NMR spectrum ($CD_3OD$, 125 MHz) of RP-HPLC fraction, containing oxidized cerebrosides………...………………………………………………………..10

**Figure S4–S7**: Mass spectra of samples enriched in the methyl esters of 2-acetyloxy $C_{23}$ and $C_{22}$ acids with a keto group in the (*n*–8) or (*n*–7) positions……..…….11–14

**Figures S8–S11**: Mass spectra of samples enriched in the methyl esters of 2-acetyloxy $C_{23}$ and $C_{22}$ acids with an additional acetyloxy group in the (*n*–8) or (*n*–7) positions…………………………………………………………………………………………………………………………………………………………………15–18

**Figures S12, S13:** Mass spectra of samples enriched in the methyl esters of 2-acetyloxy $C_{23}$ acids with a keto group in the (*n*–9) or (*n*–6) positions……..…………..19, 20

**Figure S14:** Averaged mass spectrum for four methyl esters of isomeric 2-acetyloxy keto $C_{23}$ acids……………………………...………………………………21

**Figures S15, S16:** Mass spectra of samples enriched in the methyl esters of 2-acetyloxy $C_{22}$ acids with a keto group in the (*n*–9) or (*n*–6) positions……..…..………22, 23

**Figures S17–S20**: Mass spectra of samples enriched in the methyl esters of 2-acetyloxy $C_{23}$ and $C_{22}$ acids with an additional acetyloxy group in the (*n*–9) or (*n*–6) positions……………………………………………………………………………………………………………………………………………….……………24–27

**Figures S21–S28**: Mass spectra of samples enriched in the methyl esters of 2-acetyloxy $C_{23}$ and $C_{22}$ acids with an allylic methylthio group……………………..…..28–35

**Figures S29–S32**: Mass spectra of the methyl esters of 2-acetyloxy *tris*(methylthio) $C_{23}$ and $C_{22}$ acids……………………………………….……………………36–39

Allylic monohydroxylation of methyl oleate. Methylthiolation of allylic hydroxy/acetyloxy elaidates……………………………………………………………………40

**Figure S33**: Mass spectrum of the *bis*(methylthio) adduct of methyl 11-hydroxy elaidate……………………………………………………………………………..42

**Figure S34**: Superimposed mass spectra of overlapping methyl 9-(methylthio)octadec-10-enoate and 11-(methylthio)octadec-9-enoate………………………………43

**Figure S35**: Mass spectrum of methyl 9,10,11-*tris*(methylthio)octadecanoate……………………………………………………………………………………...44

**Figure S36**: Superimposed mass spectra of overlapping methyl 10-(methylthio)octadec-8-enoate and 8-(methylthio)octadec-9-enoate…………………………..……45

**Figure S37**: Mass spectrum of methyl 8,9,10-*tris*(methylthio)octadecanoate……………………………………………………………………………..…..…46

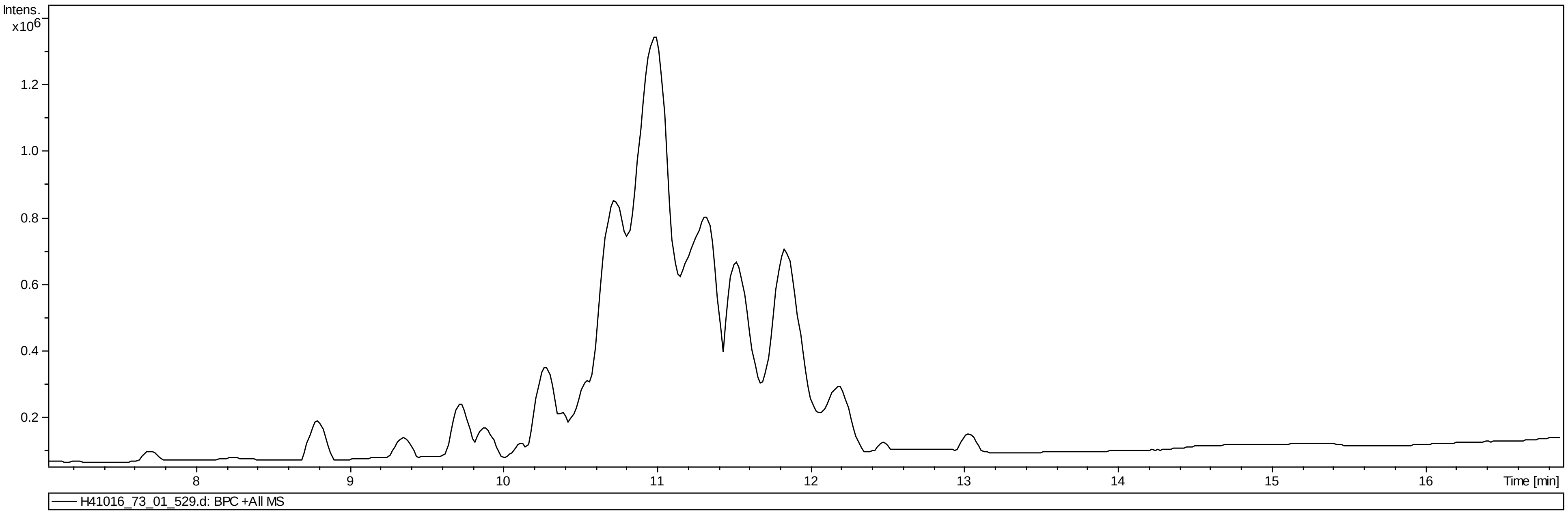


**Figure S1a**. Base peak chromatogram from UPLC-MS analysis of oxidized cerebrosides (positive ion mode).

Analysis was performed using a Bruker Elute UPLC chromatograph (Bruker Daltonics, Bremen, Germany) connected to a Q-TOF 6510 mass spectrometer (Agilent Technologies, Santa Clara, CA, USA). InfinityLab Poroshell 120 SB-C18 column (2.1 × 150 mm, 2.7 µm, Agilent Technologies, Santa Clara, CA, USA) was used for chromatographic separation. The mobile phases were 0.1% formic acid in $H_2O$ (eluent A) and 0.1% formic acid in MeCN (eluent B). The gradient program was as follows: from 80% to 100% eluent B from start to 25 min, isocratic at 100% of eluent B to 30 min, from 100% to 80% eluent B from 30 to 30.5 min. After returning to the initial conditions, the equilibration was achieved after 5 min. Chromatographic separation was performed at a 0.3 mL/min flow rate at 45 °C. Injection volume was 5 µL.

The mass spectrometry detection was performed using ESI ionization source. Optimized ionization parameters for ESI were as follows: a capillary voltage of 4.5 kV, nebulization with nitrogen at 2.5 bar, dry gas flow of 6 L/min at a temperature of 200 °C. The mass spectra were recorded in positive ion mode within *m/z* mass range of 50–1500. Collision induced dissociation (CID) product ion mass spectra were recorded in auto-MS/MS mode with a collision energy ranging from 75 to 100 eV (an exact collision energy setting depended on the molecular masses of precursor ions). The precursor ions were isolated with an isolation width of 4 Th. The mass spectrometer was calibrated using the ESI-L Low Concentration Tuning Mix (Agilent Technologies, Santa Clara, CA, USA).

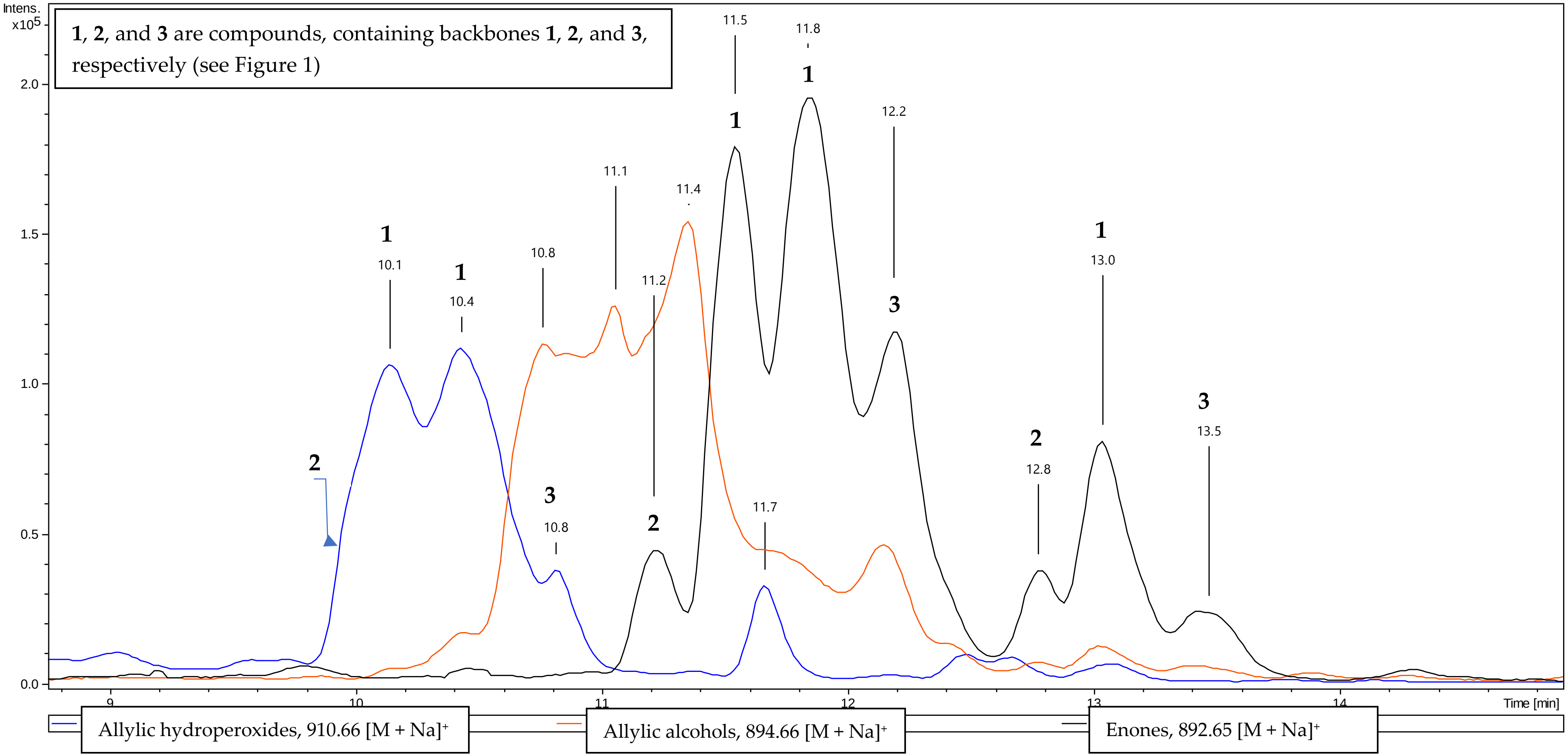


**Figure S1b**. Extracted-ion chromatograms from UPLC-MS analysis of oxidized cerebrosides. In UPLC-MS/MS, compounds **1**, **2**, and **3** fragmented to give *O* ions, containing backbones **1** (*m*/*z* 528), **2** (*m*/*z* 528), and **3** (*m*/*z* 542), respectively. Minor compound **2** was eluted slightly before isomeric compound **1**, as described for RP-HPLC separation of related compounds, containing different backbones and identical acyl chains [8]. Major clusters of peaks, corresponding to allylic hydroperoxides and enones, contain two partially resolved peaks (≈1:1) for major isomeric compounds **1a** and **1b** or **1a**// and **1b**//, respectively. Perhaps, each cluster consists of two overlapping "triplets" (for example, "triplets" **1a**//-**2a**//-**3a**// and **1b**//-**2b**//-**3b**// for the most resolved peaks of enones). Additionally, the presence of some minor peaks may be masked by peaks, representing isomers of dominant compounds **1** with rearranged acyl chains. For allylic alcohols, a cluster of closely overlapping chromatographic peaks was detected, presumably due to possible allylic rearrangements under acidic conditions of UPLC-MS analysis.

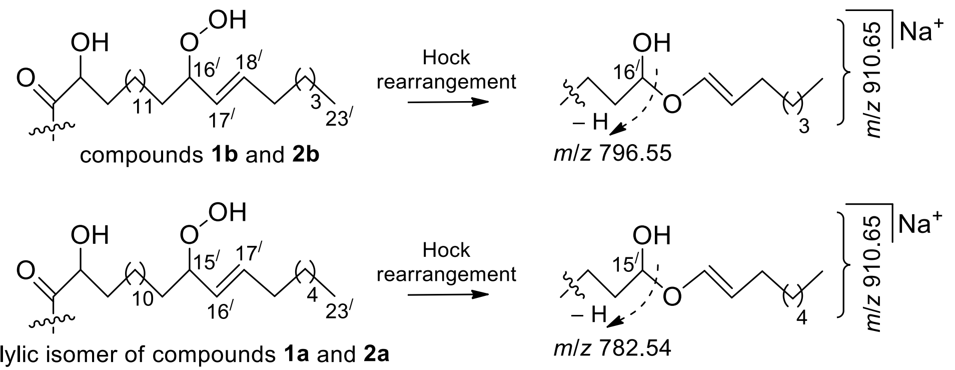


**Scheme S1**. Hock fragmentation of some isomeric allylic hydroperoxides found in the present study.

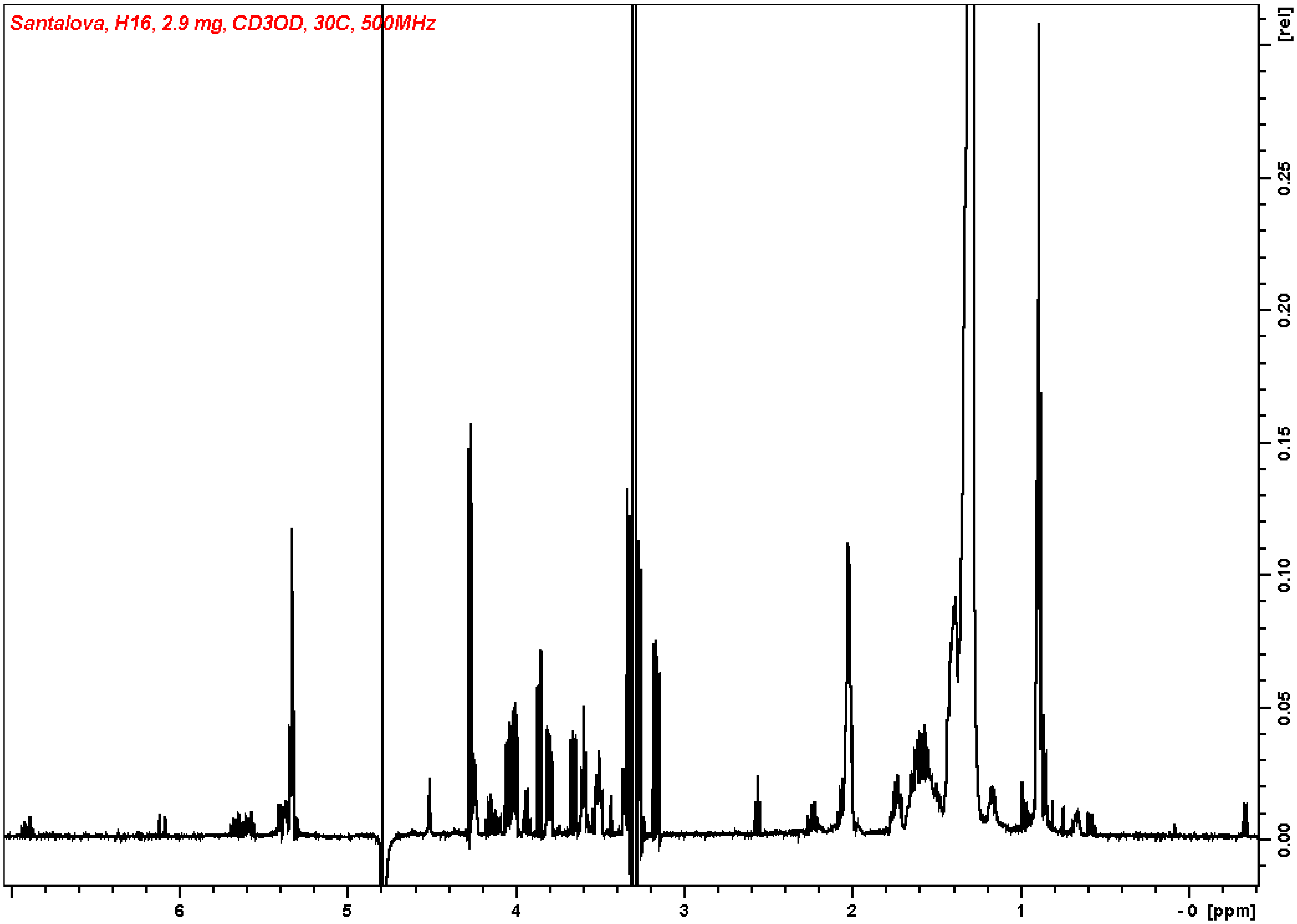


**Figure S2**. $^{1}$H-NMR spectrum ($CD_3OD$, 500 MHz) of RP-HPLC fraction, containing oxidized cerebrosides.

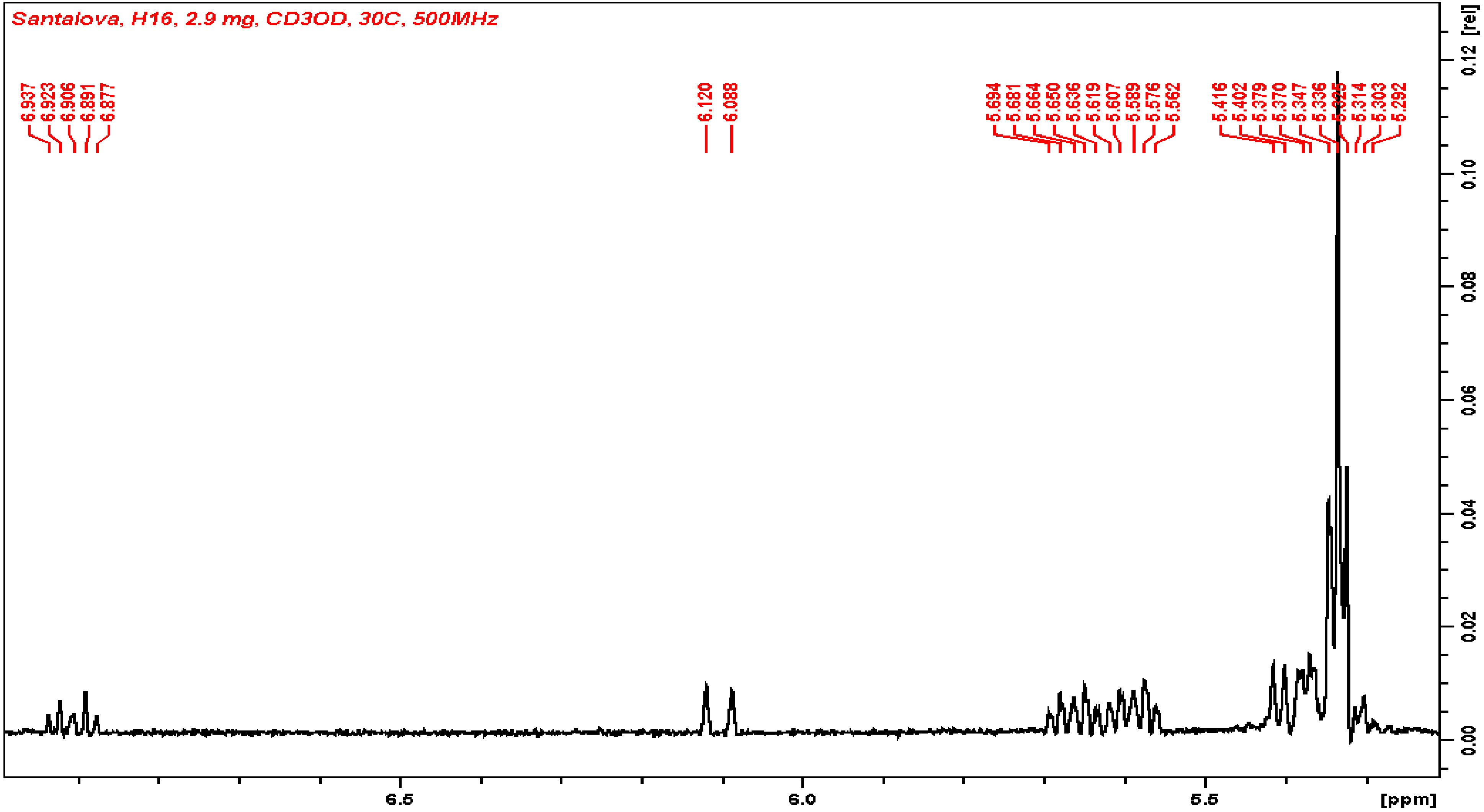


**Figure S2a**. Partial ¹H-NMR spectrum (CD3OD), containing olefinic proton signals.

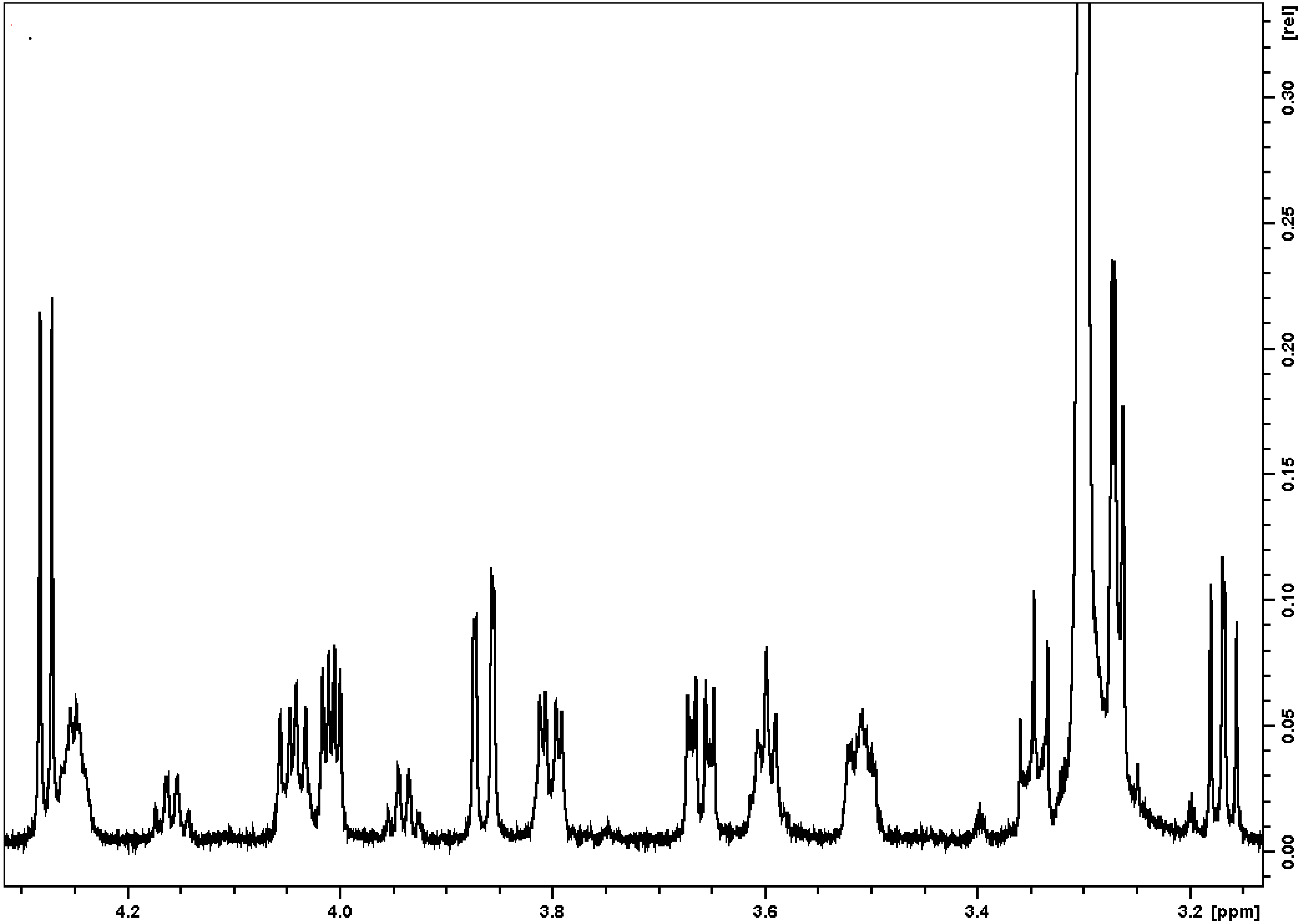


**Figure S2b**. Partial $^{1}$H-NMR spectrum ($CD_3OD$), containing mainly proton signals of oxygen-bearing fragments.

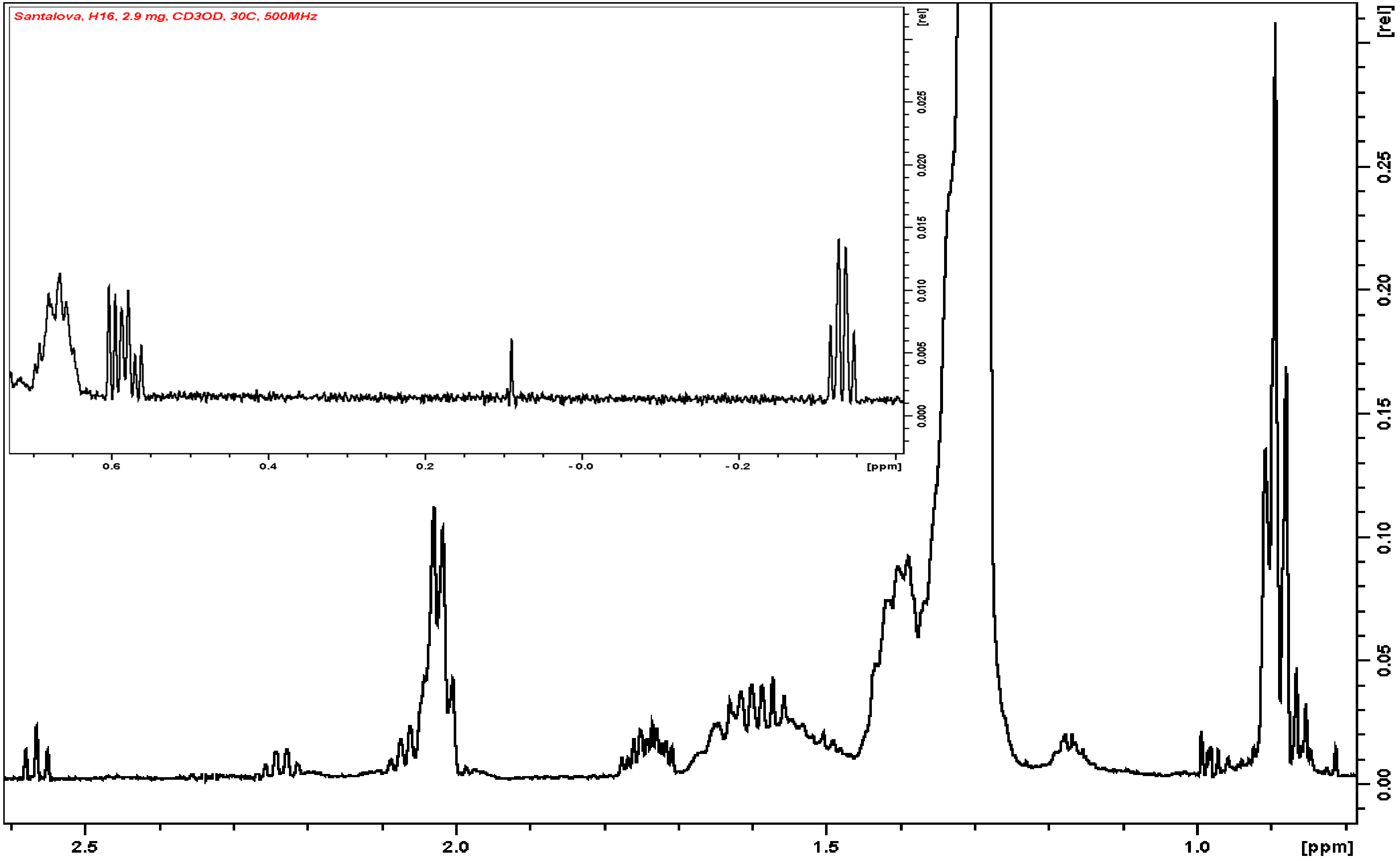


**Figure S2c**. Partial $^{1}H$-NMR spectrum ($CD_3OD$), including high-field region.

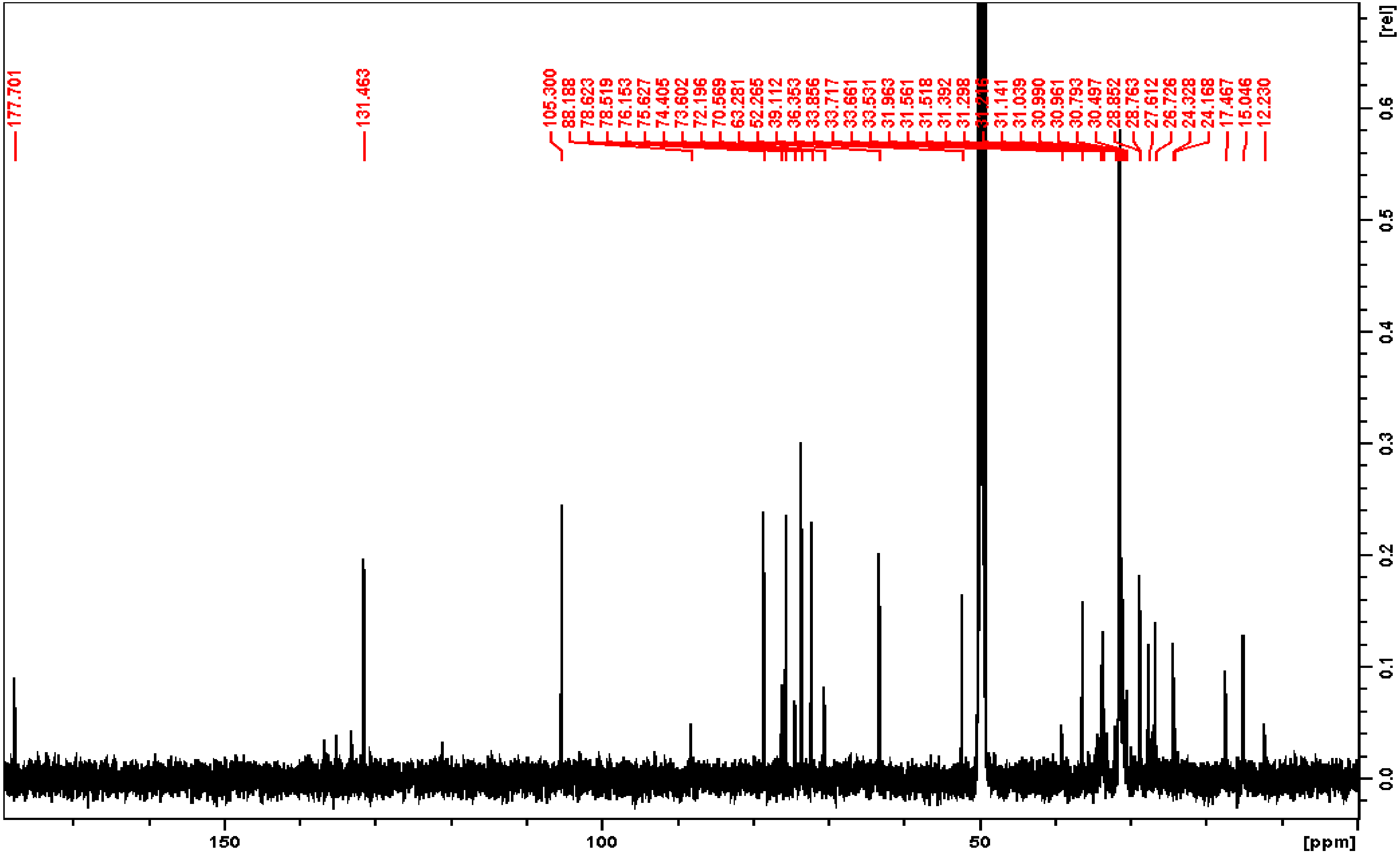


**Figure S3**. $^{13}$C-NMR spectrum ($CD_3OD$, 125 MHz) of RP-HPLC fraction, containing oxidized cerebrosides.

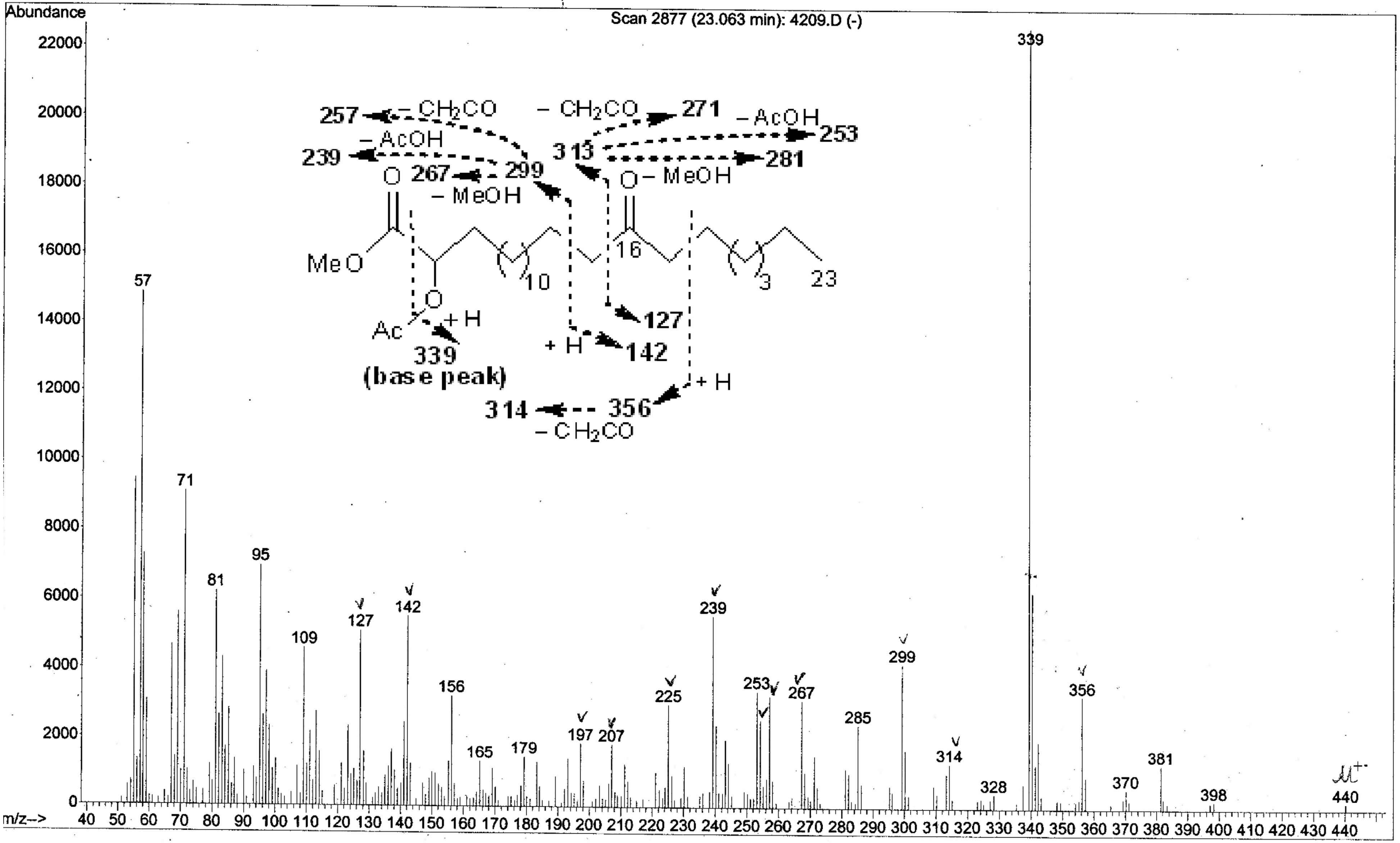


**Figure S4.** Mass spectrum of a sample enriched in the methyl ester of 2-acetyloxy $C_{23}$ acid with a keto group in the (*n*–8) position.

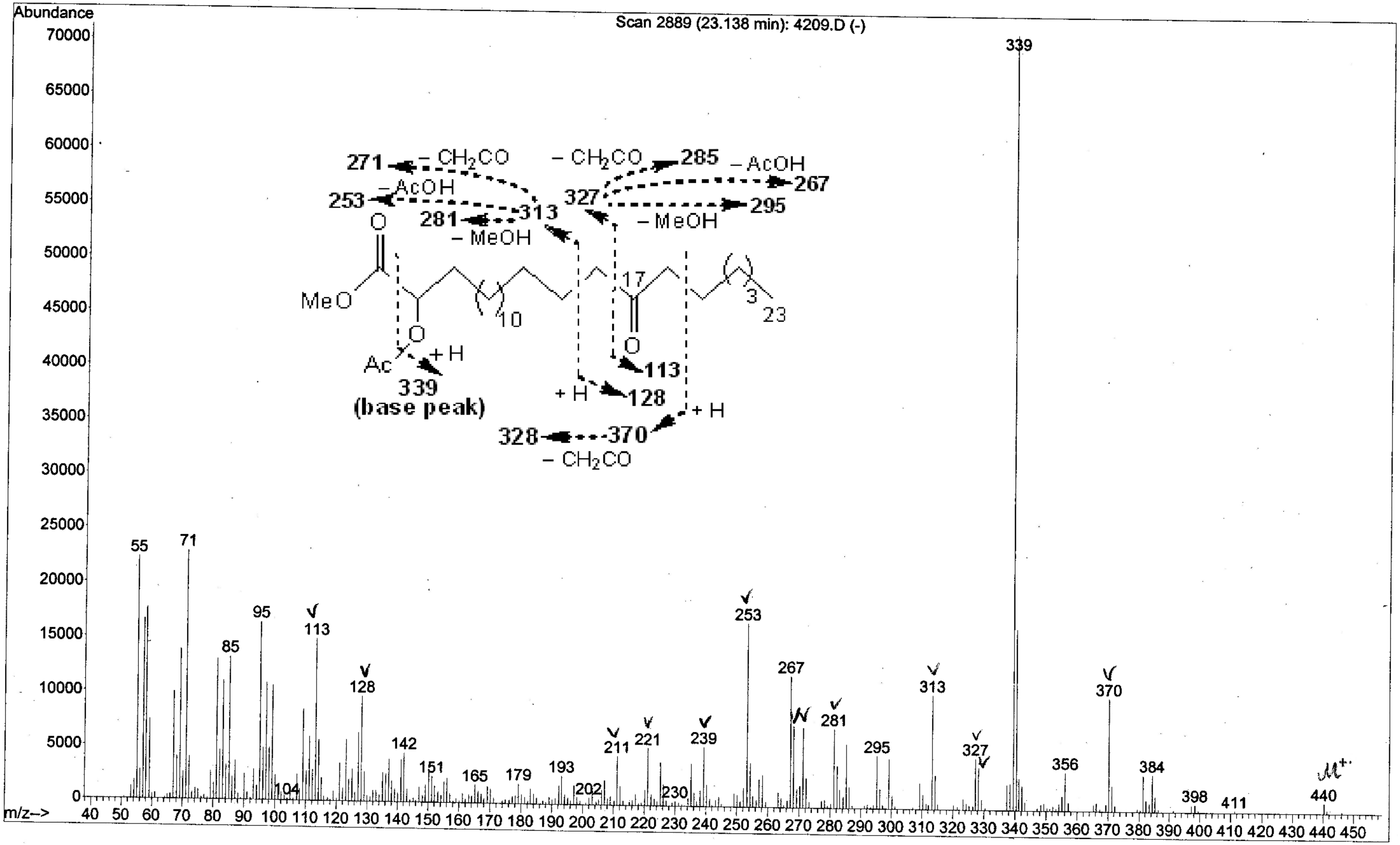


**Figure S5.** Mass spectrum of a sample enriched in the methyl ester of 2-acetyloxy $C_{23}$ acid with a keto group in the (*n*–7) position.

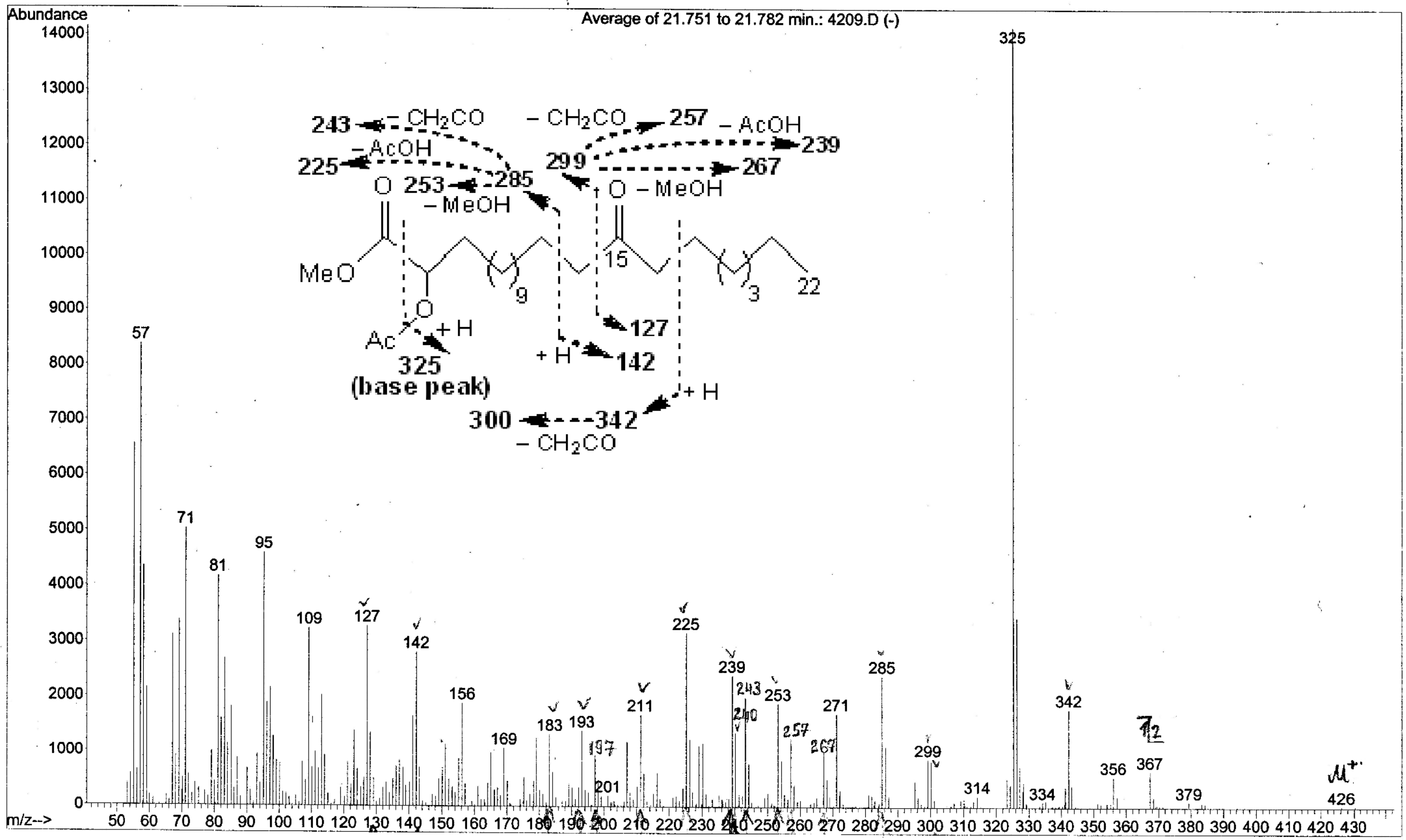


**Figure S6.** Mass spectrum of a sample enriched in the methyl ester of 2-acetyloxy $C_{22}$ acid with a keto group in the (*n*–8) position.

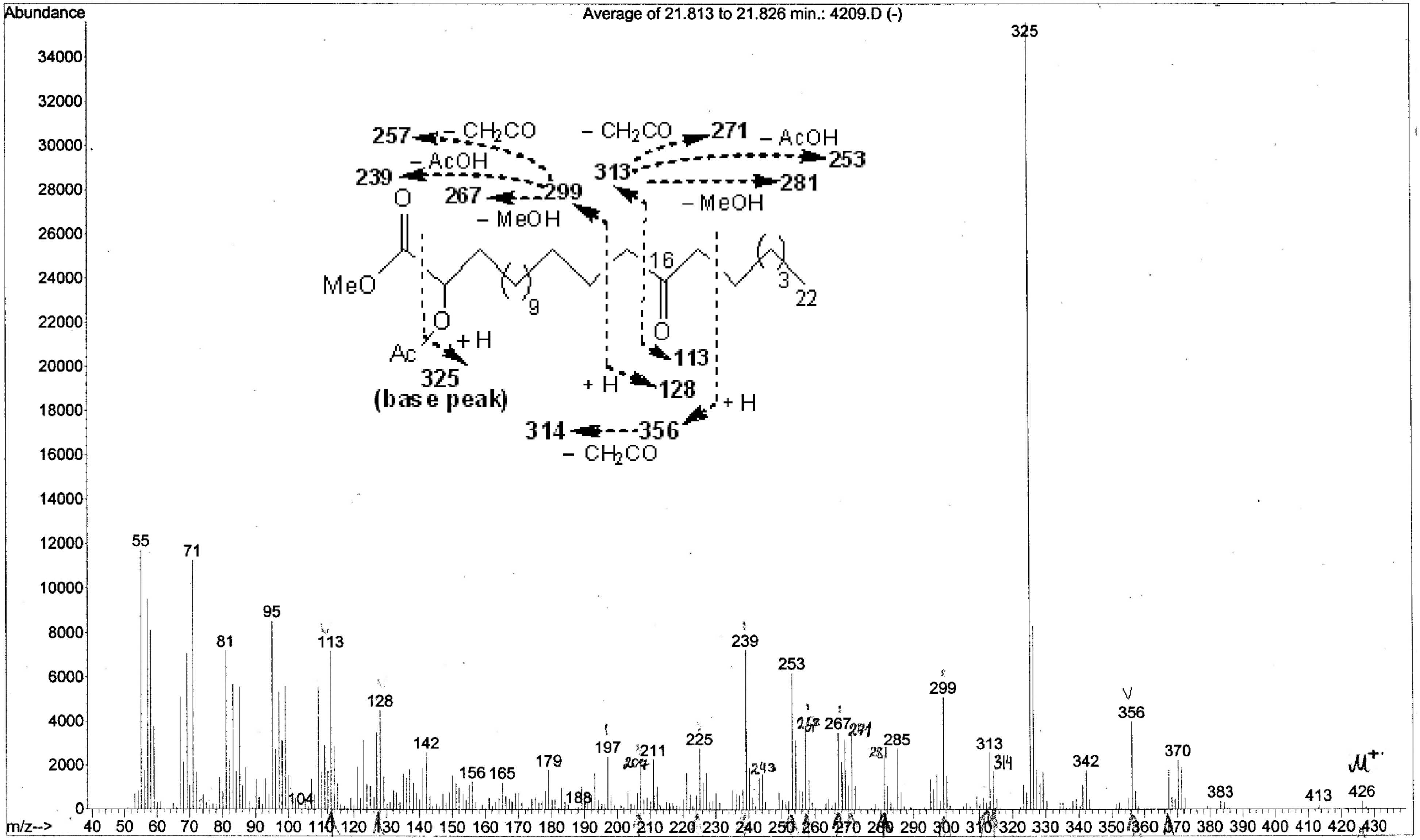


**Figure S7.** Mass spectrum of a sample enriched in the methyl ester of 2-acetyloxy $C_{22}$ acid with a keto group in the ($n$–7) position.

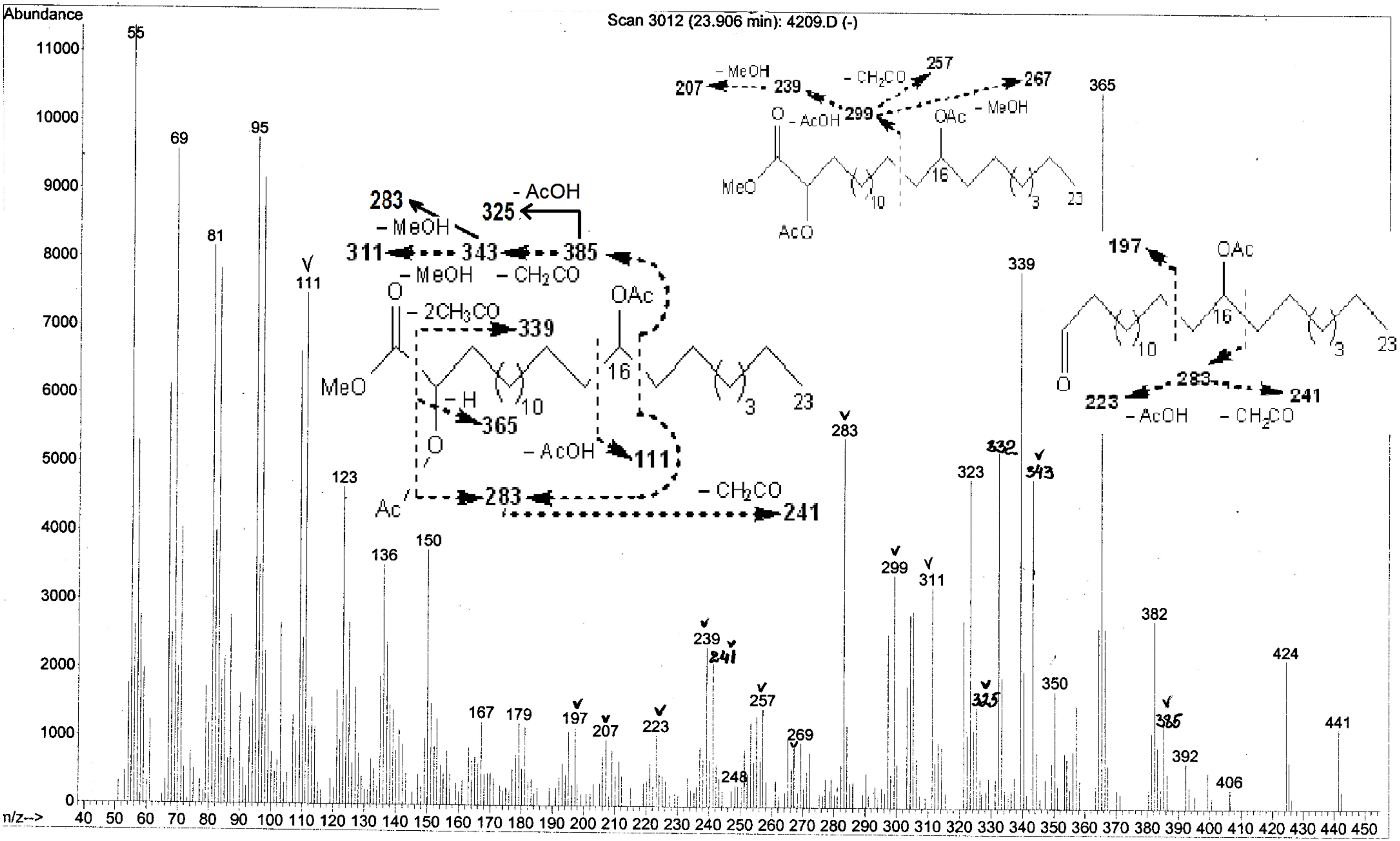


**Figure S8.** Mass spectrum of a sample enriched in the methyl ester of 2-acetyloxy $C_{23}$ acid with an additional acetyloxy group in the ($n$–8) position.

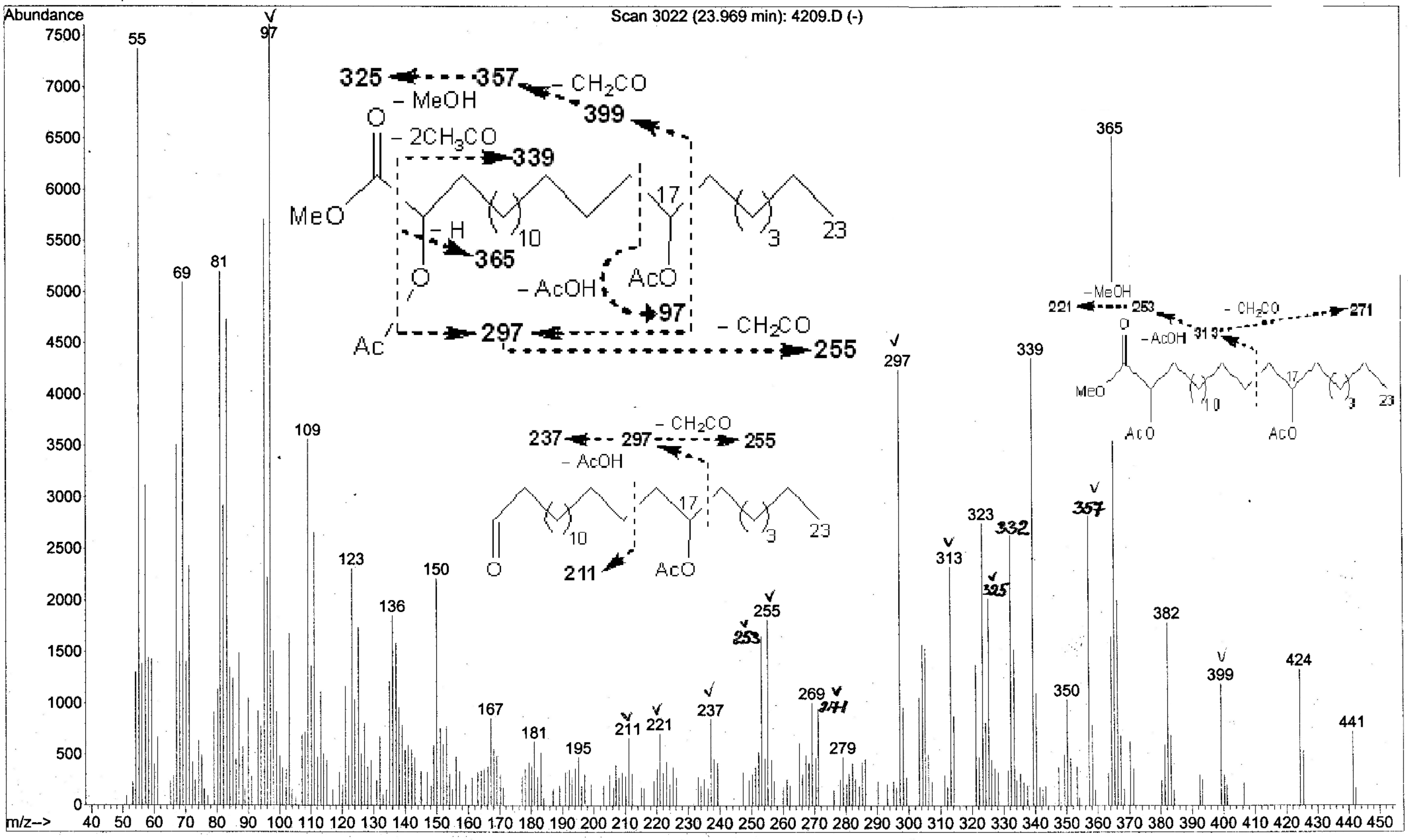


**Figure S9.** Mass spectrum of a sample enriched in the methyl ester of 2-acetyloxy $C_{23}$ acid with an additional acetyloxy group in the ($n$–7) position.

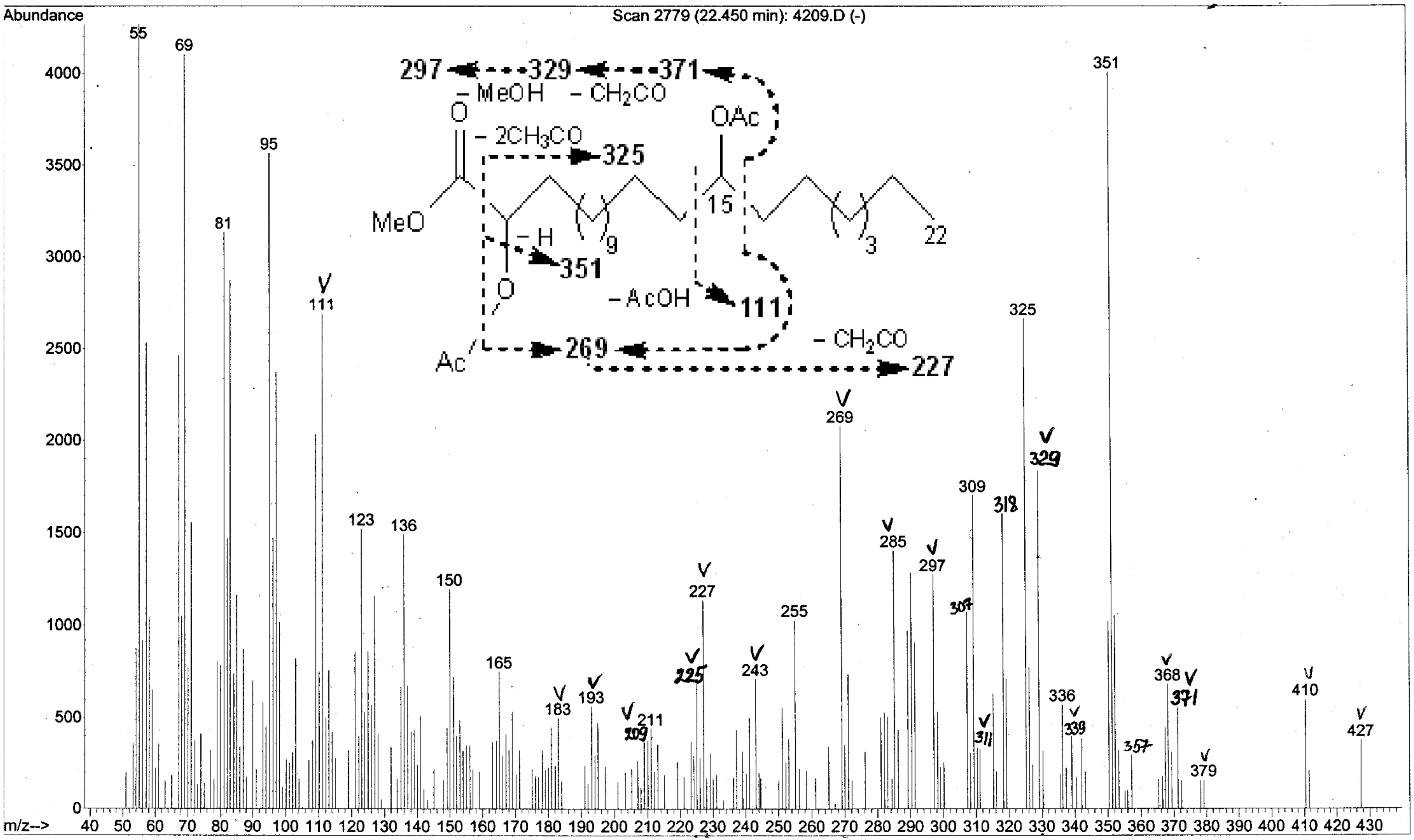


**Figure S10.** Mass spectrum of a sample enriched in the methyl ester of 2-acetyloxy $C_{22}$ acid with an additional acetyloxy group in the ($n$–8) position.

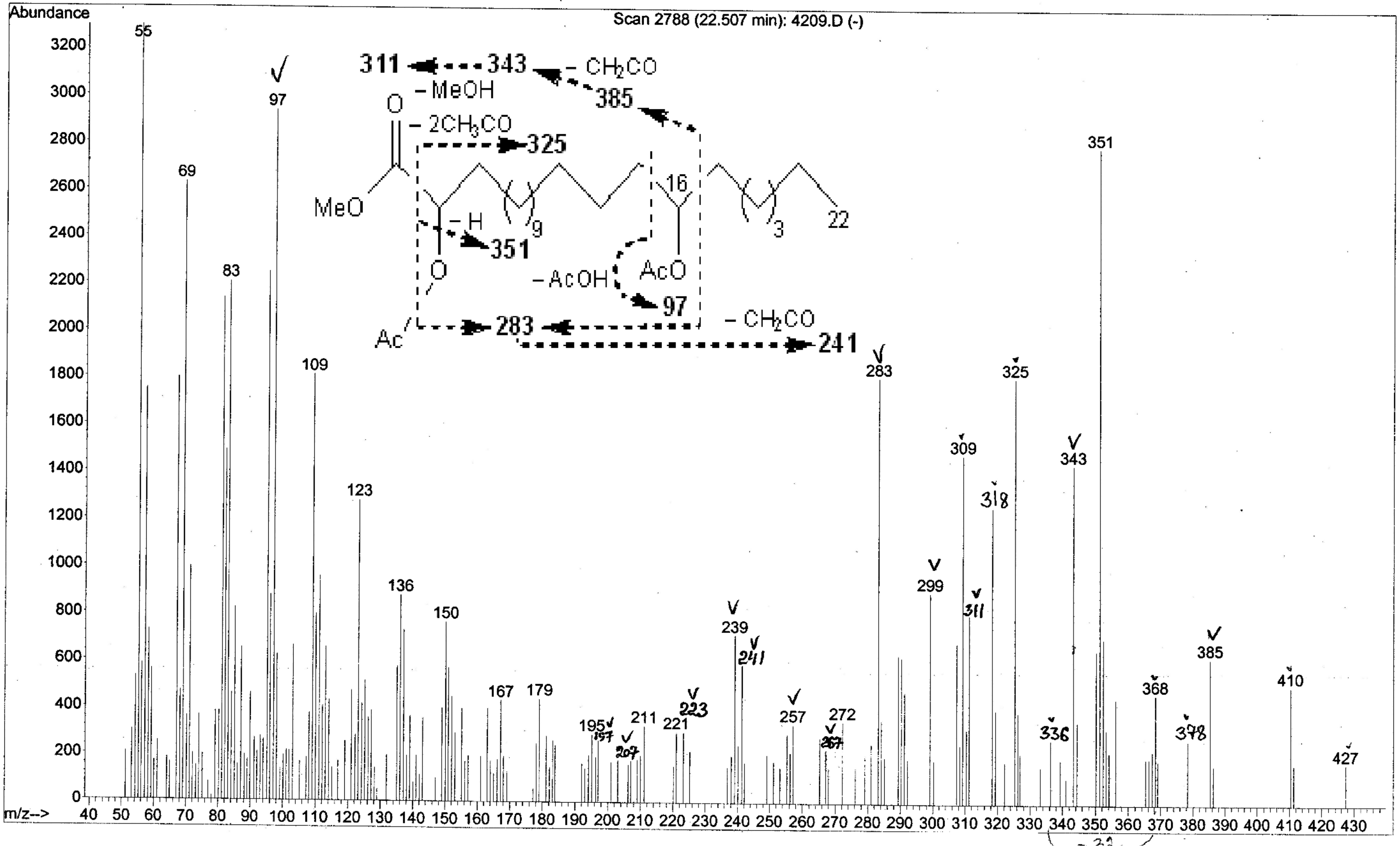


**Figure S11.** Mass spectrum of a sample enriched in the methyl ester of 2-acetyloxy $C_{22}$ acid with an additional acetyloxy group in the (*n*–7) position.

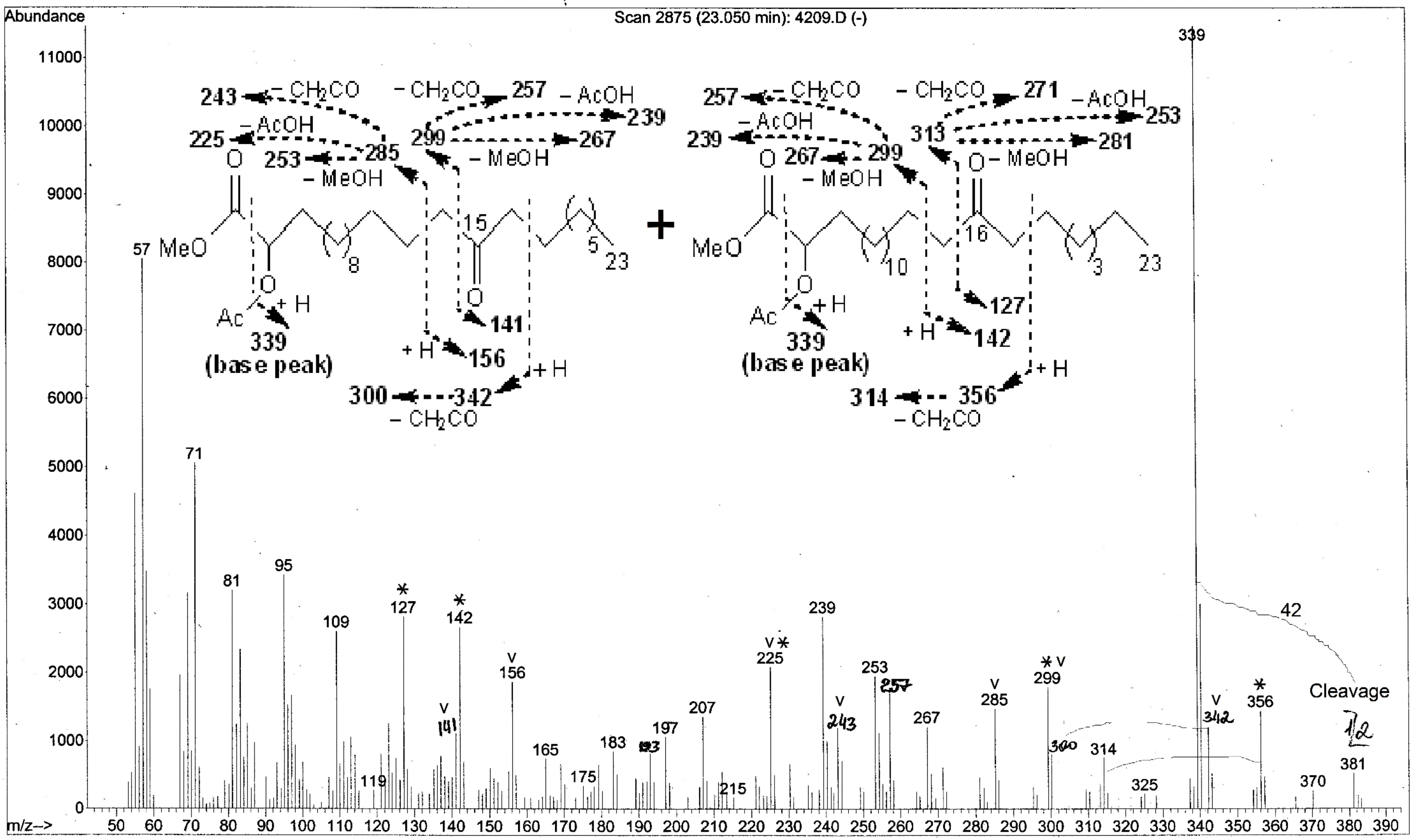


**Figure S12.** Mass spectrum of a mixture containing the overlapping methyl esters of 2-acetyloxy $C_{23}$ acids with a keto group in the (*n*–9) or (*n*–8) positions.

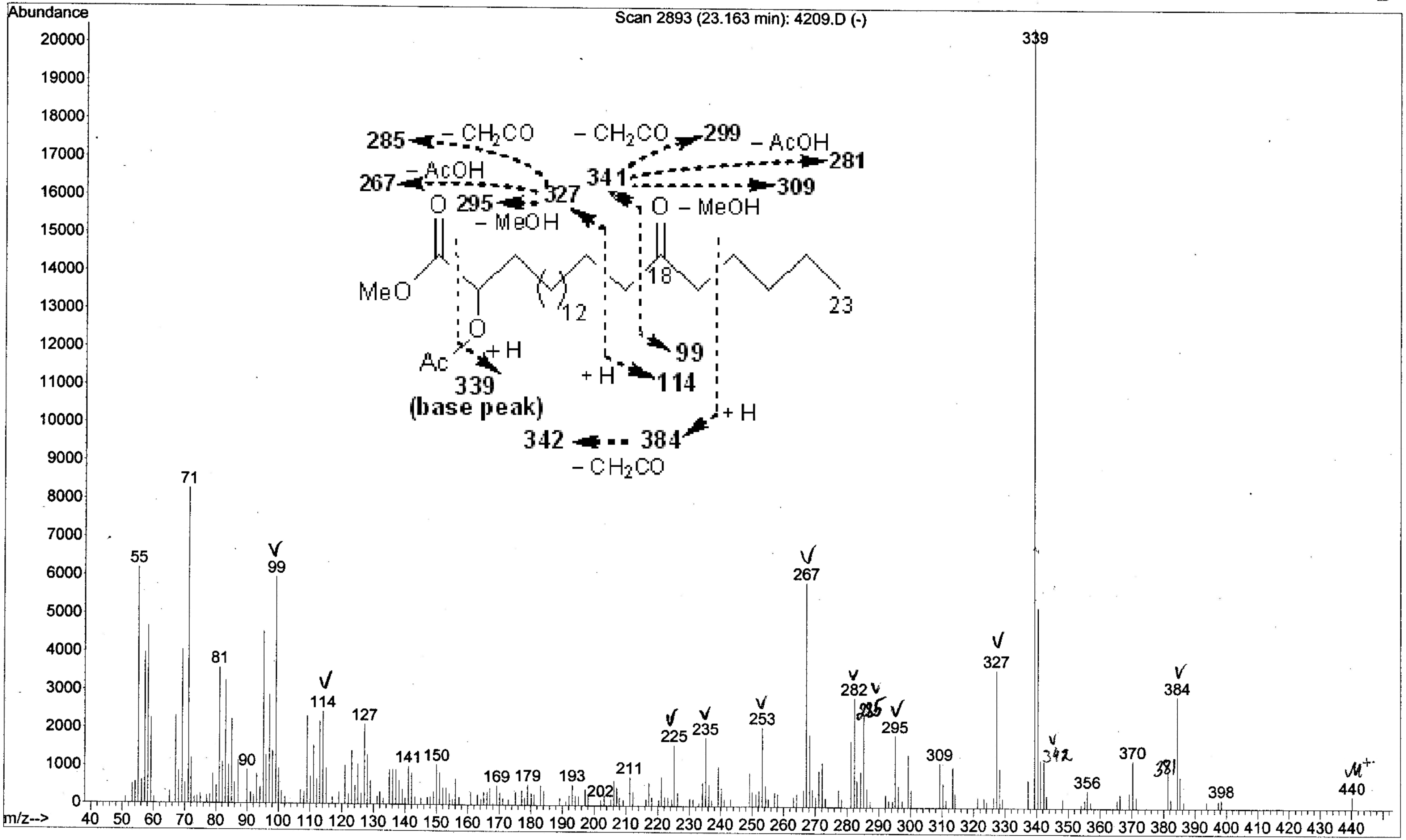


**Figure S13.** Mass spectrum of a sample enriched in the methyl ester of 2-acetyloxy $C_{23}$ acid with a keto group in the (*n*–6) position.

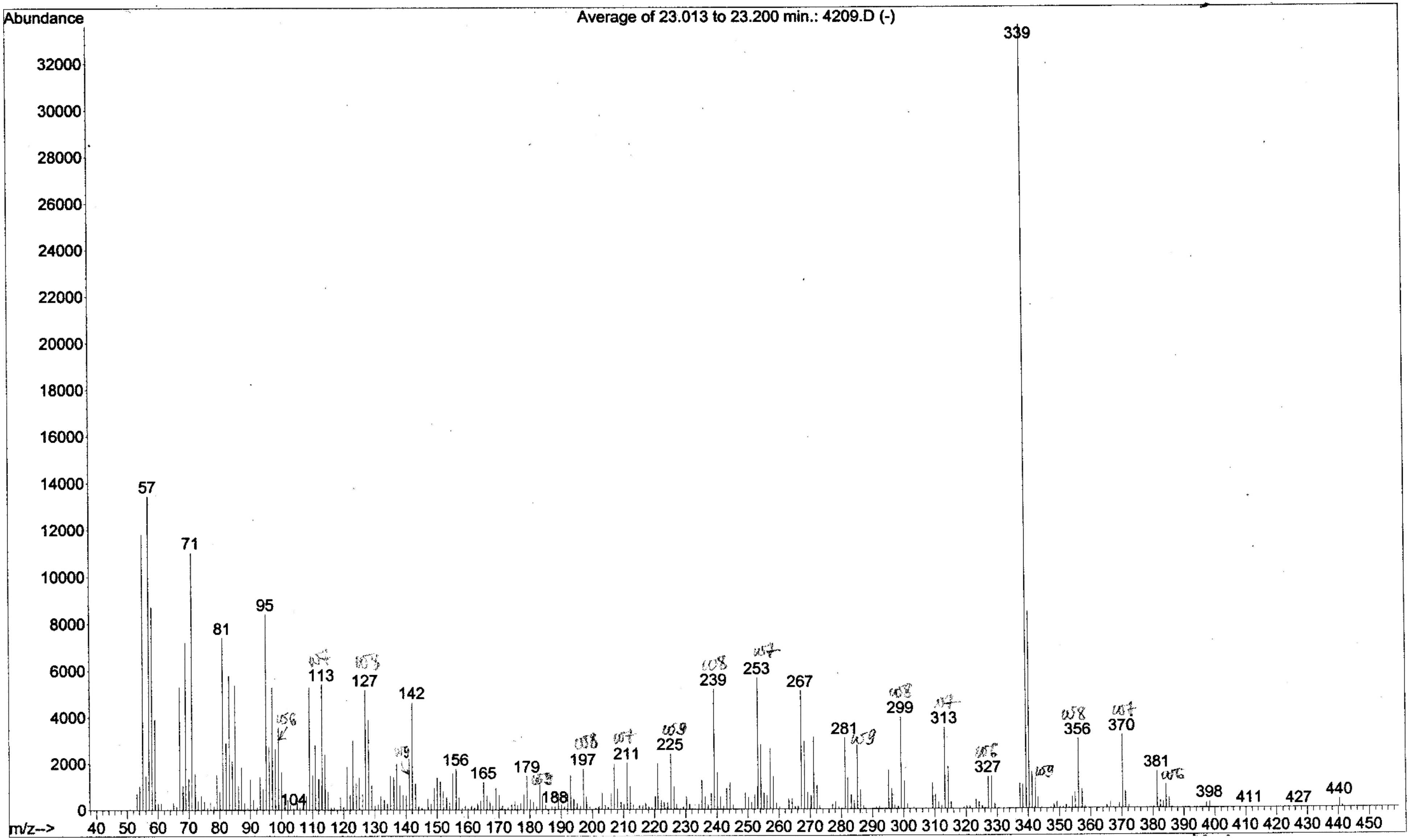


**Figure S14.** Averaged mass spectrum for four methyl esters of isomeric 2-acetyloxy keto $C_{23}$ acids.

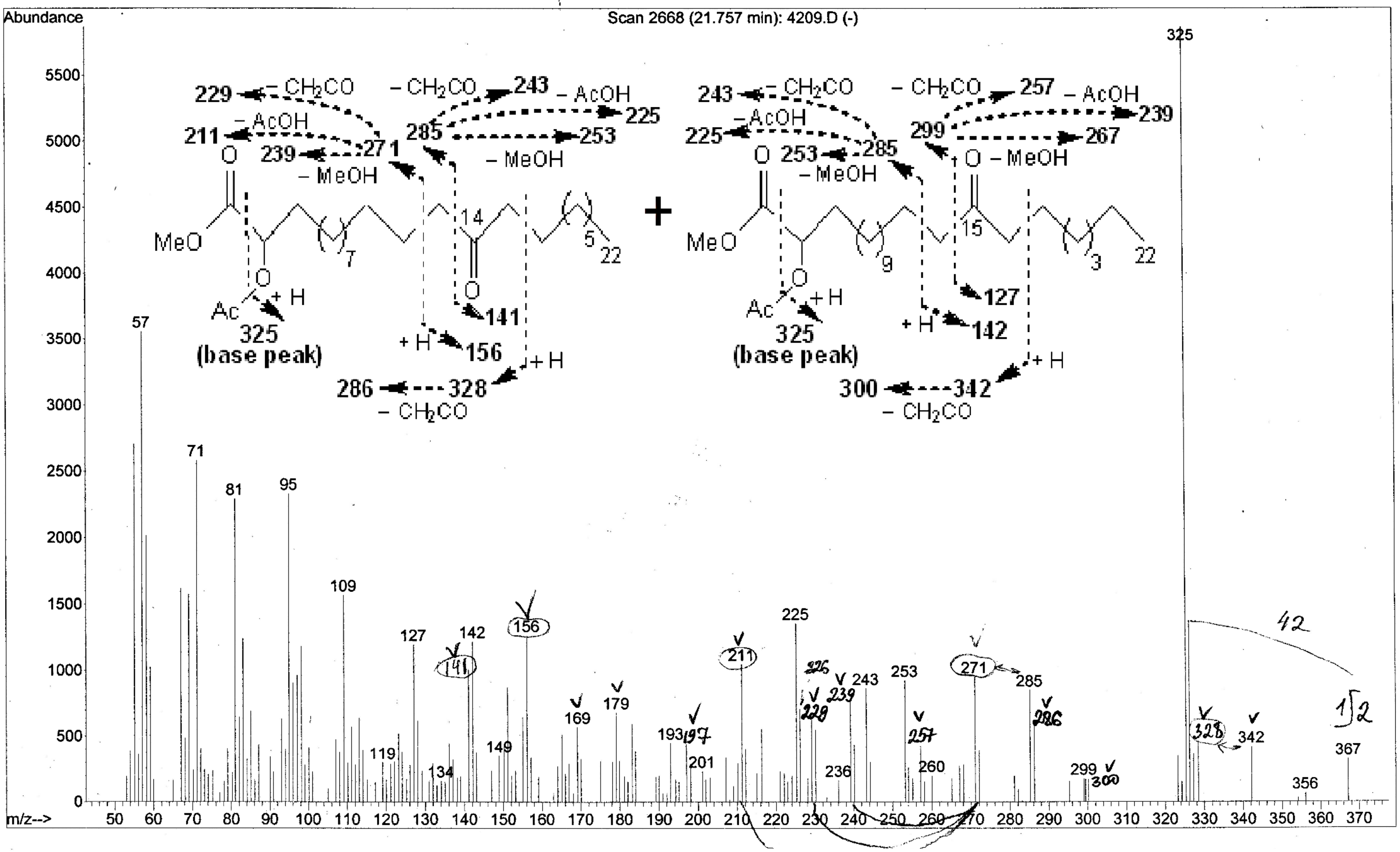


**Figure S15.** Mass spectrum of a mixture containing the overlapping methyl esters of 2-acetyloxy $C_{22}$ acids with a keto group in the (*n*–9) or (*n*–8) positions.

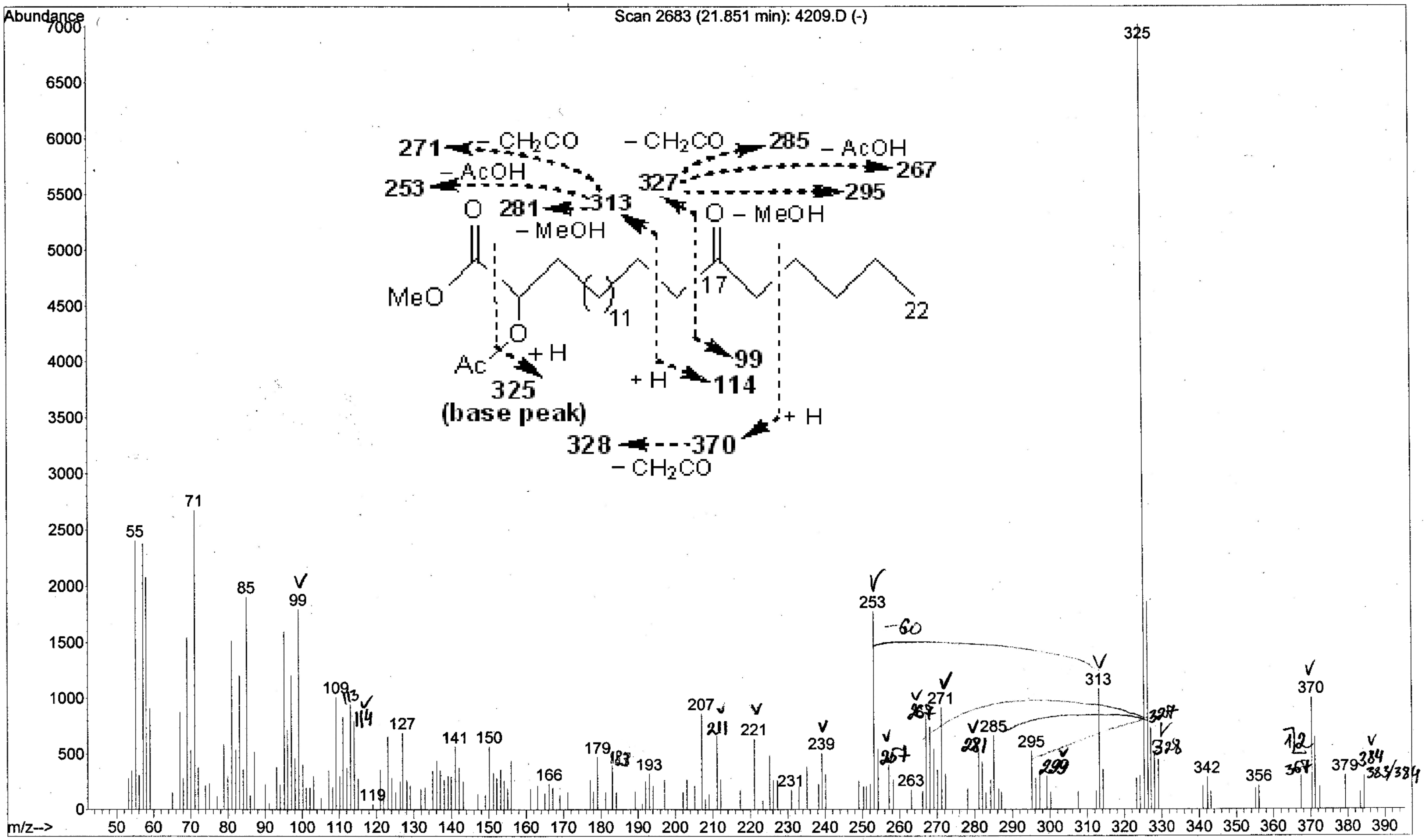


**Figure S16.** Mass spectrum of a sample enriched in the methyl ester of 2-acetyloxy $C_{22}$ acid with a keto group in the (*n*–6) position.

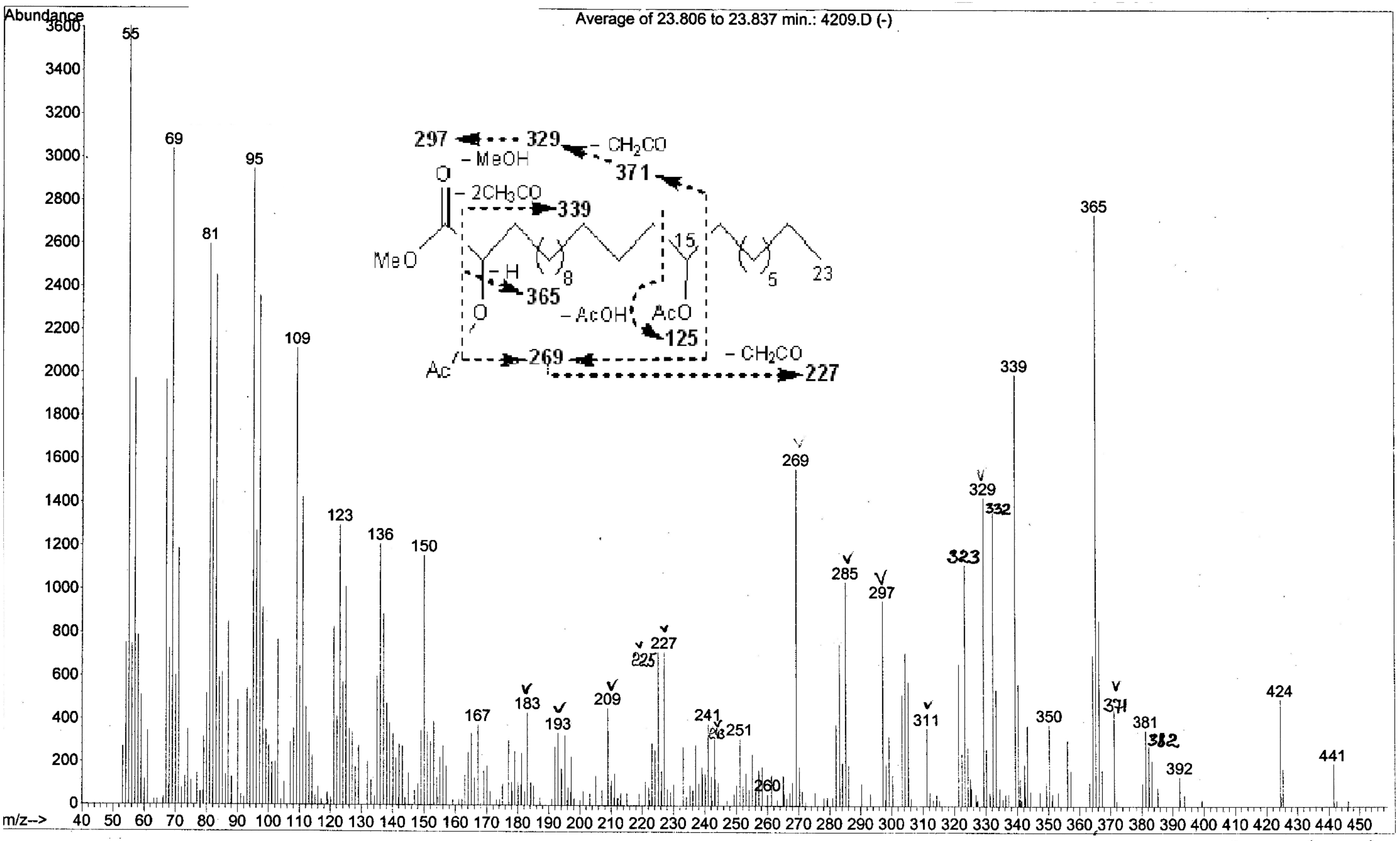


**Figure S17.** Mass spectrum of a sample enriched in the methyl ester of 2-acetyloxy $C_{23}$ acid with an additional acetyloxy group in the (*n*–9) position.

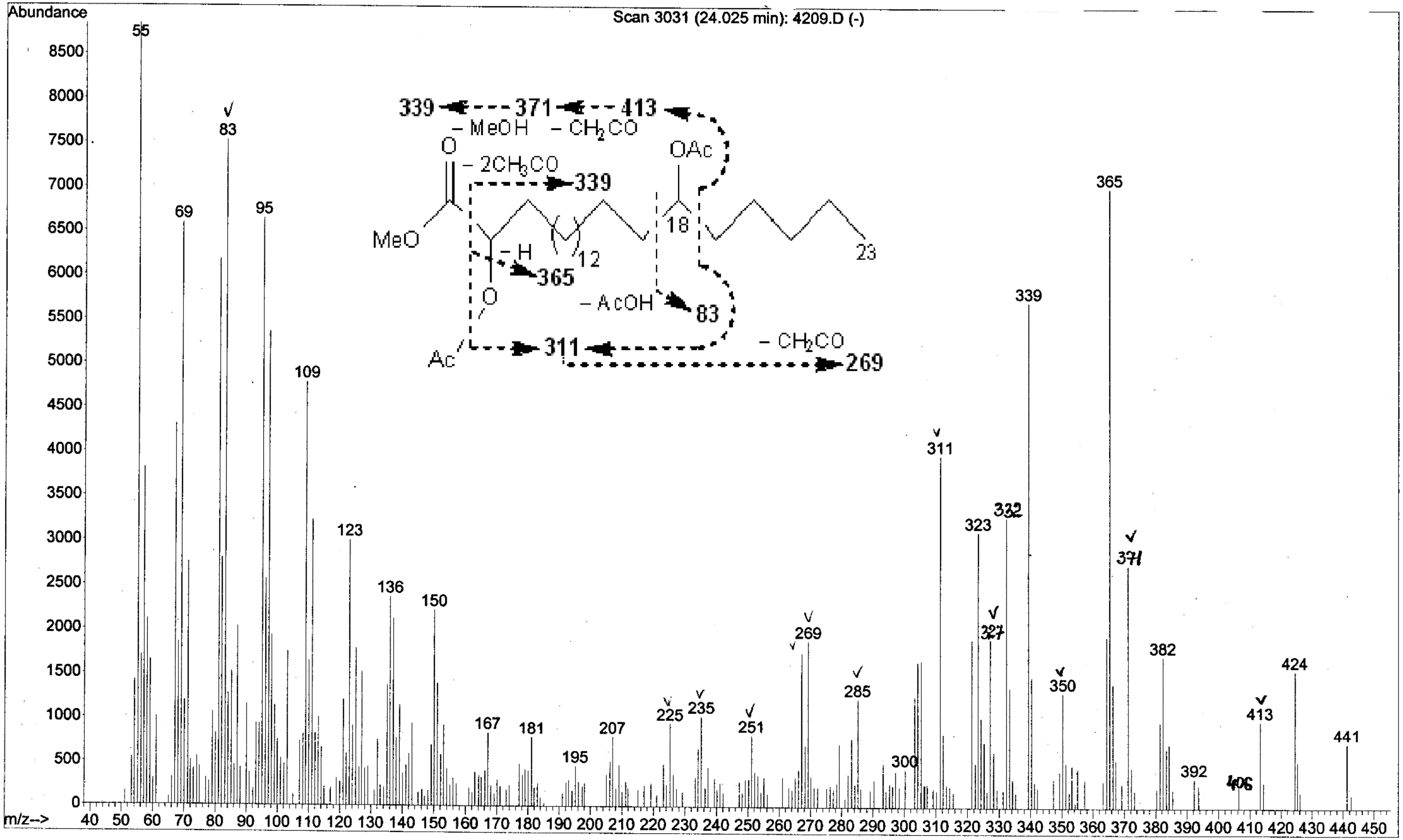


**Figure S18.** Mass spectrum of a sample enriched in the methyl ester of 2-acetyloxy $C_{23}$ acid with an additional acetyloxy group in the (*n*–6) position.

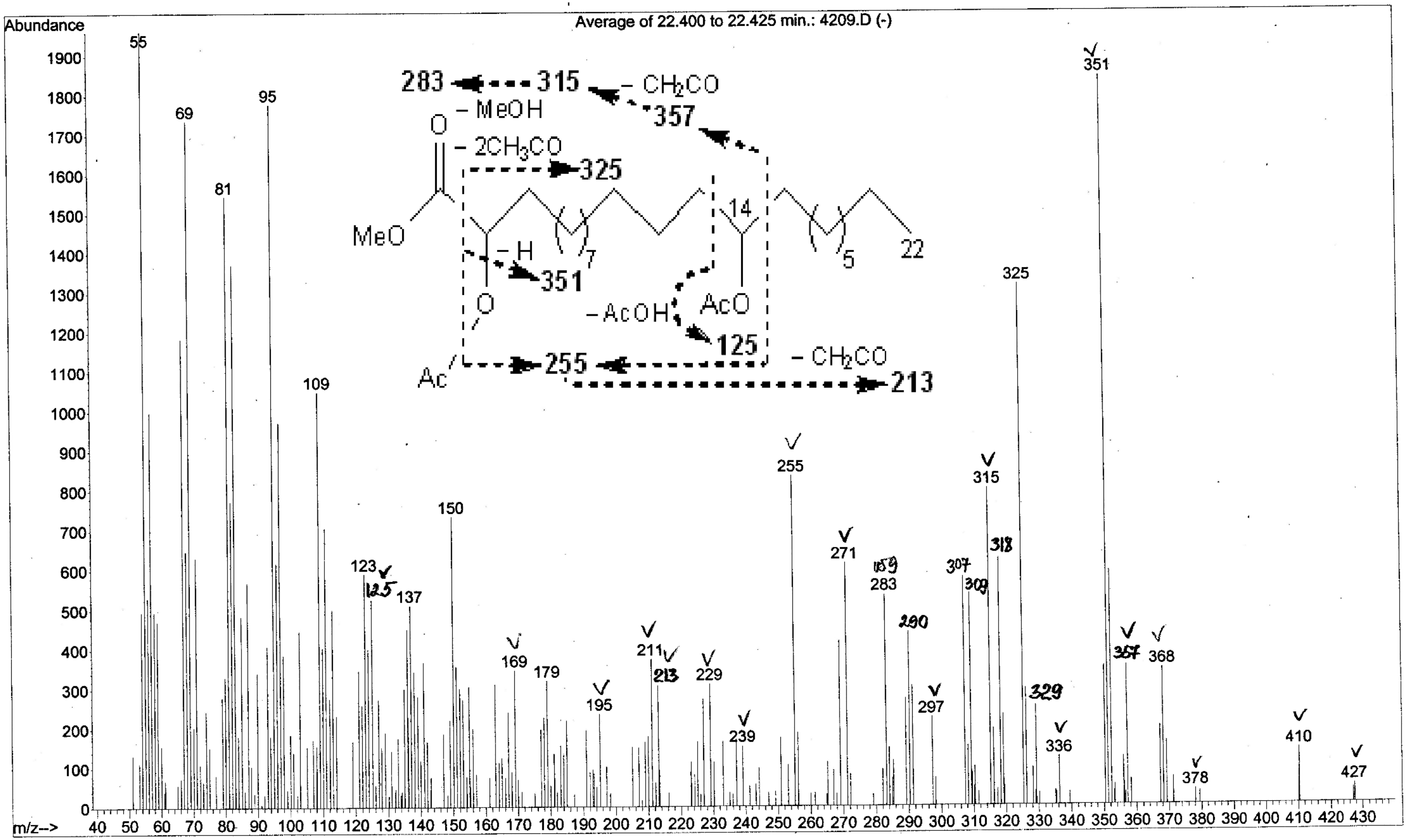


**Figure S19.** Mass spectrum of a sample enriched in the methyl ester of 2-acetyloxy $C_{22}$ acid with an additional acetyloxy group in the (*n*–9) position.

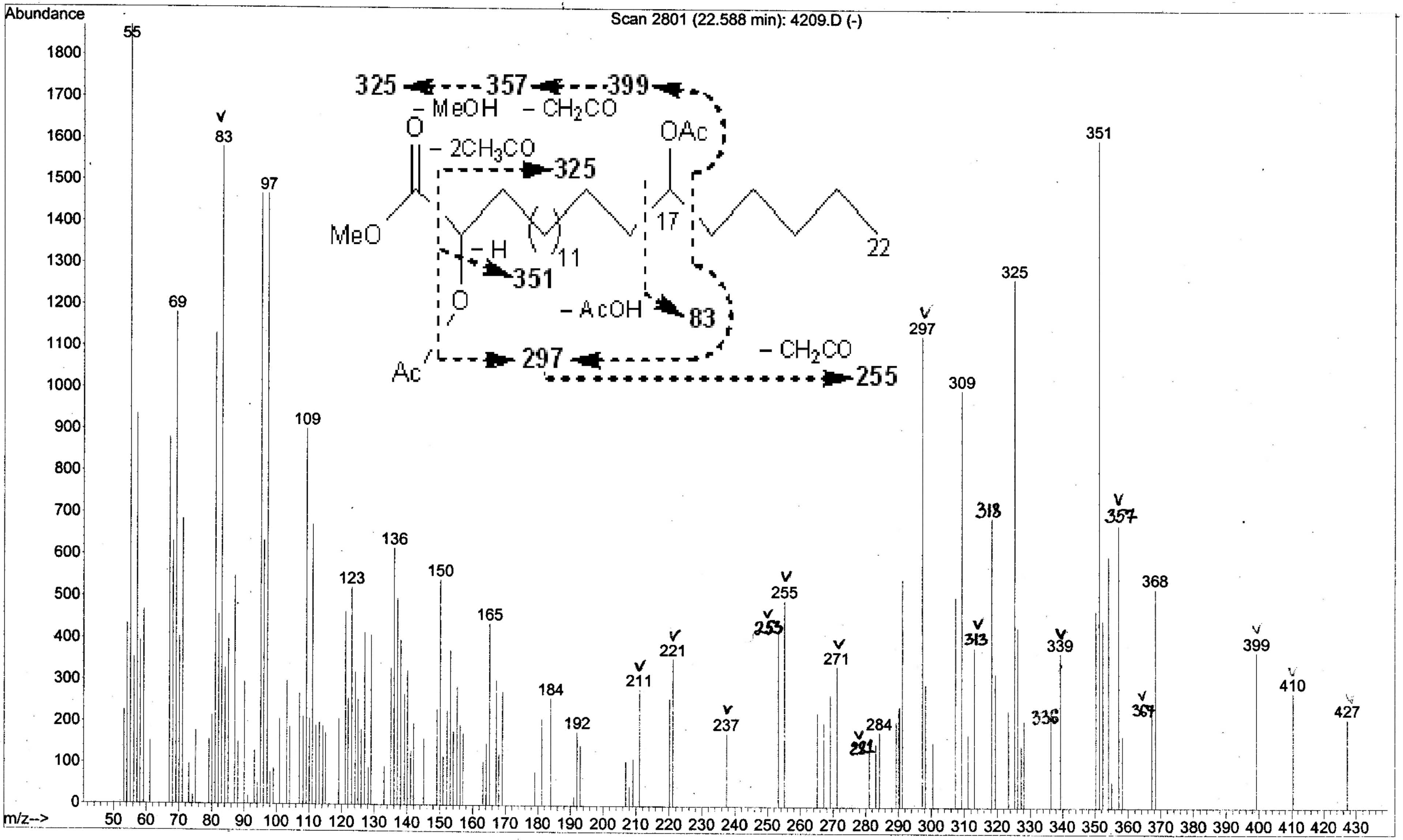


**Figure S20.** Mass spectrum of a sample enriched in the methyl ester of 2-acetyloxy $C_{22}$ acid with an additional acetyloxy group in the ($n$–6) position.

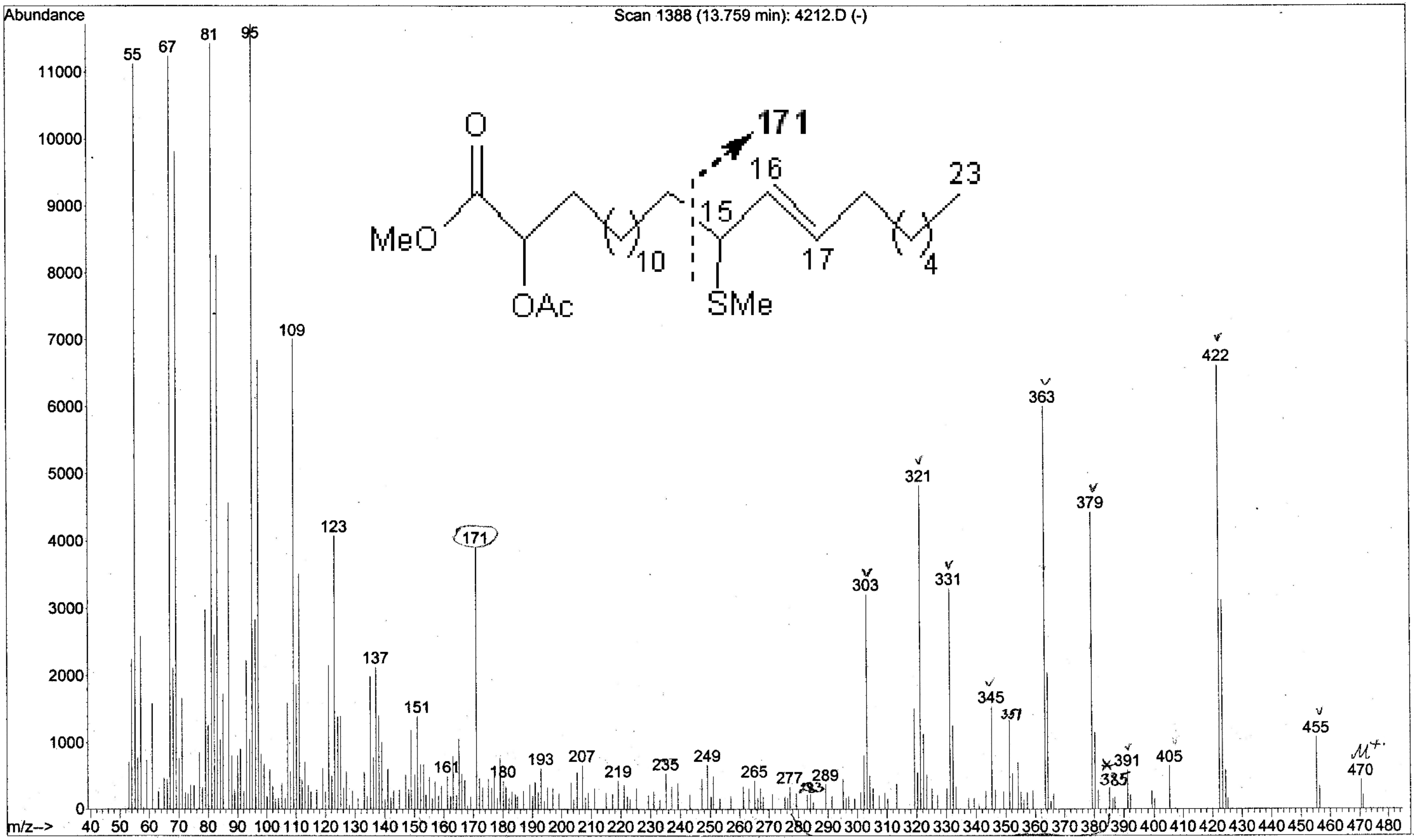


**Figure S21.** Mass spectrum of a sample enriched in the methyl ester of 2-acetyloxy $C_{23}$ acid with an allylic methylthio group in the (*n*–9) position.

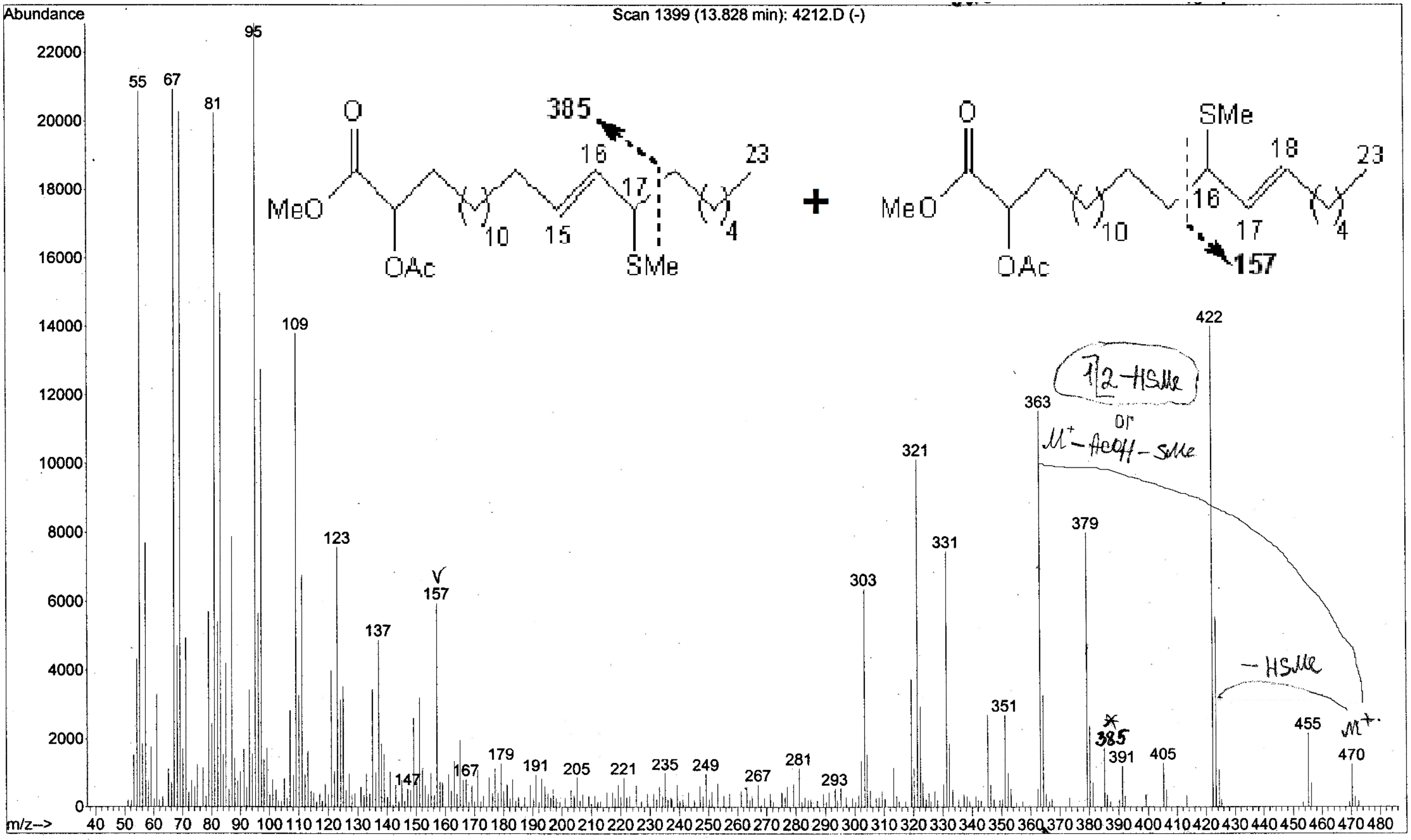


**Figure S22.** Mass spectrum of a sample enriched in the methyl esters of 2-acetyloxy $C_{23}$ acids with an allylic methylthio group in the (*n*–7) or (*n*–8) positions.

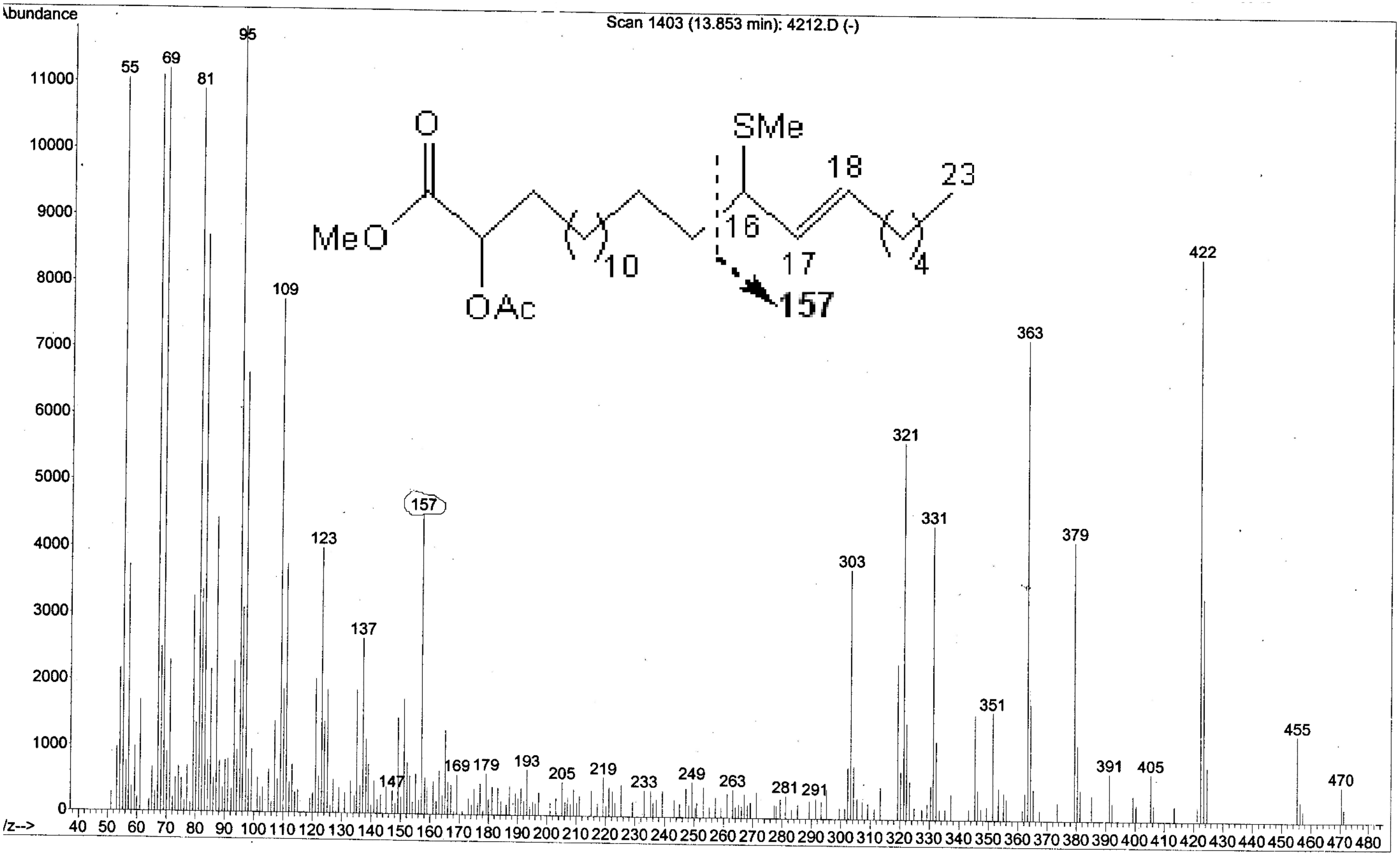


**Figure S23.** Mass spectrum of a sample enriched in the methyl ester of 2-acetyloxy $C_{23}$ acid with an allylic methylthio group in the ($n$–8) position.

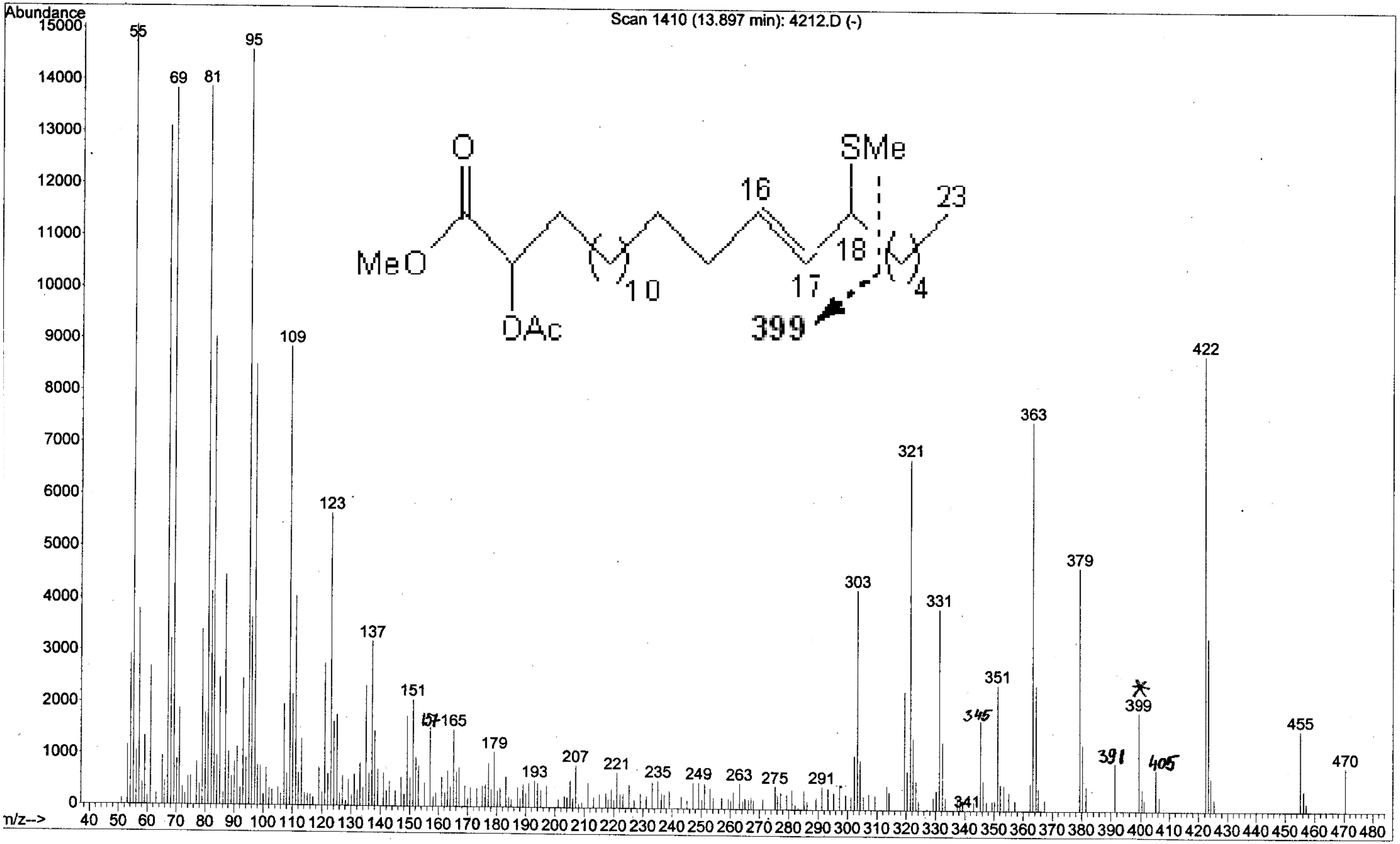


**Figure S24.** Mass spectrum of a sample enriched in the methyl ester of 2-acetyloxy $C_{23}$ acid with an allylic methylthio group in the (*n*–6) position.

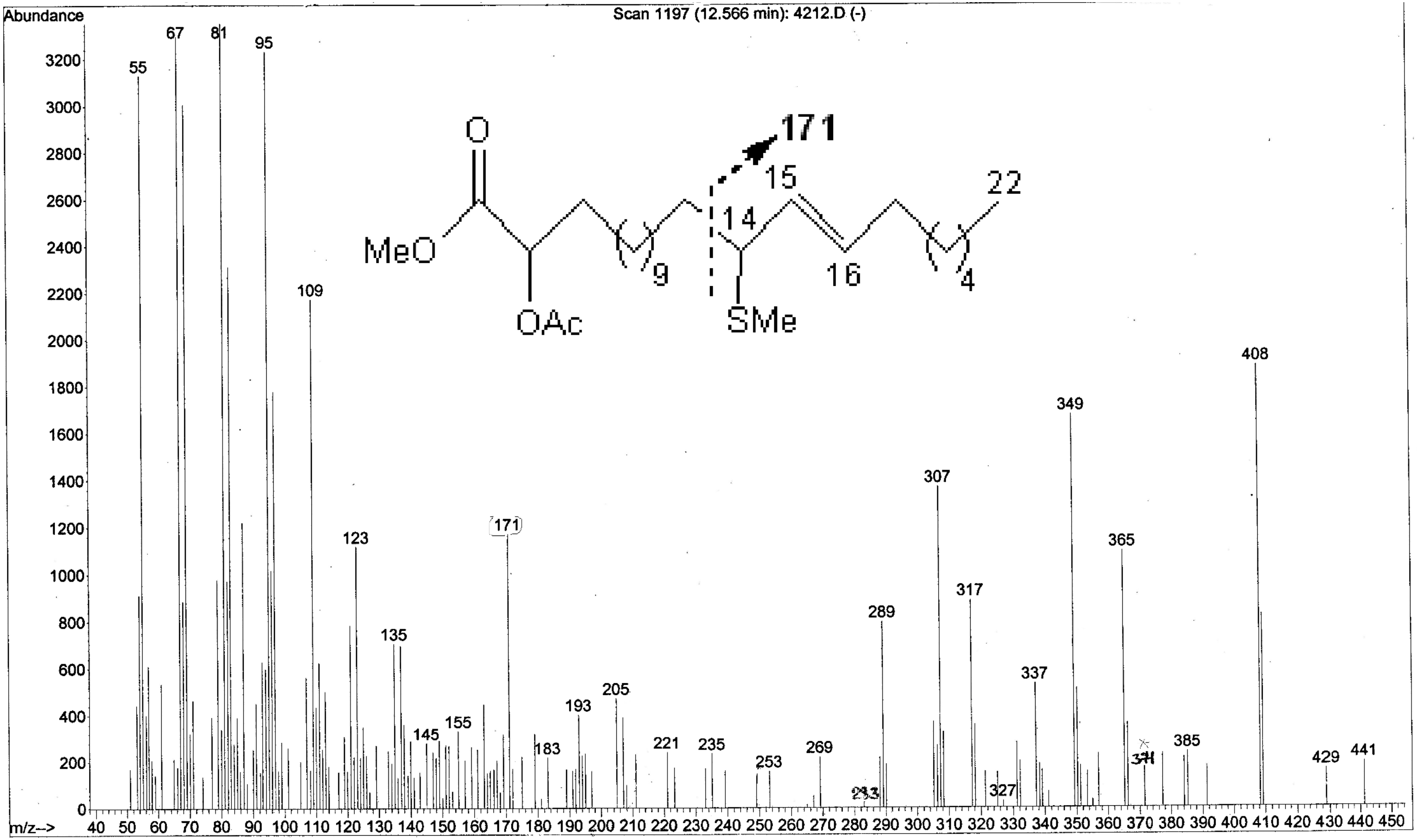


**Figure S25.** Mass spectrum of a sample enriched in the methyl ester of 2-acetyloxy $C_{22}$ acid with an allylic methylthio group in the ($n$–9) position.

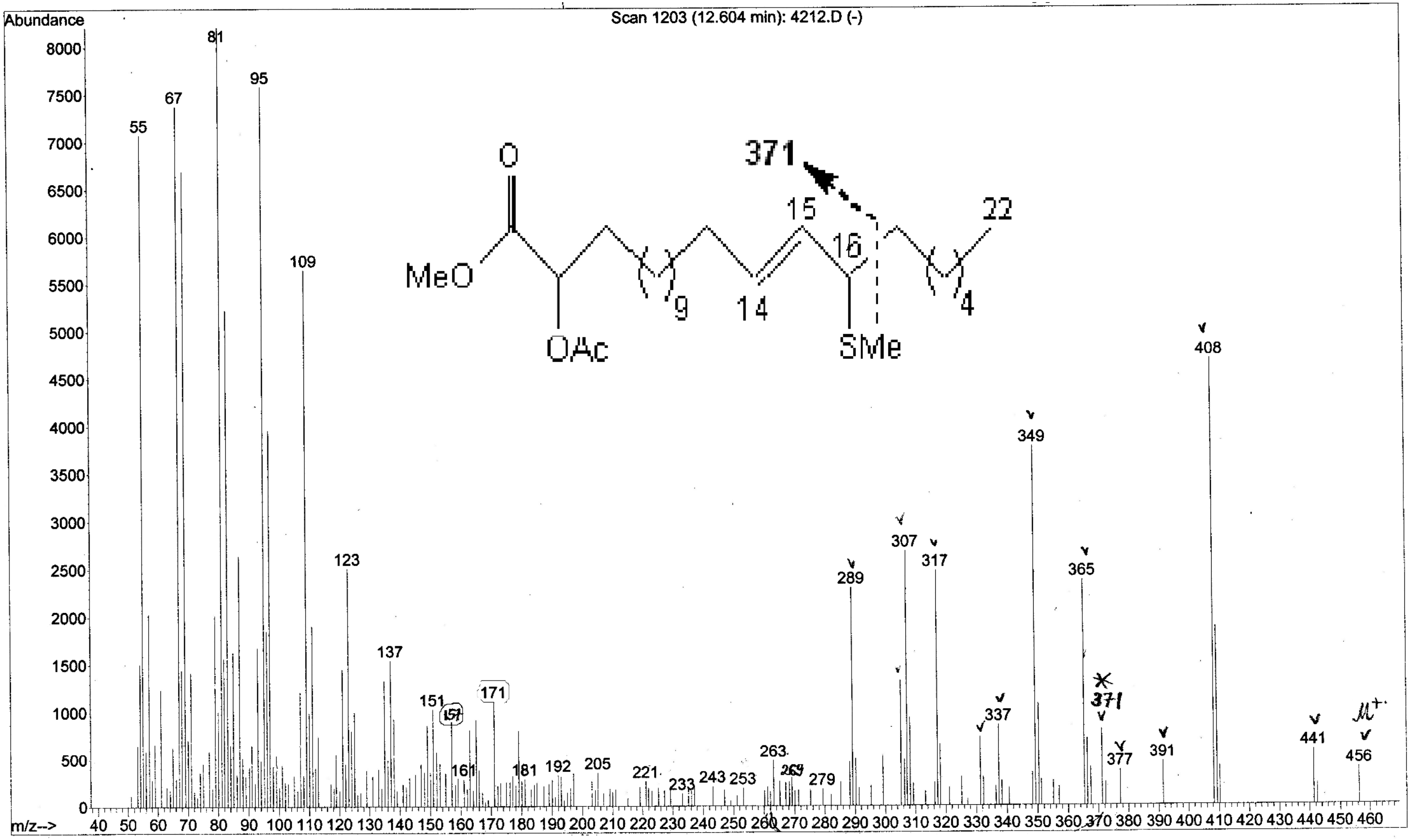


**Figure S26.** Mass spectrum of a sample enriched in the methyl ester of 2-acetyloxy $C_{22}$ acid with an allylic methylthio group in the (*n*–7) position.

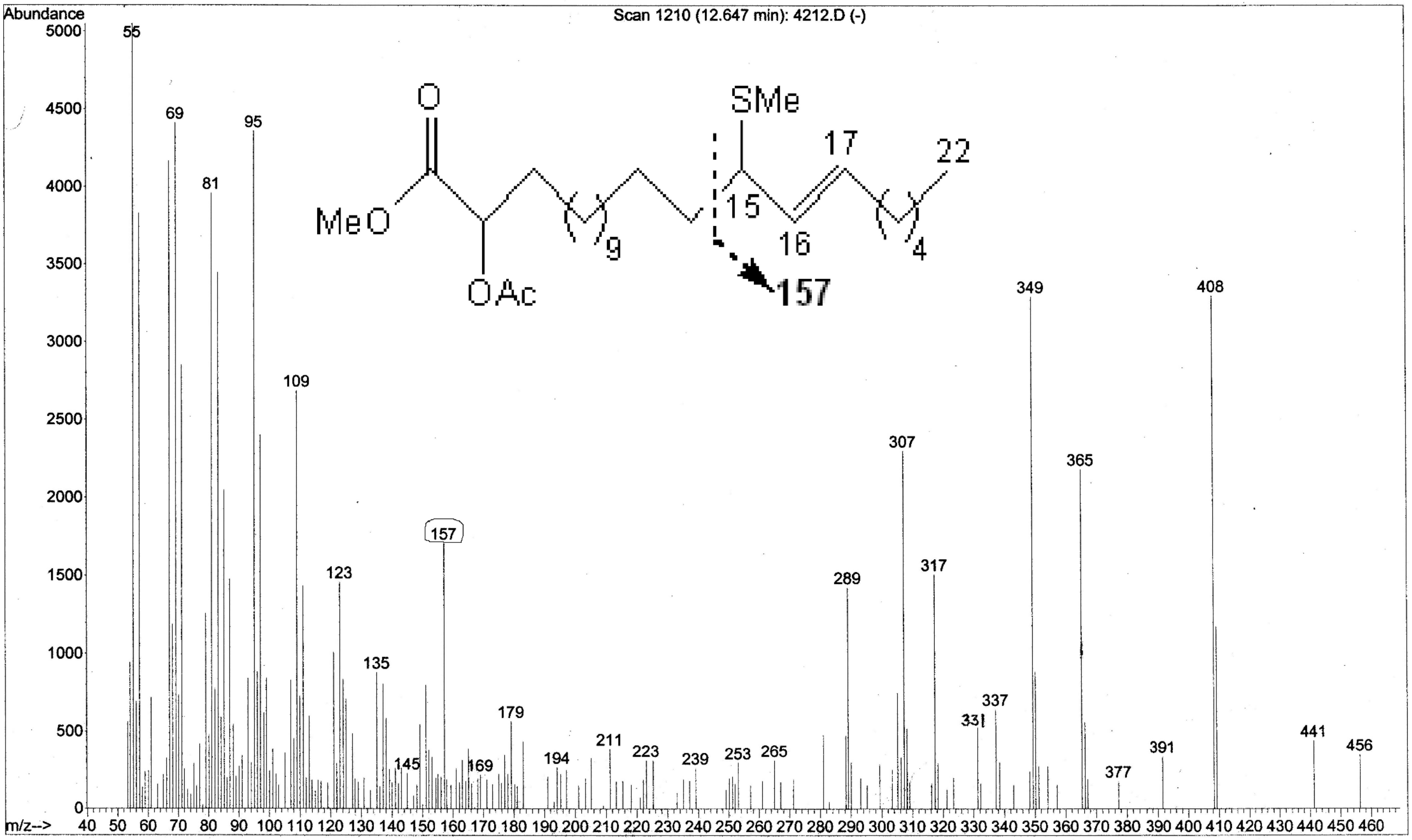


**Figure S27.** Mass spectrum of a sample enriched in the methyl ester of 2-acetyloxy $C_{22}$ acid with an allylic methylthio group in the (*n*–8) position.

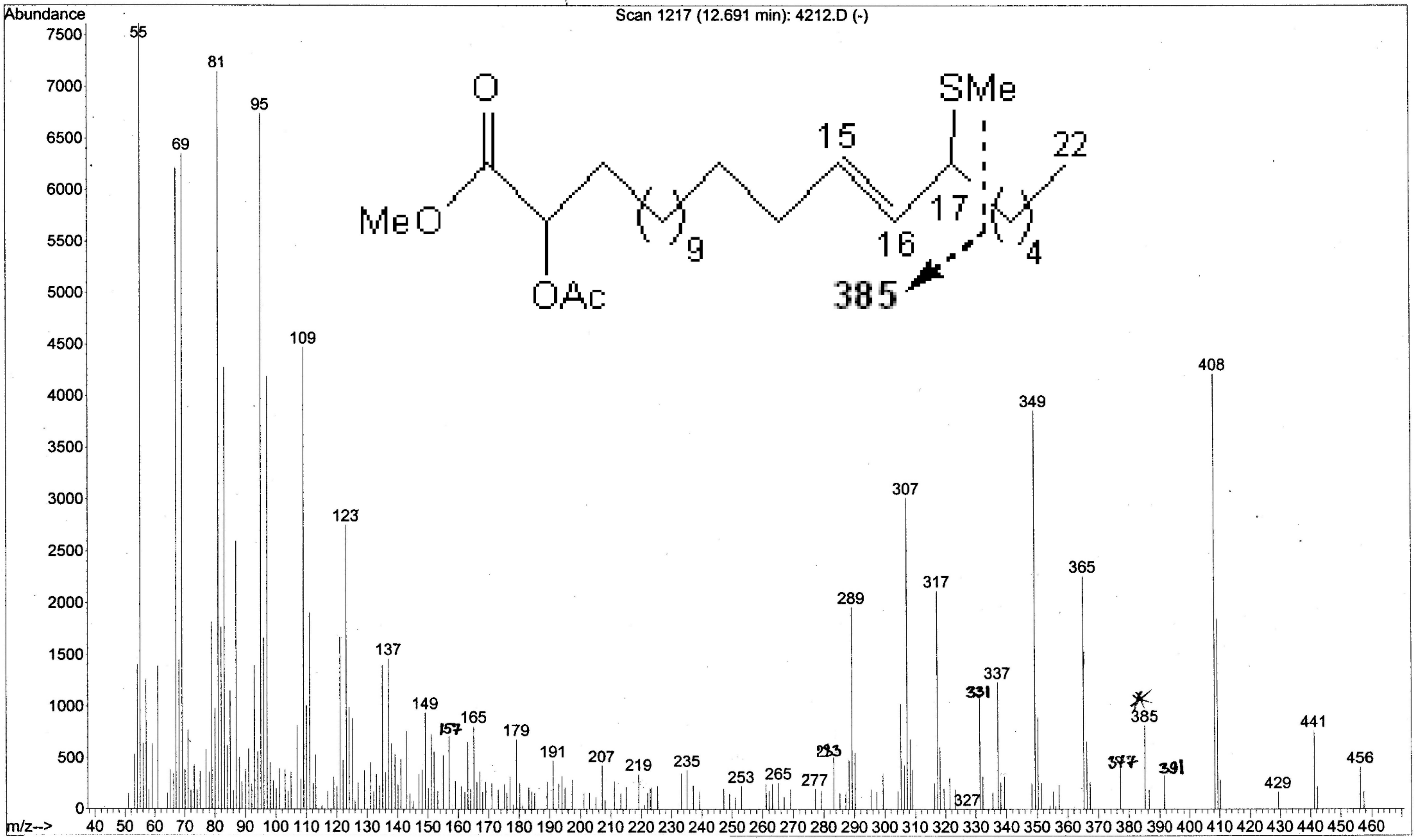


**Figure S28.** Mass spectrum of a sample enriched in the methyl ester of 2-acetyloxy $C_{22}$ acid with an allylic methylthio group in the (*n*–6) position.

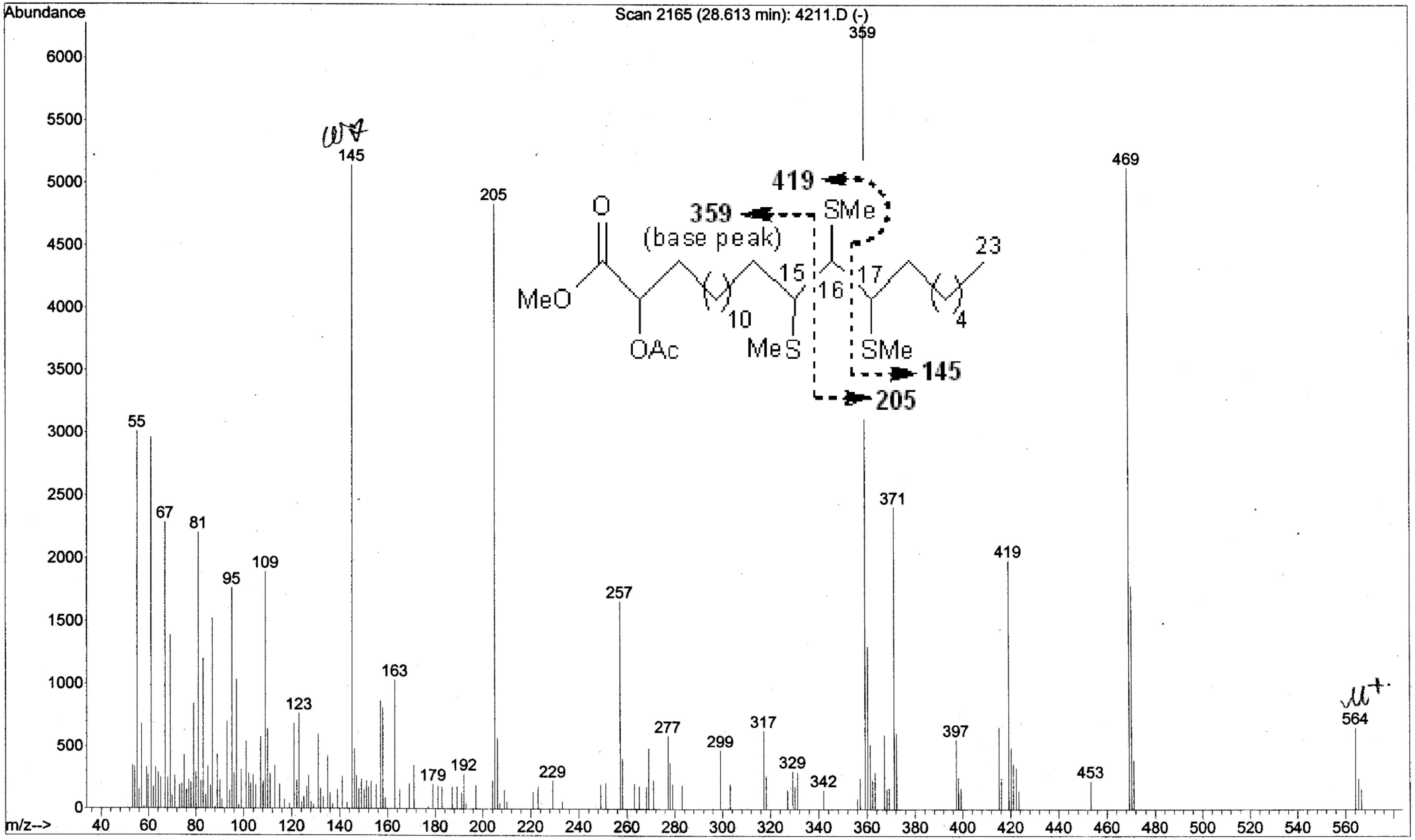


**Figure S29.** Mass spectrum of the methyl ester of 2-acetyloxy 15,16,17-*tris*(methylthio) $C_{23}$ acid.

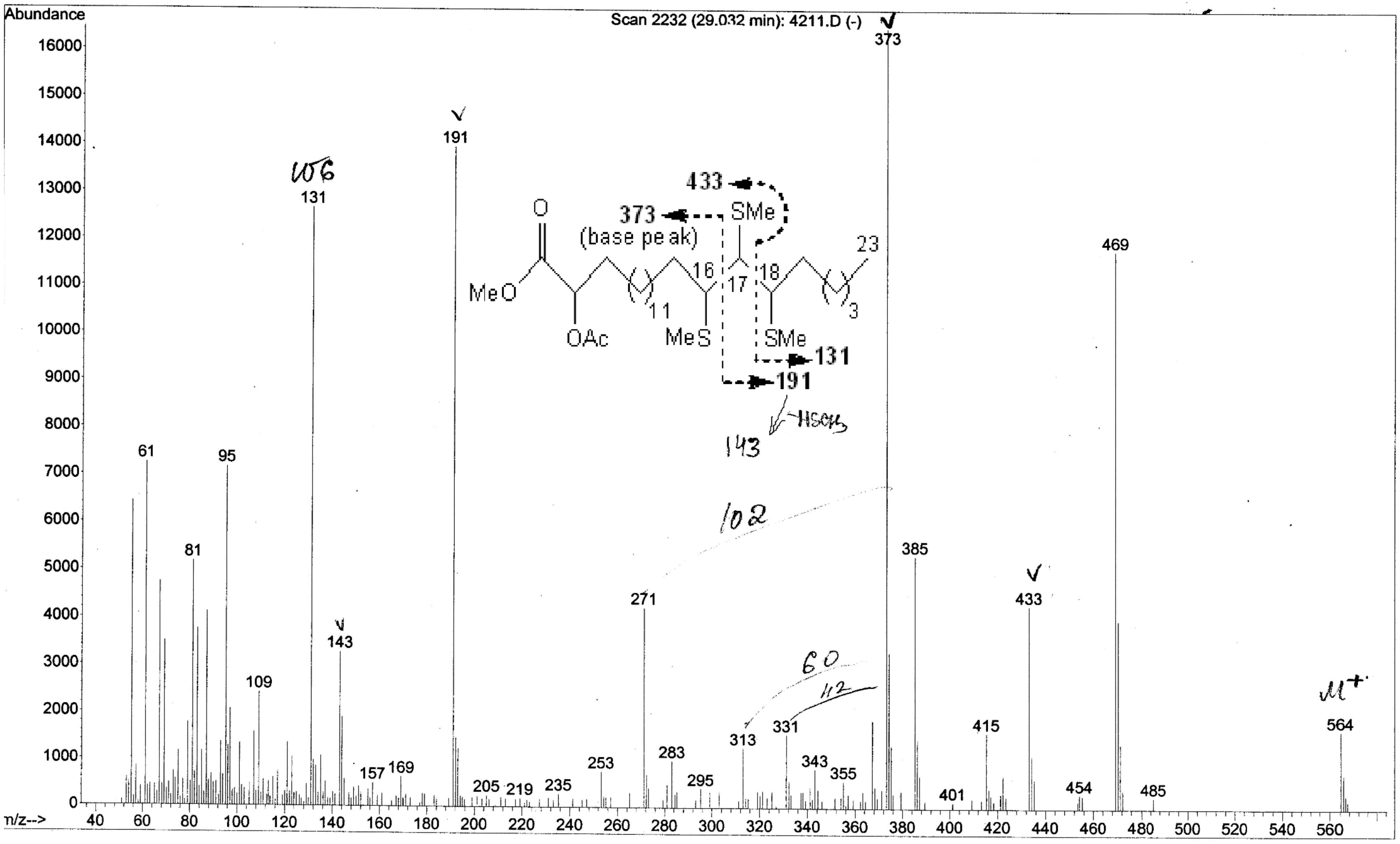


**Figure S30.** Mass spectrum of the methyl ester of 2-acetyloxy 16,17,18-*tris*(methylthio) $C_{23}$ acid.

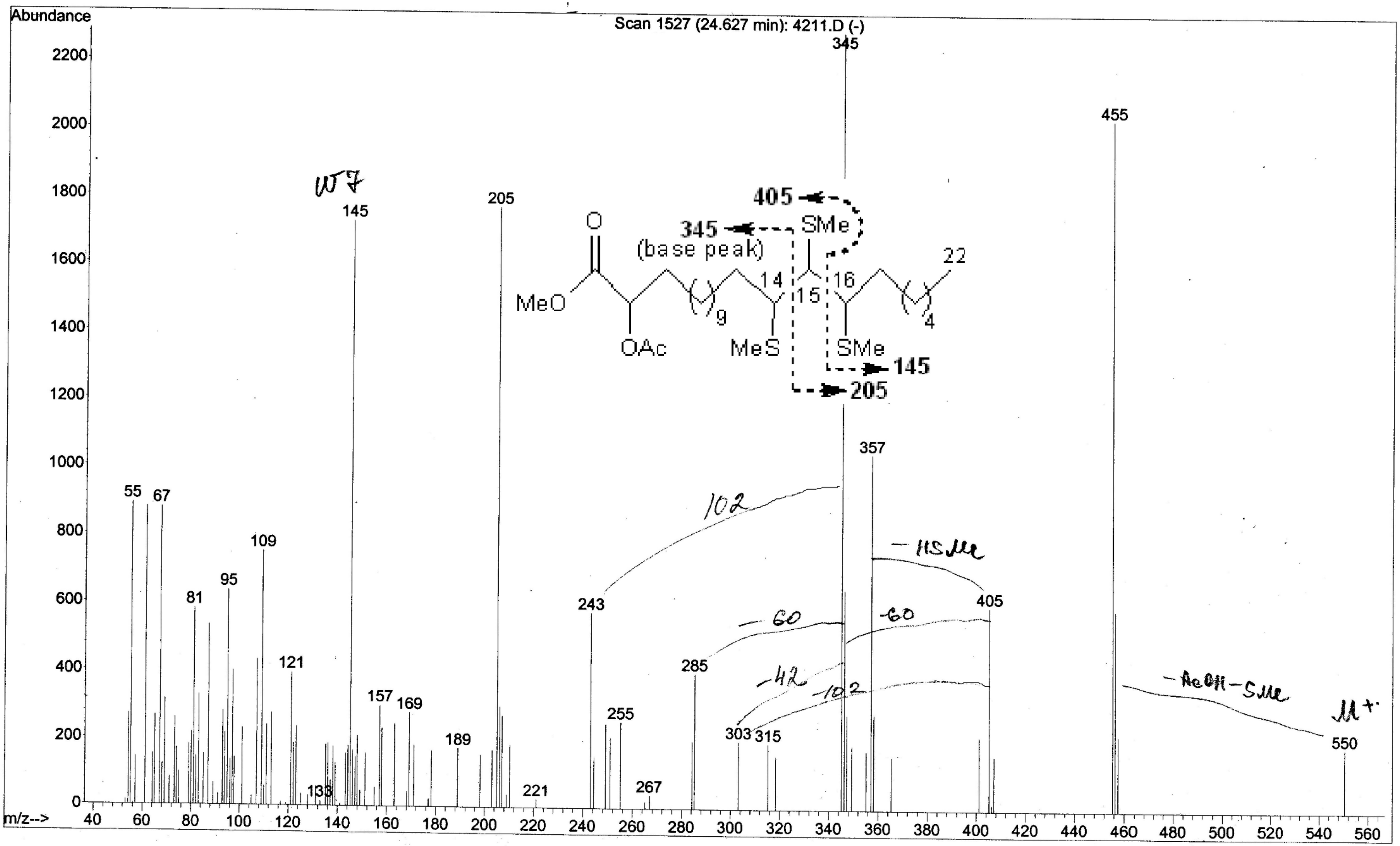


**Figure S31.** Mass spectrum of the methyl ester of 2-acetyloxy 14,15,16-*tris*(methylthio) $C_{22}$ acid.

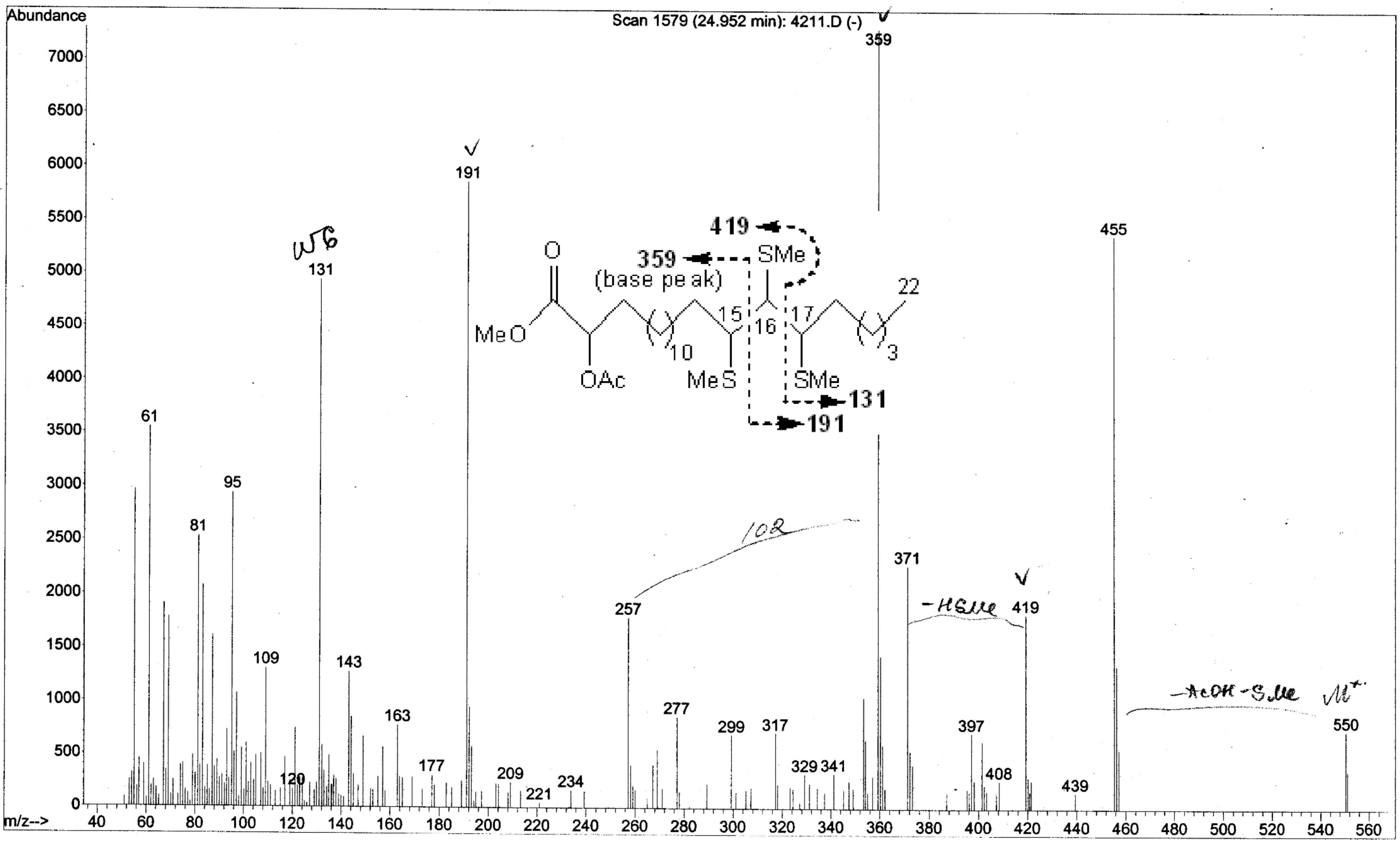


**Figure S32.** Mass spectrum of the methyl ester of 2-acetyloxy 15,16,17-*tris*(methylthio) $C_{22}$ acid.

*Allylic monohydroxylation of methyl oleate. Methylthiolation of allylic hydroxy/acetyloxy elaidates*

In a flask, $SeO_2$ (22 mg) and $CH_2Cl_2$ (0.2 mL) were stirred for 0.5 h. Then methyl oleate (50 mg in 0.3 mL $CH_2Cl_2$) was added, and the reaction mixture was stirred [46] for 3 days at room temperature. Then 10% NaCl solution (0.5 mL) was added, and the mixture was extracted with hexane (3 × 3 mL). The hexane portions were evaporated *in vacuo* yielding a yellowish oil, containing approximately 20% of allylic monohydroxy compounds according to $^1$H NMR data. A part of this product mixture was subjected to RP-HPLC purification (Agilent ZORBAX SB-C18 column, EtOH-$H_2O$, 85:15, *v/v*), and the methyl esters of 11- and 8- hydroxy elaidic acids were obtained as two HPLC fractions. Methyl 11-hydroxy elaidate, 0.4 mg: $^1$H-NMR ($CDCl_3$, 500 MHz): 5.62 (dt, 1H, *J* = 6.8, 15.4 Hz, CH-9, –**H**C=CH–CH(OH)–), 5.445 (dd, 1H, *J* = 7.1, 15.4 Hz, CH-10, –HC=C**H**–CH(OH)–), 4.03 (m, 1H, CH-11, –HC=CH–C**H**(OH)–), 3.665 (s, 3H, $H_3COCO$–), 2.30 (t, 2H, *J* = 7.5 Hz, $CH_2$-2), 2.02 (m, 2H, allylic $CH_2$-8), 1.62 (m, 2H, $CH_2$-3),1.50 (m, 1H, $CH_2$-12), 1.42–1.20 (m, –$(CH_2)_n$–), 0.89 (t, *J* = 6.9 Hz, $CH_3$-18); GC-MS *m/z* (relative intensity, %): 312 $[M]^+$ (1), 294 $[M - H_2O]^+$ (61), 263/262 $[M - H_2O - MeO]^+$/$[M - H_2O - MeOH]^+$ (15/9), 213 $[M - (CH_2)_6CH_3]^+$ (15), 195 $[M - (CH_2)_6CH_3 - H_2O]^+$ (11), 181 $[M - (CH_2)_6CH_3 - MeOH]^+$ (35), 155 $[M - CH(OH)CHCH(CH_2)_6CH_3]^+$ (52), 81 (100), 67 (96). Methyl 8-hydroxy elaidate, 0.6 mg: $^1$H-NMR ($CDCl_3$, 500 MHz): 5.625 (dt, 1H, *J* = 6.7, 15.4 Hz, CH-10, –**H**C=CH–CH(OH)–), 5.44 (dd, 1H, *J* = 7.1, 15.4 Hz, CH-9, –HC=C**H**–CH(OH)–), 4.025 (m, 1H, CH-8, –HC=CH–C**H**(OH)–), 3.66 (s, 3H, $H_3COCO$–), 2.30 (t, 2H, *J* = 7.5 Hz, $CH_2$-2), 2.02 (m, 2H, allylic $CH_2$-11), 1.62 (m, 2H, $CH_2$-3),1.50 (m, 1H, $CH_2$-7), 1.42–1.20 (m, –$(CH_2)_n$–), 0.88 (t, *J* = 6.9 Hz, $CH_3$-18); GC-MS *m/z* (relative intensity, %): 312 $[M]^+$ (1), 294 $[M - H_2O]^+$ (44), 263/262 $[M - H_2O - MeO]^+$/$[M - H_2O - MeOH]^+$ (10/8), 199 $[M - (CH_2)_7CH_3]^+$ (29), 181 $[M - (CH_2)_6CH_3 - MeOH]^+$ (38), 169 $[M - CH(OH)CHCH(CH_2)_7CH_3]^+$ (25), 167 $[M - (CH_2)_7CH_3 - MeOH]^+$ (59), 81 (94), 67 (100).

Methyl 11-hydroxy elaidate was treated with DMDS for 24 h (as described in Section 3.4 for the components from Part 2). According to GC-MS analyses, this treatment gave 59,2% of *bis*(methylthio) adduct, 26.8% of *mono*(methylthio) derivatives (allylic ethers 9-/11-*mono*(methylthio)octadec-10/9-enoates: *trans*-forms – 23.0%, *cis*-forms – 3.8%), and 8.0% of *tris*(methylthio) derivative (9,10,11-*tris*(methylthio)octadecanoate). The *bis*(methylthio) adduct of methyl 11-hydroxy elaidate MS: Figure S33. Overlapping methyl 9-(methylthio)octadec-10-enoate and 11-(methylthio)octadec-9-enoate, superimposed mass spectra: Figure S34. Methyl 9,10,11-*tris*(methylthio)octadecanoate MS: Figure S35. MeCN/HCl hydrolysis of this mixture gave 9/11-(methylthio)octadec-10/9-enoates (59.3%: *trans*-forms – 44.3%, *cis*-forms – 15%), their stereoisomeric 9,10,11-*tris*(methylthio) derivatives (12.4%), and octadecadienoate (11.7%).

Methyl 8-hydroxy elaidate was acetylated with $Ac_2O$ in pyridine (1:1, *v/v*, 0.4 ml; overnight). The acetylation gave methyl 8-acetyloxy elaidate and the starting material with the ratio close to 2:1 (only two thirds of the secondary alcohol were acetylated). Methyl 8-acetyloxy elaidate: $^1$H-NMR ($CDCl_3$, 500 MHz): 5.68 (dt, 1H, *J*

= 6.8, 15.4 Hz, CH-10, –**H**C=CH–CH(OAc)–), 5.36 (dd, 1H, *J* = 7.4, 15.4 Hz, CH-9, –HC=C**H**–CH(OAc)–), 5.17 (q, 1H, *J* = 7.0, CH-8, –HC=CH–C**H**(OH)–), 3.665 (s, 3H, $H_3COCO$–), 2.295 (t, 2H, *J* = 7.5 Hz, $CH_2$-2), 2.015 (m, 2H, allylic $CH_2$-11), 1.62 (m, 2H, $CH_2$-3),1.57 (m, 1H, $CH_2$-7), 1.42–1.15 (m, –$(CH_2)_n$–), 0.88 (t, *J* = 7.0 Hz, $CH_3$-18); GC-MS *m*/*z* (relative intensity, %): 354 $[M]^+$ (1), 311 $[M – CH_3CO]^+$ (8), 294 $[M – AcOH]^+$ (49), 279 $[M – CH_3CO – MeOH]^+$ (7), 263/262 $[M – AcOH – MeO]^+$/$[M – AcOH – MeOH]^+$ (19/15), 211 $[CH(OCOCH_3)CHCH(CH_2)_7CH_3]^+$ (4), 199 $[M – CH_2CO – (CH_2)_7CH_3]^+$ (29), 169 $[CH(OH)CHCH(CH_2)_7CH_3]^+$ (36), 167 $[M – CH_2CO – (CH_2)_7CH_3 – MeOH]^+$ (21), 150/149 $[M – (CH_2)_7CH_3 – AcOH – MeO]^+$/$[M – (CH_2)_7CH_3 – AcOH – MeOH]^+$ (19/16), 67 (100). Methyl 8-acetyloxy elaidate was divided into two parts that were treated with DMDS for 24 h and for 4 days. According to GC-MS analyses, the former treatment did not give any derivatives of the starting acetylated compound, but the latter longer-term treatment gave 8-(methylthio)octadec-9-enoate and 10-(methylthio)octadec-8-enoate (61.5%: *trans*-forms – 55.8%, *cis*-forms – 5.7%) and 8,9,10-*tris*(methylthio)octadecanoate (25.4%). Overlapping methyl 8-(methylthio)octadec-9-enoate and 10-(methylthio)octadec-8-enoate, superimposed mass spectra: Figure S36. Methyl 8,9,10-*tris*(methylthio)octadecanoate, MS: Figure S37.

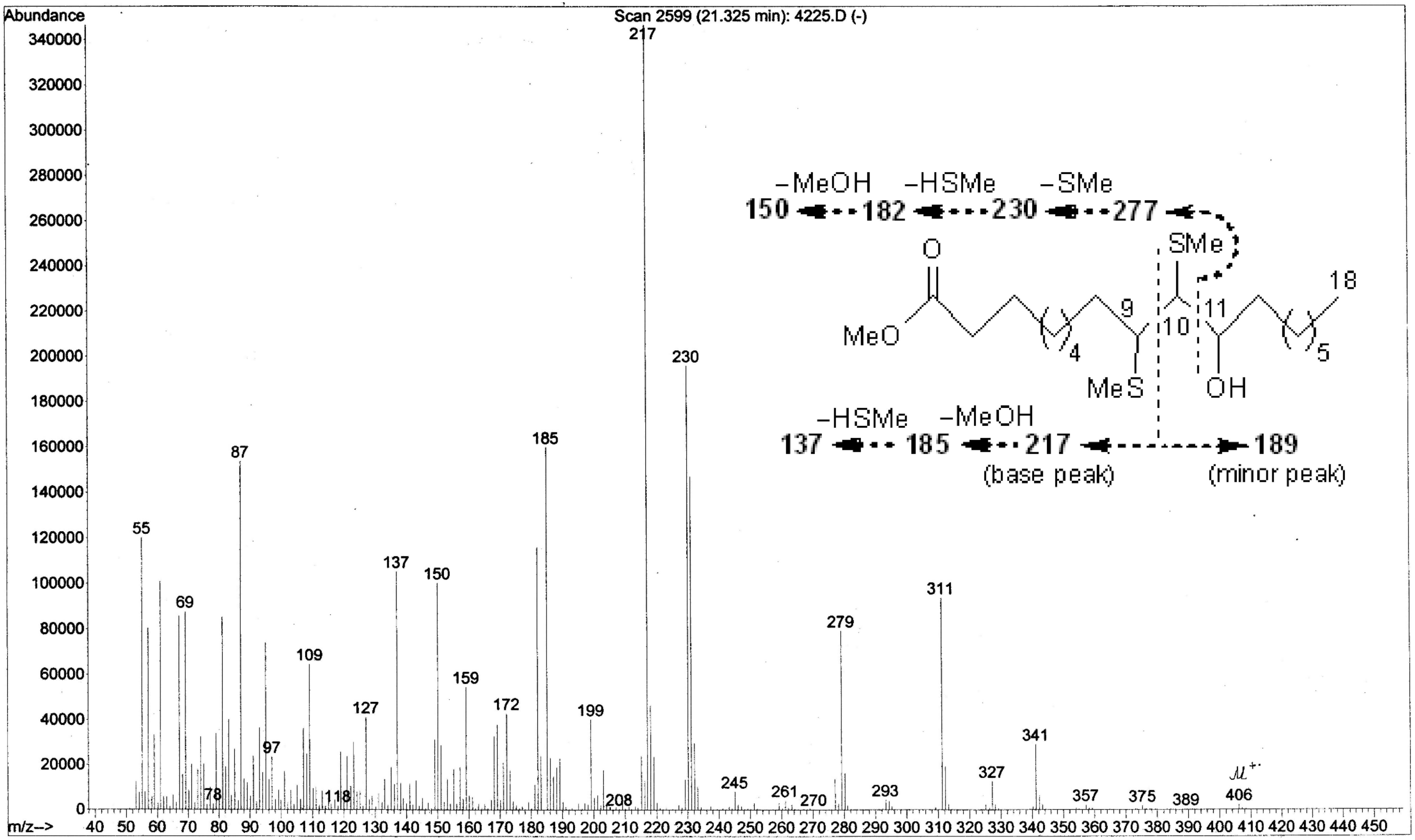


**Figure S33.** Mass spectrum of the *bis*(methylthio) adduct of methyl 11-hydroxy elaidate.

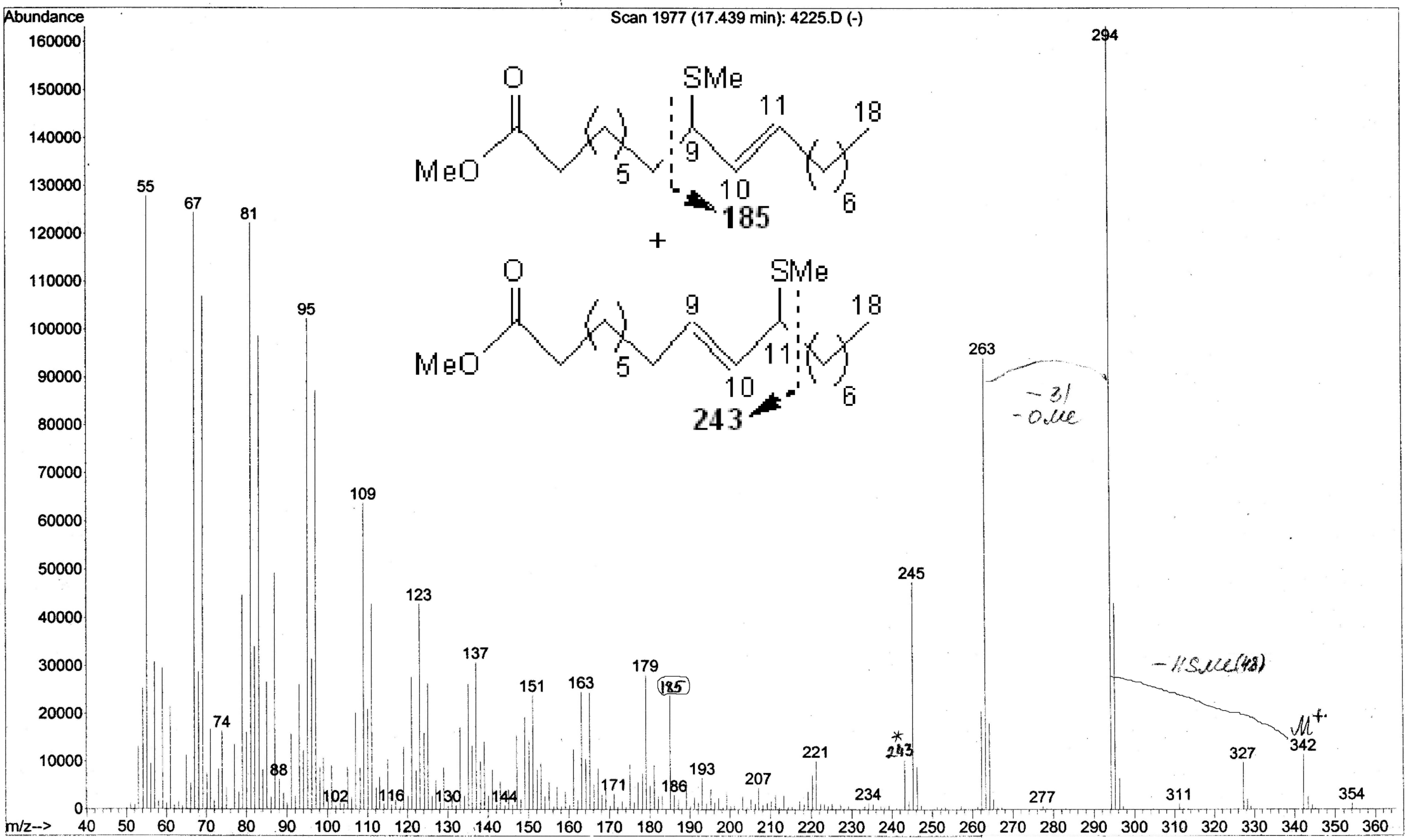


**Figure S34.** Superimposed mass spectra of overlapping methyl 9-(methylthio)octadec-10-enoate and 11-(methylthio)octadec-9-enoate.

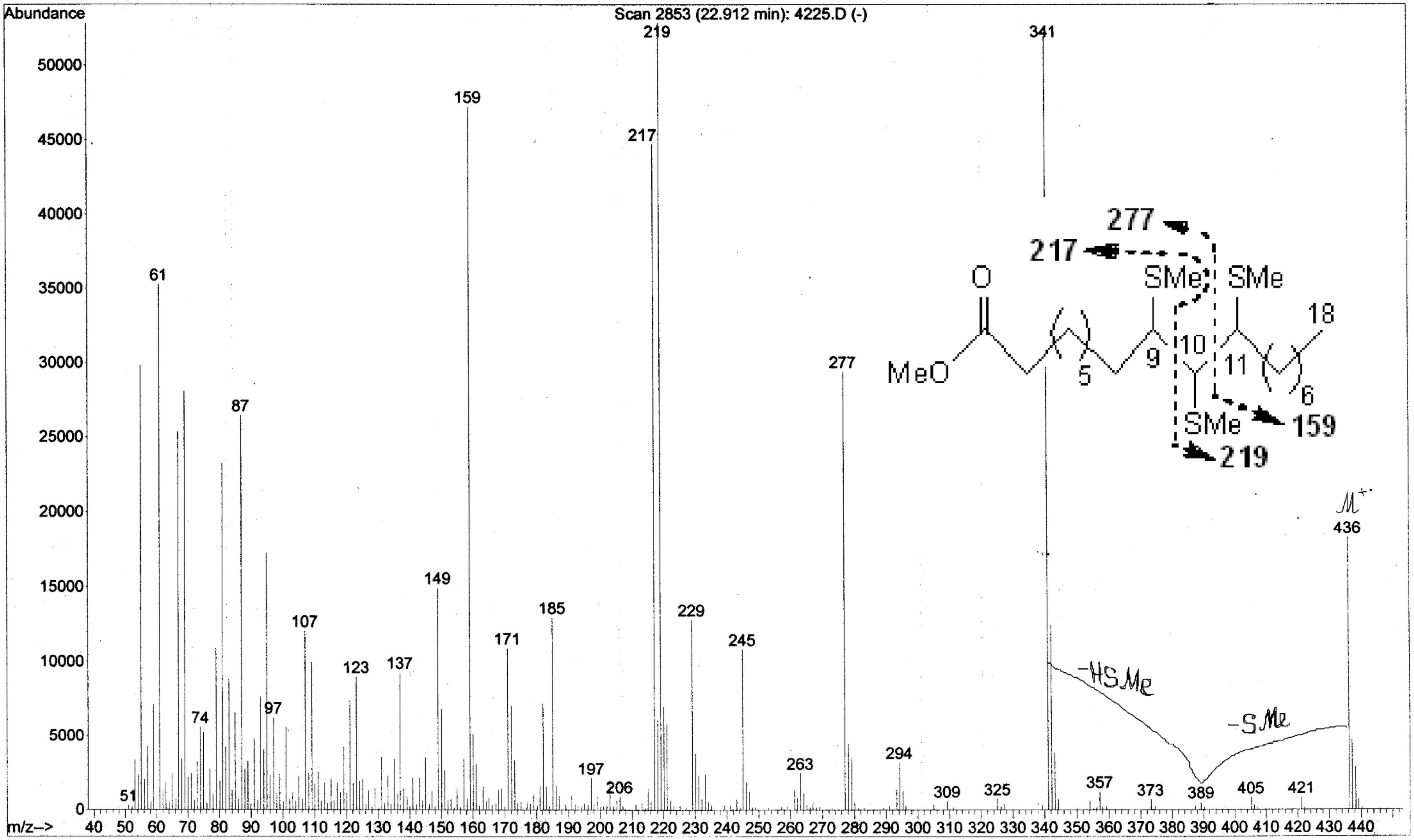


**Figure S35.** Mass spectrum of methyl 9,10,11-*tris*(methylthio)octadecanoate.

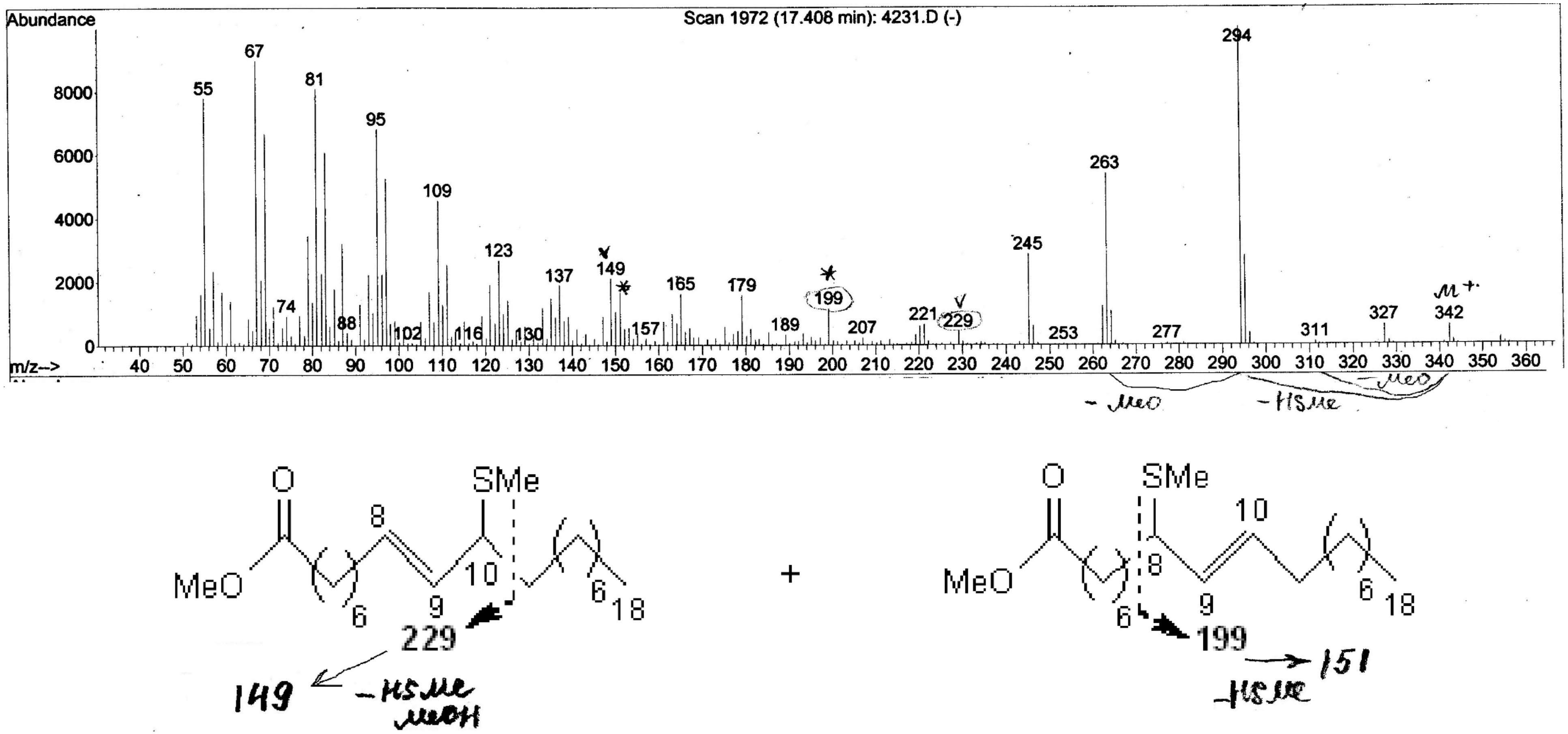


**Figure S36.** Superimposed mass spectra of overlapping methyl 10-(methylthio)octadec-8-enoate and 8-(methylthio)octadec-9-enoate.

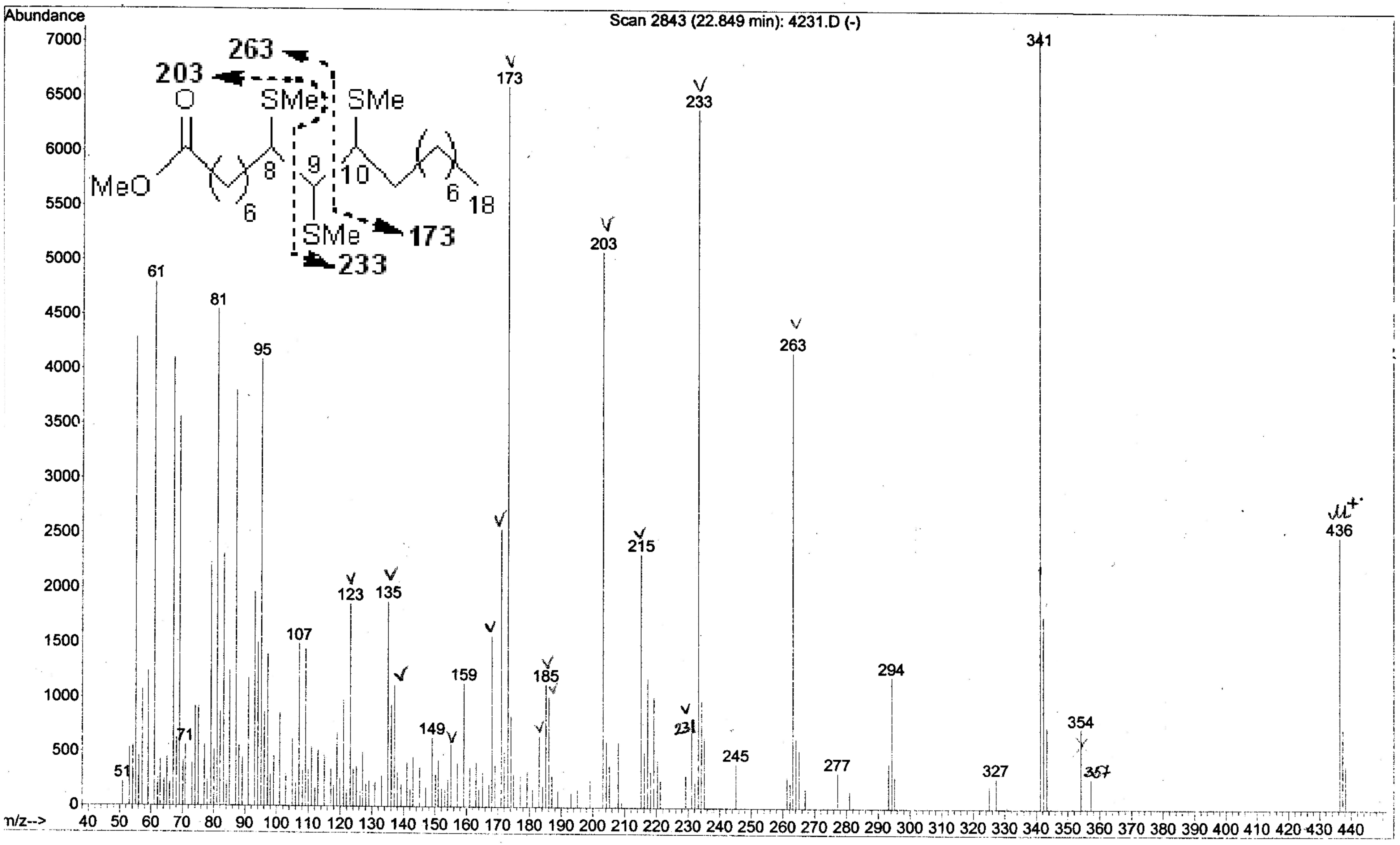


**Figure S37.** Mass spectrum of methyl 8,9,10-*tris*(methylthio)octadecanoate.
